# Two π‐Electrons Make the Difference: From BODIPY to BODIIM Switchable Fluorescent Dyes

**DOI:** 10.1002/chem.201905344

**Published:** 2020-01-09

**Authors:** Hadi Dolati, Lisa C. Haufe, Lars Denker, Andreas Lorbach, Robin Grotjahn, Gerald Hörner, René Frank

**Affiliations:** ^1^ Technische Universität Braunschweig Institute of Inorganic and Analytical Chemistry Hagenring 30 38106 Braunschweig Germany; ^2^ Universität Konstanz Fachbereich Chemie Universitätsstrasse 10 78464 Konstanz Germany; ^3^ Institut für Chemie, Theoretische Chemie—Quantenchemie TU Berlin Strasse des 17. Juni 135 10623 Berlin Germany; ^4^ Permanent address: Anorganische Chemie IV Universität Bayreuth Universitätsstrasse 30 95440 Bayreuth Germany

**Keywords:** bisimidazoles, BODIIM, BODIPY, fluorescent dyes, switchable fluorescence

## Abstract

(aza‐)BODIPY dyes (boron dipyrromethene dyes) are well‐established fluorophores due to their large quantum yields, stability, and diversity, which led to promising applications including imaging techniques, sensors, organic (opto)electronic materials, or biomedical applications. Although the control of the optical properties in (aza‐)BODIPY dyes by peripheral functional groups is well studied, we herein present a novel approach to modify the 12 π‐electron core of the dipyrromethene scaffold. The replacement of two carbon atoms in the β‐position of a BODIPY dye by two nitrogen atoms afforded a 14 π‐electron system, which was termed BODIIM (boron diimidazolylmethene) in systematic analogy to the BODIPY dyes. Remarkably, the BODIIM dye was obtained with a BH_2_‐rigidifying entity, which is currently elusive and highly sought after for the BODIPY dye class. DFT‐Calculations confirm the [12+2] π‐electron relationship between BODIPY and BODIIM and reveal a strong shape correlation between LUMO in the BODIPY and the HOMO of the BODIIM. The modification of the π‐system leads to a dramatic shift of the optical properties, of which the fluorescent emission is most noteworthy and occurs at much larger Stokes shift, that is, ≈500 cm^−1^ in BODIPY versus >4170 cm^−1^ in BODIIM system in all solvents investigated. Nucleophilic reactivity was found at the *meso*‐carbon atom in the formation of stable borane adducts with a significant shift of the fluorescent emission, and this behavior contrasts the reactivity of conventional BODIPY systems. In addition, the reverse decomplexation of the borane adducts was demonstrated in reactions with a representative N‐heterocyclic carbene to retain the strongly fluorescent BODIIM compound, which suggests applications as fully reversible fluorescent switch.

## Graphical abstract

**Imidazole‐based dyes**: The chromophoric 12 π‐electron system in BODIPY fluorescent dyes is modified to give a 14 π‐electron core. The novel BODIIM fluorescent dye shows surprising properties, which are complementary to BODIPY dyes.

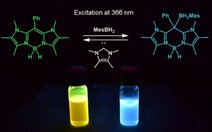

## Introduction

**Scheme 1:**
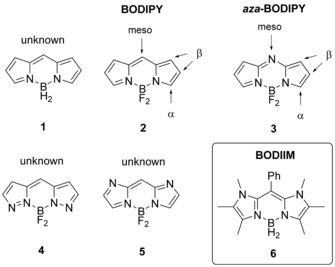
Structures of compounds **1**–**6**.

BODIPY dyes (boron dipyrromethenes) constitute an important class of fluorophores, which can be considered as boron chelates with a dipyrrin entity^1^ and have attracted broad interest as photoresponsive compounds, in particular as efficient fluorescent dyes (Scheme 1). The success of BODIPYs is due to their excellent optical key properties, which include strong absorbance and narrow‐band fluorescence at high quantum yields combined with excellent photostability in most organic solvents. The Stokes shifts of BODIPYs are low (typically about 500 cm^−1^), and absorbance and emission maxima are commonly observed between 500–550 nm. The extraordinarily rich chemistry of BODIPY fluorophores allows for high functional‐group tolerance and procedures of post modification,^2^ which plays a crucial role in the development of materials for imaging techniques,^3^ sensors,^4^ organic (opto)electronic applications,^5^ biomedical applications,^6^ photodynamic therapy,^7^ and sensitizers.^8^ Although the parent BODIPY **1** containing a BH_2_‐complexed dipyrrin entity is currently unknown, the unsubstituted BF_2_‐functionalized analogue **2** was obtained with considerable effort.^9^ In contrast, derivatives of **2** were readily synthesized as early as 1968 by Treibs and Kreuzer,^10^ and various modifications have been achieved at the *meso*‐, α‐, and β‐positions.^11^ In addition, the BF_2_ entity in derivatives of **2** is subject to facile post functionalization with a broad scope of hydrocarbyl groups.^12^

The optical properties of organic π‐systems can systematically be tuned by incorporation of hetero atoms into the scaffold. Successful application of this methodology has been demonstrated with the development of aza‐BODIPY dyes **3**, in which the *meso*‐CH unit is replaced by a nitrogen atom. In contrast to BODIPY dyes **2**, absorbance and fluorescence emission in **3** are now significantly redshifted to at least 650 nm (visible red or near‐IR region), which suggests the application of these systems in bio‐imaging procedures.^13^ Despite the intriguing results achieved with aza‐BODIPY dyes **3**, the further heteroatom incorporation was attempted but remained unachieved. In particular, compounds **4** and **5** were proposed as an extension of the concept but synthetic approaches to afford the key structures were reported to fail.^14^ Exploiting the concept of heteroatom incorporation into the π‐system of a BODIPY dye, compound **6** is presented as a recent result from our laboratories. The π‐system can be viewed as being formed by the formal replacement of two methyne entities from the β‐position in the unknown parent BODIPY **1** by N−CH_3_ groups. Due to the systematic analogy of compound **6** to BODIPY dyes we herein introduce the term boron diimidazolylmethene (BODIIM) for this type of novel compounds.

## Results and Discussion

**Scheme 2:**
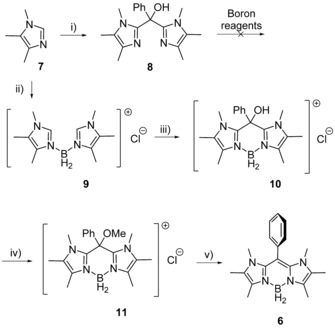
Key conditions and reagents. i) 1 equiv. *n*BuLi, THF, −30 °C, 30 min, then 0.45 equiv. benzoyl chloride, 0 °C to rt, 1 h, 65 %. ii) 0.5 equiv. BH_2_Cl⋅SMe_2_, DCM, 0 °C to rt, 15 min, then THF, 15 min, then hexanes, 95 %. iii) 2.05 equiv. *n*BuLi, THF, −78 °C to rt, overnight, then 1.05 equiv. methyl benzoate, rt, 1 h, aqueous work‐up with brine, 95 %. iv) 1.05 equiv. Na[N(SiMe_3_)_2_], THF, −78 °C to rt, 3 h, then 3 equiv. MeI, rt, 12 h, aqueous workup with brine, 96 %. v) 4 equiv. KC_8_, THF, 0 °C, 15 min, 95 %.

The synthetic access towards compound **6** was performed employing trimethyl imidazole **7** (Scheme 2).^15^ Given that BODIPY compounds are commonly synthesized from pre‐formed ligand scaffolds (dipyrromethene precursors) by the complexation to the boron reagent being the last step of the synthetic protocol, we attempted such strategy for compound **6**. Thus, the lithiation of **7** at low temperature and subsequent reaction with benzoyl chloride afforded bisimidazole compound **8**, the complexation of which was attempted with various boron reagents including BX_3_, BHX_2_⋅SMe_2_ or BH_2_X⋅SMe_2_ (X=Cl, Br).

**Figure 1:**
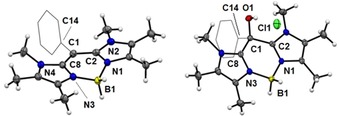
Molecular structures of compounds **6** (left) and **10** (right). The phenyl entity is represented in the wireframe model. Thermal ellipsoids are drawn at the 50 % probability level. Solvent molecules (chloroform) in the structure of **10** are omitted. Selected bond lengths (Å) and bond angles (°) for **6**: B1−N1 1.546(1), B1−N3 1.550(1), C8−N3 1.356(1), C1−C8 1.413(1), C1−C2 1.417(1), C1−C14 1.490(1), C2−N1 1.357(1), C8‐C1‐C14 121.95(9), C2‐C1‐C14 122.73(9), C2‐C1‐C8 115.17(9); for **10**: B1−N1 1.553(3), B1−N3 1.553(3), C8−N3 1.326(3), C1−C8 1.512(3), C1−C2 1.513(3), C1−C14 1.534(3), O1−C1 1.412(3), C2−N1 1.323(3), C8‐C1‐C14 107.90(18), C2‐C1‐C14 109.06(18), C2‐C1‐C8 108.56(18).

However, in all cases unselective reactions were observed giving rise to several signals in the ^11^B NMR spectra. This behavior may be rationalized by the presence of the hydroxyl group in **8**, which prevents selective reactions due to the oxophilic nature of the boron reagents employed. Therefore, boron was introduced in the first step of the synthetic sequence. The reaction of trimethyl imidazole **7** with BH_2_Cl⋅SMe_2_ afforded the bisimidazol‐functionalized boronium compound **9** in high yield, in which the carbon bridge was readily introduced by lithiation of **9** at the C2‐positions and the subsequent reaction with methyl benzoate. The resulting alcohol **10** was converted to the respective methyl ether **11** by deprotonation of the hydroxyl group followed by addition of methyl iodide. The reduction of ether **11** with potassium graphite KC_8_ afforded the novel BODIIM compound **6** as a yellowish, fluorescent solid. All compounds were fully characterized including multinuclear NMR spectroscopy, elemental analysis and in the case of **6**, **8**, and **10** by X‐ray crystallography; for **6** and **10** see Figure 1, for **8** see the Supporting Information.

The reduction of ether **11** significantly changes the geometry of the carbon bridge C1 from a fourfold‐coordinate carbon atom (in **10** and **11**) to threefold coordination (in **6**). The bonds C1−C2 and C1−C8 undergo significant contraction upon reduction with an average bond length C1−C2/C8 (1.513(3) in **10** vs. 1.415(1) Å in **6**). This behavior is in line with an increase of the double‐bond character at the central carbon C1. In the molecular structures of **6** and **10** the scaffold atoms N1‐C2‐C1‐C8‐N3 were found to span a plane with only minor deviations from the idealized geometry. Although in precursor **10** the BH_2_‐entity is well aligned within the plane with a slight inclination of only 1.56(2)° the distortion from the planarity of the BH_2_‐entity in compound **6** is much higher with an angle of 17.76(2)°, see the Supporting Information.

**Figure 2:**
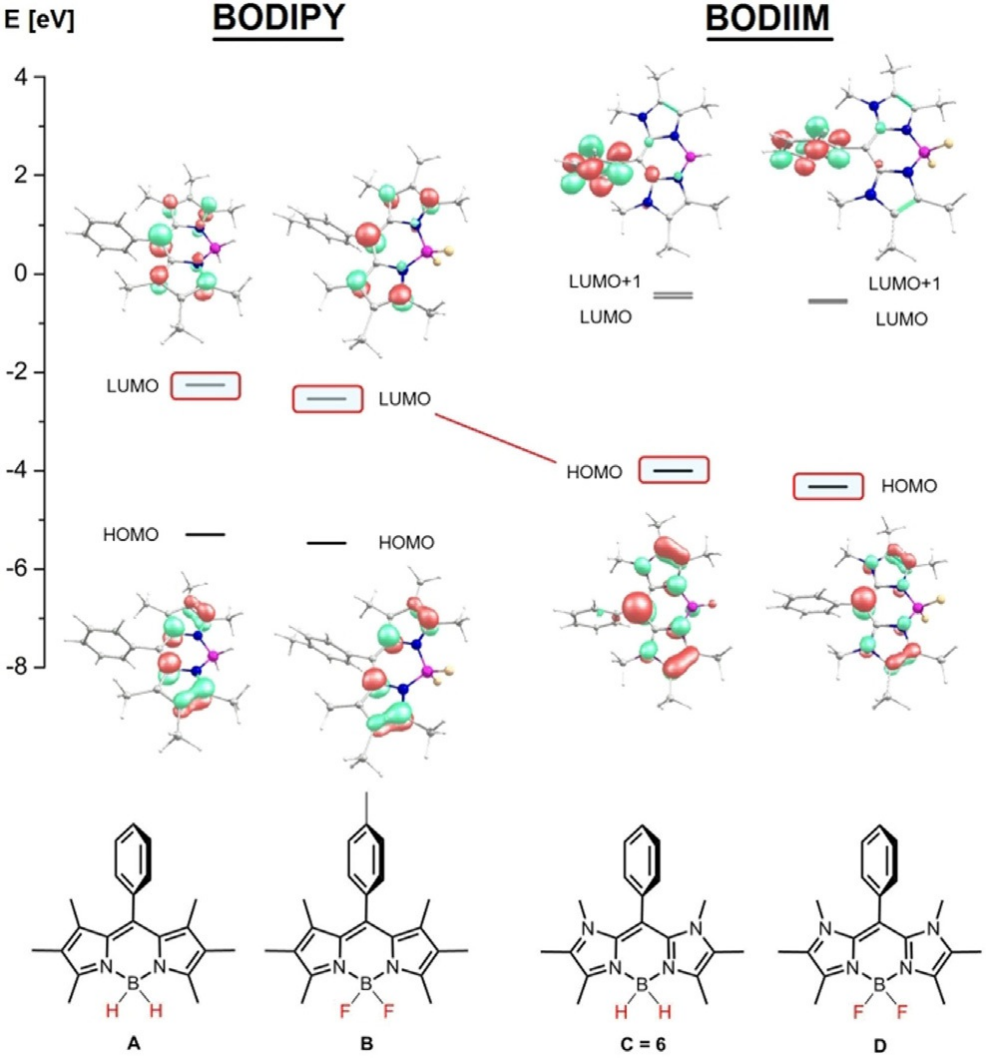
Selected molecular orbitals for BODIPY (**A**, **B**) and BODIIM (**C**, **D**) systems as obtained by DFT calculations (B3LYP‐D3/TZVP/COSMO(THF)). The addition of 2 π‐electrons to the LUMO in BODIPY systems retains the HOMO in the novel BODIIM system with preservation of the characteristic atomic orbital contributions.

A comparison of BODIPY systems **2** with the novel BODIIM compound **6** reveals remarkable differences. Although compound **6** is readily obtained in the parent form with a BH_2_ entity, such type of BODIPY compounds is currently elusive to the best of our knowledge, and the BODIPY dye class is usually prepared as BF_2_ derivatives. Previous attempts by Piers et al. to produce the parent BH_2_‐BODIPY core from reactions of dipyrrin and BH_3_⋅SMe_2_ provide hints of the suggested species as the kinetic product in a crude mixture but its isolation as a clean material could not be demonstrated. Instead, the thermal treatment of the crude product afforded a nonfluorescent dipyrromethano borane derivative formed by hydride migration from the BH_2_ entity to the *meso*‐carbon atom.^16^ With 12 π‐electrons in the organic framework of a BODIPY system, the formal replacement of carbon atoms for two N−CH_3_ moieties leads to a BODIIM system with an increase to 14 π‐electrons. For further insight DFT calculations (for computational details, see the Supporting Information) were performed for BODIPY type compounds **A** (BH_2_, hypothetical system) and **B** (BF_2_, reference system)^17^ as well as for the novel BODIIM type compounds **C** (BH_2_, representing compound **6**) and **D** (BF_2_, hypothetical analogue of **6**; Figure 2). Optimized structures of **B** and **6** closely mimic the experimental metrics (bond length deviations <1 pm) and render the proposed structures of hypothetic **A** and **D** highly reliable (see Table S1, Supporting Information). With these systems effects of boron substitution (BF_2_ versus BH_2_) and varied π‐electron count (12 π in BODIPYs **A**, **B** versus 14 π in BODIIMs **6**, **D**) can be studied in isolation. Inspection of the frontier orbitals of **A**–**D** proves the substitution at boron to be insignificant. In contrast, the strikingly diverging properties of BODIPY and BODIIM, both in the ground and in the excited state (see below), can be traced to altered frontier orbital character by virtue of the π‐electron count.

Although in BODIPY derivatives **A** and **B** both HOMO and LUMO are extended over the dipyrromethene backbone, this only holds for the HOMO in BODIIM compounds **C** and **D**. In contrast, the degenerate LUMO and LUMO+1 are essentially centered on the phenyl entity. A couple of equally phenyl‐centered MOs feature at much higher energies as LUMO+1 and LUMO+2 in **A** and **B**. Most strikingly, the LUMO in BODIPY systems and the HOMO in the BODIIM system appear to be related to each other and display a pronounced resemblance with a strong contribution of the carbon p‐orbital at the *meso*‐position. We conclude that the replacement of two methyne C−CH_3_ entities (**A**, **B**) for two N−CH_3_ moieties (**C**, **D**) does not simply obey a [12+2] π‐electron‐count formalism but can rather be considered as targeted structural tuning. The substitution of two carbon atoms by two nitrogen atoms within the π‐conjugated system leads to an increase of the π‐electrons by two. Thus, in a simplistic view, one would expect that the HOMO of the nitrogen‐containing compound should display strong similarities with the LUMO in the carbon derivative.

**Figure 3:**
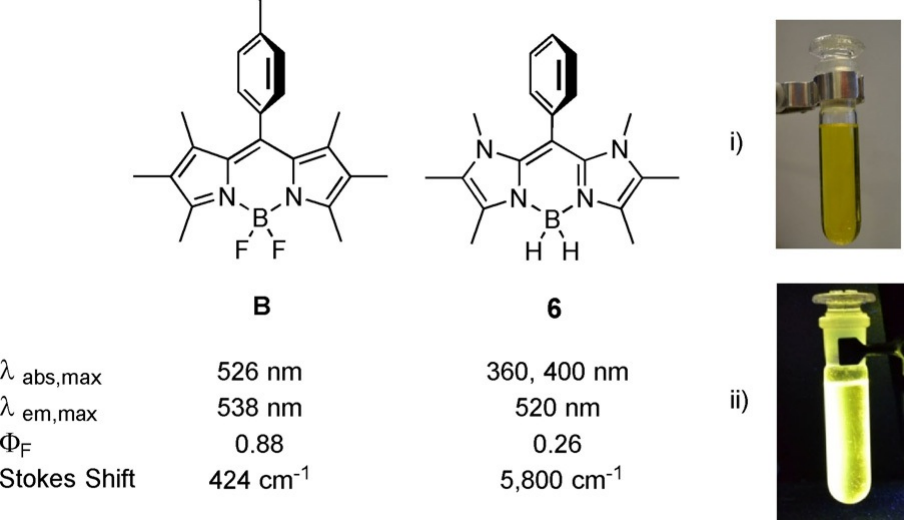
Comparison of the optical properties of BODIPY reference compound **B** (data reported in Ref. 17, recorded in toluene) and BODIIM **6** (recorded in toluene). i) Solution of compound **6** in toluene at ambient light. ii) Solution of compound **6** in toluene with UV‐lamp excitation (*λ*≈366 nm). *λ*_abs,max_: wavelength at the maximum of absorbance, *λ*_em,max_: wavelength at the maximum emission intensity, Φ_F_: fluorescence quantum yield.

**Figure 4:**
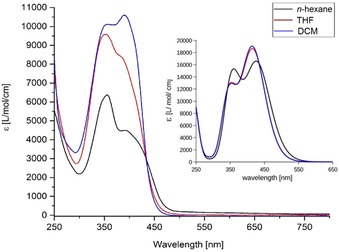
Experimental absorption spectra of **6**. Insert: Calculated absorption spectra of **6** using empirically corrected CAM‐B3LYP/cc‐pVDZ TD‐DFT excitation energies at PBE0/def2‐TZVP structures and a Gaussian broadening of FWHM=0.25 eV.

Given that HOMO–LUMO transitions often characterize or even determine absorption and fluorescence properties, BODIIM dyes must be expected to deviate from the established BODIPY dyes. Time‐dependent (TD)‐DFT studies of compound **B** associate absorption and emission within the planar BODIPY core without any contribution of the tolyl moiety at the *meso*‐position. Given that the HOMO and the LUMO display significant spatial overlap, strong absorption and intense fluorescence are predicted by theory, in full agreement with the experiment (both ca. 530 nm). The emission is reported with high quantum yield at a low Stokes shift (424 cm^−1^) in toluene solution (Figure 3).^17^ In contrast, in the same solvent the absorption of BODIIM **6** is found at 360, 400 nm with an intense greenish fluorescence at 520 nm resulting in a remarkable Stokes shift of 5800 cm^−1^. This behavior can be traced back to the modified orbital situation in the BODIIM system **6**, in which the transition occurs from the HOMO located in the heterocycle part into LUMO and LUMO+1 located on the phenyl entity with preferred orthogonal orientation. Accordingly, TD‐DFT modeling of the optical absorption spectra reveals two moderately intense close‐lying transitions located in the near‐UV region, which both carry heterocycle→arene charge‐transfer character. Inclusion of medium effects within the polarizable continuum model (PCM) accounts for the solvent‐dependent relative intensities (see insert Figure 4). In view of this aspect the photophysics of BODIIM **6** is clearly completely different from BODIPY **B** and BODIPY dyes in general because the aryl entity is essential by serving as the accepting unit in the absorption process. Although aryl entities in the *meso*‐position of BODIPY dyes are also known to give fluorescence‐diminished compounds at, however, only somewhat increased Stokes shift, the photophysical mechanism is described as an interaction of the aryl based HOMO or LUMO with the BODIPY core‐centered S_1_ excited state leading to accepting or donating photoelectron transfer processes (PeT) with the arene unit, that is, a‐PeT or d‐PeT mechanisms.^18^

**Figure 5:**
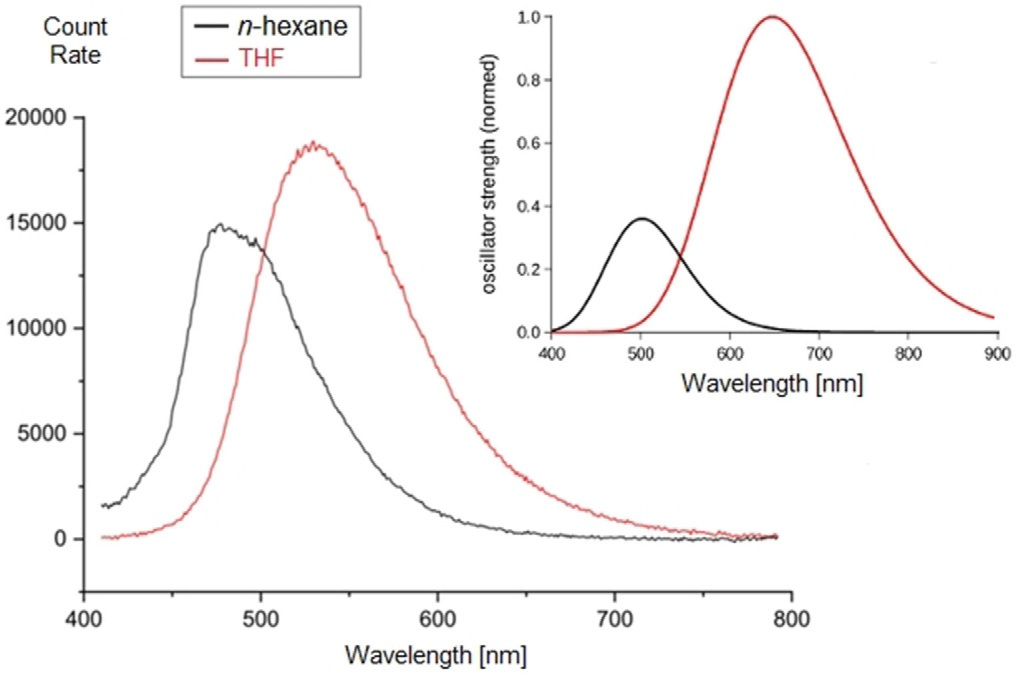
Experimental fluorescence spectra of compound **6**. Insert: Calculated emission spectra of **6** at wB97XD/def2‐TZVP TD‐DFT level of theory. Oscillatory strengths were normalized to 1.0 at *λ*_max_ in THF.

The experimental fluorescence spectra of **6** show single bands at 480 nm (*n*‐hexane) or 530 nm (THF), which are in excellent and good agreement with the calculated spectra, and give rise to significant Stokes shifts of >4,170 cm^−1^ in all studied solvents (Figure 5). The Stokes shift tends to increase with solvent polarity: *n*‐hexane (4170 cm^−1^)<toluene (5800 cm^−1^)<THF (6100 cm^−1^), corroborating the charge‐transfer (CT)‐like nature of the emissive excited state which was derived from TD‐DFT calculations. Accordingly, a further diminished Stokes shift of only 2000 cm^−1^ is predicted to prevail for gas‐phase conditions. A Stokes shift of such large extent again points out distinctive qualitative differences between the BODIIM and BODIPY chromophores. Given that the latter chromophore undergoes only mild structural evolution upon excitation, energies of excitation and emission are close to degenerate.

Accordingly, significant structural evolution is active in **6** which is revealed through inspection of the metrics of the optimized excited‐state structure of **6** (see Table S1, Supporting Information). In addition to a heterocycle‐borne C−N bond contraction and 1,4‐quinoidal distortion in the arene that are natural reporters of the charge shift upon excitation, the CT‐excited state of **6** undergoes a substantial contraction of the C−C single bond between the 14 π‐backbone and the arene; the difference in bond lengths between ground state (GS) and exited state (ES) amounts to less than 4 pm. We believe that this unique feature of a large Stokes shift in **6** will be attractive to the photophysical community, although a broader investigation of the photostability remains to be addressed. Compound **6** was found to suffer from photodecomposition in solvents of high polarity. Although in CH_2_Cl_2_ reproducible measurements were prevented by a slight extent of photodecomposition, the solvents *n*‐hexane and THF gave reproducible results.

**Scheme 3:**
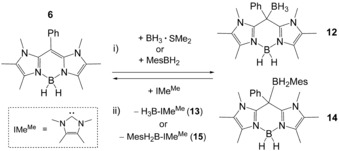
Key conditions and reagents. i) 1.3 equiv. BH_3_⋅SMe_2_ or MesBH_2_, toluene, rt, 30 min, 75 % (**12**) or 70 % (**14**). ii) 1 equiv. NHC, C_6_D_6_, rt, 5 % (**12**) or quantitative (**14**). Mes=2,4,6‐Me_3_C_6_H_2_.

In addition to the unique aspects of photophysics the HOMO in BODIIM **6** can be used as a representative map of the LUMO in BODIPY dyes. In particular, the strong p_z_‐contribution (24.6 %) of the *meso*‐carbon atom in the HOMO of compound **6** suggests a significant contribution to the LUMO in BODIPY **B** (23.1 %), whereas the HOMO of **B** essentially lacks the *meso*‐carbon contribution (0.0 %). This qualitative difference suggests the reactivity of BODIIM **6** as a carbon nucleophile at this distinct position; a behavior unparalleled by BODIPY dyes. Indeed, the addition of BH_3_ (introduced in the form of the adduct BH_3_⋅SMe_2_) as a representative Lewis acid afforded borane adduct **12** with attack at the *meso*‐position, which demonstrates the reactivity of **6** as a carbon nucleophile (Scheme 3). The reversibility of the adduct formation was probed with strong Lewis bases. Although phosphines, such as PPh_3_ and PMe_3_, proved to be inefficient, the N‐heterocyclic carbene (NHC) IMe^Me^ (1,3,4,5‐tetramethyl‐imidazol‐2‐ylidene) was found to produce **6** from **12** in approximately 5 % NMR spectroscopic yield. The poor reversibility was attributed to the strong donating ability of **6** at the *meso*‐carbon atom and can be estimated to be comparable to N‐heterocyclic carbenes. In view of a better reversibility for the donor–acceptor reaction, we reasoned that a bulkier borane would be bound weaker to the *meso*‐carbon atom in **6**. This hypothesis was studied with the sterically congested aryl borane MesBH_2_ (Mes=2,4,6‐Me_3_C_6_H_2_), the reaction of which with compound **6** gave the borane adduct **14**. In this case the treatment of **14** with IMe^Me^ led to a reversible, quantitative formation of **6**.

**Figure 6:**
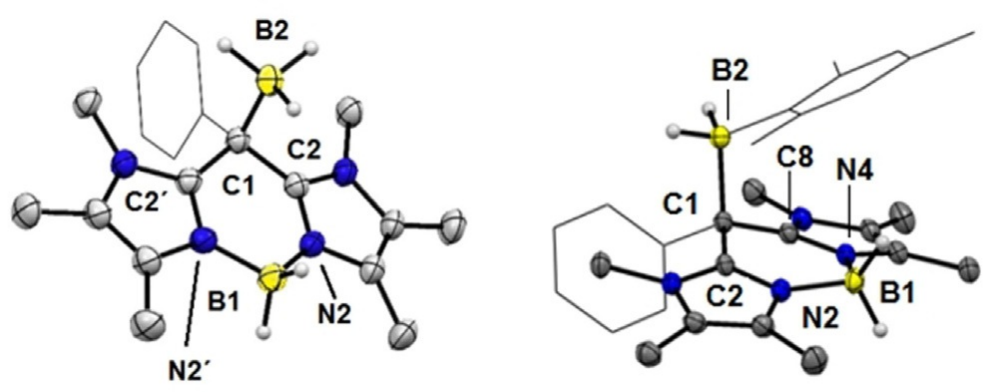
Molecular structure of compounds **12** (left) and **14** (right). The phenyl and mesityl entity are represented in the wireframe model. Thermal ellipsoids are drawn at the 50 % probability level. Carbon bound hydrogen atoms are omitted. The illustrated molecule for **12** is located on a crystallographic mirror plane containing B1, C1, B2 and the phenyl entity. Selected bond lengths [Å] and bond angles [°] for **12**: B1−N2 1.5443(16), C1−C2 1.4975(14), C2−N2 1.3330(15), C1−B2 1.679(2), C2‐C1‐C2' 108.16(13), C2‐C1‐B2 106.34(9), N2‐B1‐N2' 105.03(14); for **14**: B1−N2 1.5519(17), B1−N4 1.5543(18), C1−B2 1.734(2), C2−N2 1.3368(16), C8−N4 1.3340(16), C1−C2 1.5033(18), C1−C8 1.4989(17), N2‐B1‐N4 104.24(10), C8‐C1‐C2 107.40(10).

**Figure 7:**
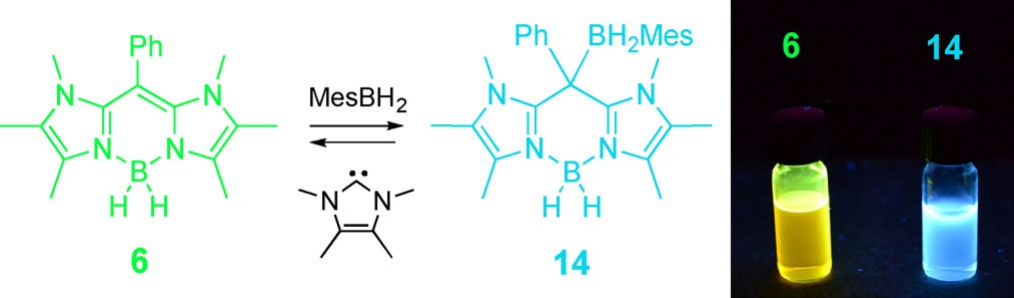
Upon irradiation (UV‐lamp, *λ*≈366 nm) NMR‐solutions of compound **6** and **14** (0.1 m in C_6_D_6_) show intense green fluorescence (**6**) and weak blue fluorescence (**14**).

Although borane adducts **12** and **14** were prepared on a synthetic scale to afford the isolated and analytically pure compounds, the reverse process was studied by ^1^H and ^11^B{^1^H} NMR monitoring. Thus, the isolated borane adducts **12** and **14** were reacted with a stoichiometric amount of IMe^Me^ in C_6_D_6_ on typical NMR scale and retained compound **6** and the IMe^Me^ borane adducts **13** and **15**, respectively. The latter were also prepared in analytically pure form by independent reactions of the carbene IMe^Me^ with the boranes BH_3_⋅SMe_2_ or MesBH_2_. The comparison of ^1^H and ^11^B{^1^H} NMR spectra of reaction mixtures with isolated compounds allowed for the unambiguous identification of **13** and **15** as side products in the reverse process, for an example see the Supporting Information. Compounds **12** and **14** were also structurally authenticated by X‐ray crystallography (Figure 6). The molecular structures clearly reveal a tetrahedral environment for the *meso*‐carbon atom C1 upon coordination of the respective borane. In particular, the C1−B2 bond in **12** (1.679(2) Å) is found to be shorter than in **14** (1.734(2) Å) indicating a weaker bond in latter case, which is in accordance with the quantitative decomplexation of the MesBH_2_ entity in **14** upon addition of the IMe^Me^. The reversible borane complexation is also interesting from the standpoint of switching the optical properties in compound **6**. The electrophilic attack of the borane takes place at the *meso*‐carbon atom with involvement of the HOMO. Given that this orbital is directly involved in the absorption process, the borane complexation should have a strong impact on the absorption and fluorescence properties. Indeed, the reaction of yellowish compound **6** with BH_3_ or MesBH_2_ quenches both the absorption (360–400 nm) and the greenish fluorescence (480–530 nm). The colorless borane adducts **12** and **14** were found to be weakly absorbing between 300–400 nm and a weak blue fluorescence was observed upon excitation with a laboratory UV‐lamp (*λ*≈366 nm; Figure 7). Due to its weak nature the fluorescence in **14** was not further investigated. Although dilute solutions of **6** (10^−5^ m in C_6_D_6_) still showed strong fluorescence, the blue fluorescence of **14** was macroscopically not visible at the same concentration. We wish to point out that the reactivity of BODIIM **6** as a carbon nucleophile to bind and release Lewis acids (here shown with boranes) can be monitored by the concomitant switch of the fluorescence. This behavior is in pronounced contrast to BODIPY systems, for which such reactivity is completely unknown, and we suggest applications of our system as a reversible fluorescence switch in the presence of Lewis acids and bases.

## Conclusions

(aza‐)BODIPY dyes (boron dipyrromethene dyes) are well‐established fluorophores due to their excellent quantum yields, stability and diversity. Although the control of the optical properties in (aza‐) BODIPY dyes by peripheral functional groups is well studied, we herein presented a novel approach to modify the 12 π‐electron system of the BODIPY scaffold. We presented a first prototype of a fluorescent dye termed BODIIM (**6**, boron diimidazolylmethene), which was obtained by the formal replacement of CH groups in the β‐position by nitrogen atoms in the organic π‐system of a BODIPY. The resulting 14 π‐electron system in the BODIIM dye can be considered as an extension of the 12 π‐electron system in a BODIPY dye. DFT‐Calculations confirm the [12+2] π‐electron relationship between BODIPY and BODIIM cores and reveal a strong shape correlation between LUMO in the BODIPY and the HOMO of the BODIIM. The BODIIM prototype **6** proved to be less stable than BODIPY dyes: Solid samples were found to be bench‐stable for a period of 2 weeks but solutions of **6** in [D_8_]THF exposed to air showed decomposition to an extent of 1–2 % within 24 h as assessed by NMR monitoring. Although reproducible fluorescence spectra were obtained in *n*‐hexane and THF solutions of **6** in CH_2_Cl_2_ showed signs of photo‐decomposition. However, in view of the fact that compound **6** is the first prototype of BODIIM these problems can be addressed by careful structural design in future work. Remarkably, the BODIIM prototype compound **6** offers several features which are unprecedented for the BODIPY dye class: (i) Even though compound **6** was obtained with a BH_2_ rigidifying entity, this structural motive was suggested but not proven for the BODIPY dye class.^16^ (ii) The modification of the π‐system leads to a dramatic shift of the optical properties, of which the fluorescent emission is most noteworthy and occurs at much higher Stokes shift, that is, ≈500 cm^−1^ in BODIPY versus at least 4170 cm^−1^ in BODIIM systems in all solvents investigated. (iii) Nucleophilic reactivity was found at the *meso*‐carbon atom in the formation of stable borane adducts with BH_3_ (**12**) and MesBH_2_ (**14**) displaying a significant shift of the fluorescent emission. Additionally, the reverse decomplexation of the borane adducts was demonstrated in reactions with a representative N‐heterocyclic carbene to retain the strongly fluorescent BODIIM **6**. This reactivity is in contrast to BODIPY systems, which lack such nucleophilic behavior. We suggest our system as a fully reversible fluorescent switch to probe Lewis acids and bases, in particular for systems of academic interest, for example, frustrated Lewis pairs (FLP). The fluorescence lifetime is not reported but will be published within a library of modified derivatives to allow for a consistent comparison. In addition to the improvement of the stability of the prototype compound **6**, future work also will focus on the influence of the torsion angle of the phenyl moiety. In preliminary computational studies this torsion angle was systematically tilted from the preferred orthogonal orientation and a strong increase of the transition moments for both absorption and fluorescence emission with a concomitant hypsochromic shift was found (see Table S2, S3 and Figure S48, Supporting Information).

## Conflict of interest

The authors declare no conflict of interest.

## Supporting information

As a service to our authors and readers, this journal provides supporting information supplied by the authors. Such materials are peer reviewed and may be re‐organized for online delivery, but are not copy‐edited or typeset. Technical support issues arising from supporting information (other than missing files) should be addressed to the authors.

Supplementary

## References

[1] T. E. Wood , A. Thompson , Chem. Rev. 2007, 107, 1831–1861. 17430001 10.1021/cr050052c

[2] For chemical transformations:

[2a] A. Loudet , K. Burgess , Chem. Rev. 2007, 107, 4891–4932; 17924696 10.1021/cr078381n

[2b] G. Ulrich , R. Ziessel , A. Harriman , Angew. Chem. Int. Ed. 2008, 47, 1184–1201; 10.1002/anie.200702070 18092309

[2c] N. Boens , B. Verbelen , W. Dehaen , Eur. J. Org. Chem. 2015, 6577–6595;

[2d] E. Bodio , C. Goze , Dyes Pigm. 2019, 160, 700–710.

[3a] A. V. Solomonov , Y. S. Marfin , E. V. Rumyantsev , Dyes Pigm. 2019, 162, 517–542;

[3b] S. Kolemen , E. U. Akkaya , Coord. Chem. Rev. 2018, 354, 121–134;

[3c] N. Boens , V. Leen , W. Dehaen , Chem. Soc. Rev. 2012, 41, 1130–1172. 21796324 10.1039/c1cs15132k

[4] S. C. Lee , J. Heo , H. C. Woo , J.-A. Lee , Y. H. Seo , C.-L. Lee , S. Kim , O.-P. Kwon , Chem. Eur. J. 2018, 24, 13706–13718. 29700889 10.1002/chem.201801389

[5a] D. Ho , R. Ozdemir , H. Kim , T. Earmme , H. Usta , C. Kim , ChemPlusChem 2019, 84, 18–37; 31950740 10.1002/cplu.201800543

[5b] H. Klfout , A. Stewart , M. Elkhalifa , H. He , ACS Appl. Mater. Interfaces 2017, 9, 39873–39889; 29072443 10.1021/acsami.7b07688

[5c] S. A. Baudron , CrystEngComm 2016, 18, 4671–4680.

[6a] T. Zhang , C. Ma , T. Sun , Z. Xie , Coord. Chem. Rev. 2019, 390, 76–85;

[6b] Y. S. Marfin , A. V. Solomonov , A. S. Timin , E. V. Rumyantsev , Curr. Med. Chem. 2017, 24, 2745–2772. 28571557 10.2174/0929867324666170601092327

[7a] W. Sun , X. Zhao , J. Fan , J. Du , X. Peng , Small 2019, 15, 1804927; 10.1002/smll.201804927 30785670

[7b] A. M. Durantini , D. A. Heredia , J. E. Durantini , E. N. Durantini , Eur. J. Med. Chem. 2018, 144, 651–661. 29289888 10.1016/j.ejmech.2017.12.068

[8a] J. Zhao , K. Chen , Y. Hou , Y. Che , L. Liu , D. Jia , Org. Biomol. Chem. 2018, 16, 3692–3701. 29693683 10.1039/c8ob00421h

[9a] A. Schmitt , B. Hinkeldey , M. Wild , G. Jung , J. Fluoresc. 2009, 19, 755–759; 19067126 10.1007/s10895-008-0446-7

[9b] K. Tram , H. Yan , H. A. Jenkins , S. Vassiliev , D. Bruce , Dyes Pigm. 2009, 82, 392–395;

[9c] I. J. Arroyo , R. Hu , G. Merino , B. Z. Tang , E. Peña-Cabrera , J. Org. Chem. 2009, 74, 5719–5722. 19572588 10.1021/jo901014w

[10] A. Treibs , F. H. Kreuzer , Justus Liebigs Ann. Chem. 1968, 718, 208–223. 10.1002/jlac.19687180118 5704489

[11a] For the alkyl-substituted BODIPY core see: E. V. de Wael , J. A. Pardoen , J. A. v. Koeveringe , J. Lugtenburg , Recl. Tra V. Chim. Pays-Bas 1977, 96, 306–309;

[11b] R. Bandichhor , C. Thivierge , N. S. P. Bhuvanesh , K. Burgess , Acta Crystallogr. Sect. E 2006, 62, o4310–o4311.

[12a] Examples include alkyl-, aryl- and alkynyl groups: H. L. Kee , C. Kirmaier , L. Yu , P. Thamyongkit , W. J. Youngblood , M. E. Calder , L. Ramos , B. C. Noll , D. F. Bocian , W. R. Scheidt , R. R. Birge , J. S. Lindsey , D. Holten , J. Phys. Chem. B 2005, 109, 20433–20443; 16853644 10.1021/jp0525078 PMC1513631

[12b] C. Goze , G. Ulrich , L. J. Mallon , B. D. Allen , A. Harriman , R. Ziessel , J. Am. Chem. Soc. 2006, 128, 10231–10239; 16881653 10.1021/ja062405a

[12c] G. Ulrich , C. Goze , M. Guardigli , A. Roda , R. Ziessel , Angew. Chem. Int. Ed. 2005, 44, 3694–3698; 10.1002/anie.200500808 15915531

[12d] R. Ziessel , C. Goze , G. Ulrich , Synthesis 2007, 6, 936–949;

[12e] R. Ziessel , G. Ulrich , A. Harriman , New J. Chem. 2007, 31, 496–501.

[13a] J. Killoran , L. Allen , J. Gallagher , W. Gallagher , D. O'Shea , Chem. Commun. 2002, 1862–1863; 10.1039/b204317c 12271646

[13b] A. Gorman , J. Killoran , C. O'Shea , T. Kenna , W. M. Gallagher , D. F. O'Shea , J. Am. Chem. Soc. 2004, 126, 10619–10631; 15327320 10.1021/ja047649e

[13c] S. O. McDonnell , D. F. O'Shea , Org. Lett. 2006, 8, 3493–3496; 16869643 10.1021/ol061171x

[13d] X.-D. Jiang , S. Li , J. Guan , T. Fang , L. Tao , X. Liu , L.-J. Xiao , Curr. Org. Chem. 2016, 20, 1736–1744;

[13e] Y. Ni , J. Wu , Org. Biomol. Chem. 2014, 12, 3774–3791. 24781214 10.1039/c3ob42554a

[14] T. W. Ross , G. Sathyamoorthi , J. H. Boyer , Heteroat. Chem. 1993, 4, 609–612.

[15] Y. Zhou , Y. Gong , Eur. J. Org. Chem. 2011, 6092–6099.

[16] C. Bonnier , W. E. Piers , M. Parvez , Organometallics 2011, 30, 1067–1072.

[17] M. Bröring , R. Krüger , S. Link , C. Kleeberg , S. Köhler , X. Xie , B. Ventura , L. Flamigni , Chem. Eur. J. 2008, 14, 2976–2983. 18306269 10.1002/chem.200701912

[18a] A. P. de Silva , H. G. N. Gunaratne , T. Gunnlaugsson , A. J. M. Huxley , C. P. McCoy , J. T. Rademacher , T. E. Rice , Chem. Rev. 1997, 97, 1515–1566; 11851458 10.1021/cr960386p

[18b] H. Sunahara , Y. Urano , H. Kojima , T. Nagano , J. Am. Chem. Soc. 2007, 129, 5597–5604; see also Ref. [2a]. 17425310 10.1021/ja068551y

