# Supplementary material for: Two π‐Electrons Make the Difference: From BODIPY to BODIIM Switchable Fluorescent Dyes
Source: Chemistry. 2020 Jan 9;26(6):1422–8. doi: 10.1002/chem.201905344 (PMC7027818; doi:10.1002/chem.201905344)
Supplement: Supplementary file 1 — Supplementary [file CHEM-26-1422-s001.pdf]

CHEMISTRY
A European Journal

Supporting Information

Two π-Electrons Make the Difference: From BODIPY to BODIIM Switchable Fluorescent Dyes

Hadi Dolati,[a] Lisa C. Haufe,[a] Lars Denker,[a] Andreas Lorbach,[b] Robin Grotjahn,[c] Gerald Hörner,*[c, d] and René Frank*[a]

chem_201905344_sm_miscellaneous_information.pdf

1. General Information

All manipulations were performed under dry argon atmosphere using Schlenk techniques or in a glove box (M. Braun 200B model) unless stated otherwise. Solvents were purified and dried using a Solvent Purification System (M. Braun) and stored over molecular sieves (3−4 Å). All commercially available compounds (TCI, abcr, deutero, Sigma Aldrich) were used without further purification. Deuterated solvents were dried over sodium (C_6D_6, THF-D_8) or CaH_2 (CD_2Cl_2, $CDCl_3$), distilled under argon and stored over molecular sieves (3−4 Å).The compounds **7**,[1] **13**,[2] IMe^{Me} [3] and $MesBH_2$ [4] were prepared according to literature methods.

NMR spectra were recorded on Bruker Avance II-300, Avance III-HD, Avance III-400 and AVII-600 spectrometer. The chemical shifts (δ) are reported in parts per million (ppm). The residual solvent peak (C_6HD_5, δ = 7.16 ppm, THF-HD_7, δ = 1.72, 3.58 ppm, $CHCl_3$, δ = 7.26 ppm, $CHDCl_2$, δ = 5.32 ppm) is used for the referencing of the ^{1}H-NMR spectra. The ^{13}C spectra are internally calibrated by using the ^{13}C resonances of the solvent peaks (C_6D_6, δ = 128.06 ppm, THF-D_8, δ = 25.31, 67.21 ppm, $CDCl_3$, δ = 77.16 ppm, CD_2Cl_2, δ = 53.84 ppm). For ^{11}B-NMR spectra an external calibration with $BF_3 \cdot Et_2O$ was used. Coupling constants are stated in Hertz (Hz), multiplicities are defined as br (broad), s (singlet), d (doublet), t (triplet), sept (septet) or m (multiplet). If necessary, 2D-NMR experiments (H,H-COSY, H,C-HSQC, H,C-HMBC) were used to aid the assignment of the signals.

IR spectra were recorded on a Bruker Vertex 70 with the ATR technique.

Mass spectra were recorded on a Finnigan MAT 8400-MSS I instrument (for electro spray ionization, ESI) or on a Finnigan MAT 4515 instrument (electron impact mode, EI) and are reported as the m/z ratio (in Da).

Elemental analyses were accomplished by combustion and gas chromatographic analysis using a VarioMICRO Tube and HW detection. Values are reported in weight-%.

All optical measurements were carried out in quartz glass cuvettes.

UV-VIS absorption spectra were recorded on a Varian Cary 50 device in the solvents *n*-hexane, THF and DCM of spectroscopic purity and were dried over sodium (n-hexane, THF) or CaH_2 (DCM).

Photoluminescence measurements on solutions of **6** in degassed and dried *n*-hexane and THF were carried out on a *PicoQuant FluoTime 300* spectrometer. In both solvents, excitation at 360 nm and 400 nm led to superimposable emission spectra. Excitation spectra with detection wavelengths of 480 nm (*n*-hexane) and 530 nm (THF) displayed the same band structures as the corresponding absorption spectra.

Absolute photoluminescence quantum yields were determined on a *Hamamatsu C9920-02* spectrometer using an integrating sphere. Freshly prepared solutions of **6** in degassed and dried toluene (spectroscopic grade) showed absorbance maxima of $\lambda_{abs,max}$ = 360, 400 nm and maximum emission intensity at $\lambda_{em,max}$ = 520 nm. Toluene solutions with an absorbance of A_{400} = 0.08 showed a considerable decrease in quantum yield (initially 26%) after repeated measurements within 10 min. To reduce the relative amount of decomposition products, samples with higher concentrations were also

investigated (A_{400} = 0.9: QY = 27%; A_{400} > 3: QY = 22%). Excitation at 360 nm did not lead to a change in quantum yield.

2. Synthetic Procedures and Analytical Data

2.1. Compound **8**.

Imidazole **7** (5.19 g, 47.11 mmol, 1.00 eq.) was dissolved in tetrahydrofuran (50 mL) and cooled to −30 °C. *n*-BuLi (29.5 mL, 47.1 mmol, 1.00 eq., 1.6 M in hexanes) was added dropwise to the colorless solution. Upon addition of *n*-BuLi a yellowish precipitate formed, which dissolved as soon as the amount of *n*-BuLi exceeded ca. 24 mL. After complete addition, the clear yellow solution was stirred for 30 min with warming to 0 °C. Benzoyl chloride [PhC(O)Cl, 2.46 mL, 21.2 mmol, 0.45 eq.] was added dropwise at 0 °C. The dark red solution was stirred for 1 h with warming to room temperature and then quenched with water (10 mL). The solvent was removed under reduced pressure, and water (80 mL) was added. The mixture was extracted with chloroform (3 × 80 mL) and the combined organic phases were dried over $MgSO_4$. The solvent was removed under reduced pressure and dried in high vacuum. The orange solid was recrystallized by diffusion of diethyl ether vapor into a saturated solution of the crude product in chloroform. Compound **8** was isolated as an off-white solid (4.47 g, 13.78 mmol, 65 %). Analytically pure samples were obtained by a second recrystallization. Crystals suitable for X-ray crystallography were harvested by diffusion of *n*-pentane into a solution of compound **8** in chloroform.

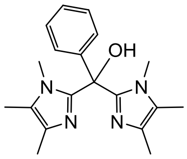

Chemical Formula: $C_{19}H_{24}N_4O$
Exact Mass: 324,20
Molecular Weight: 324,43
m/z: 324.20 (100.0%), 325.20 (20.5%), 326.20 (2.0%), 325.19 (1.5%)
Elemental Analysis: C, 70.34; H, 7.46; N, 17.27; O, 4.93

^{1}H-NMR ($CDCl_3$, 500 MHz, 293 K): δ 2.06 (6 H, s, CMe), 2.10 (6 H, s, CMe), 3.20 (6 H, s, NMe), 6.56 (1 H, s, OH), 7.07−7.09 (2 H, m, aryl-CH), 7.24−7.29 (3 H, m, aryl-CH).

^{13}C{^{1}H}-NMR ($CDCl_3$, 125.8 MHz, 293 K): δ 9.1 (CMe), 12.9 (CMe), 32.0 (NMe), 74.7 (C−OH), 125.0 (C_q), 127.6 (aryl CH), 127.9 (aryl CH), 128.2 (aryl CH), 130.5 (C_q), 143.0 (NCMe), 146.2 (NCMe).

IR: $\tilde{\upsilon}$ = 3196 (O−H), 3084 (aryl C−H), 2940 (alkyl C−H), 1600 (C=C), 1330, 1082 cm^{-1}.

MS (ESI): m/z = 324.96 $[M+H]^+$, 346.93 $[M+Na]^+$.

Elemental Analysis: Calculated: C 70.34, H 7.46, N 17.27. Found: C 70.13, H 7.05, N 17.34.

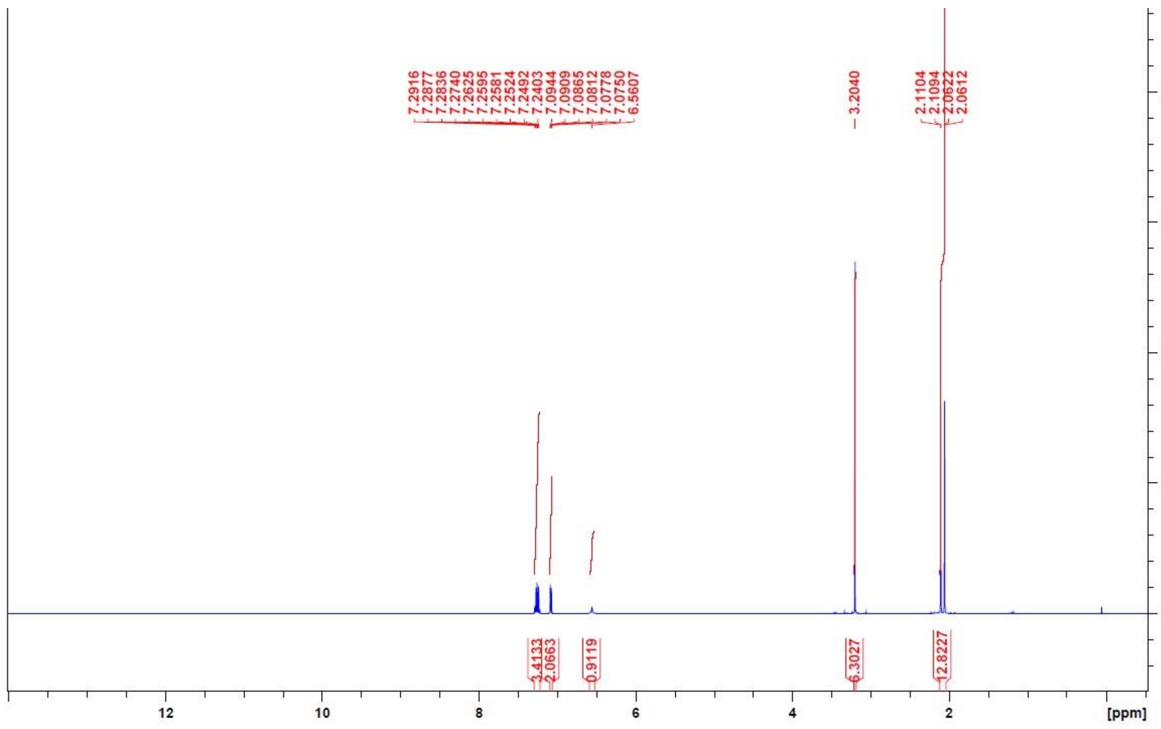

Figure S1. ^{1}H-NMR spectrum ($CDCl_3$, 500 MHz) of compound **8**

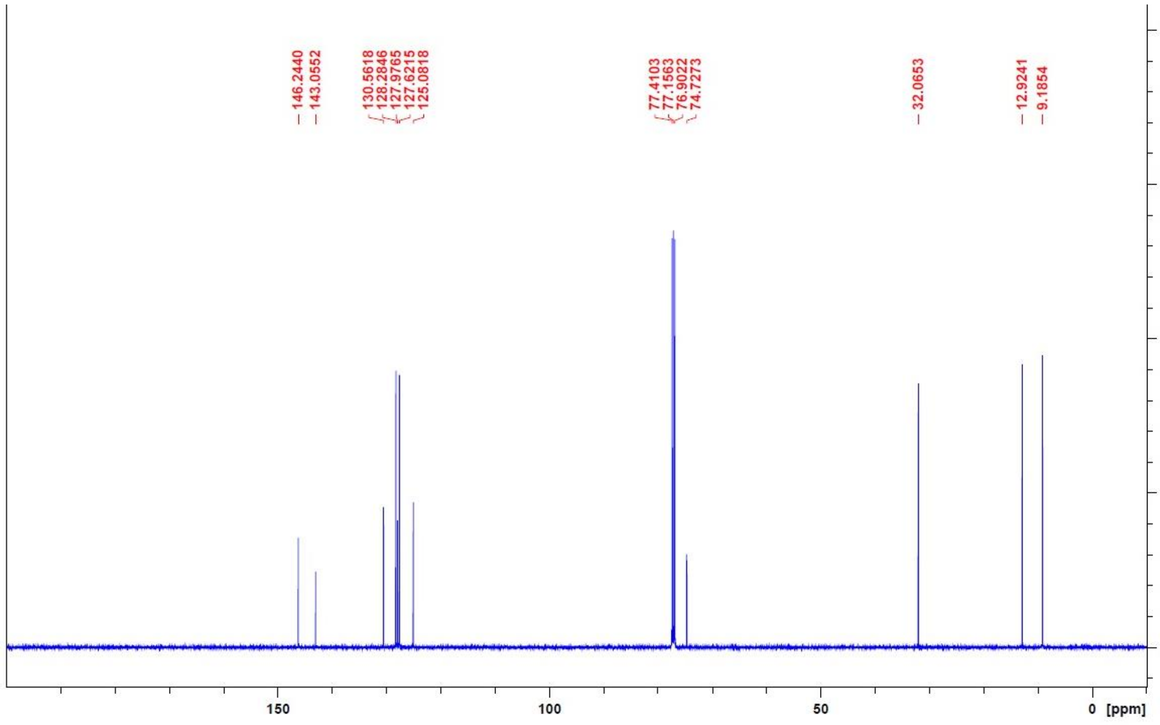

Figure S2. $^{13}C\{^{1}H\}$-NMR spectrum ($CDCl_3$, 125.8 MHz) of compound **8**

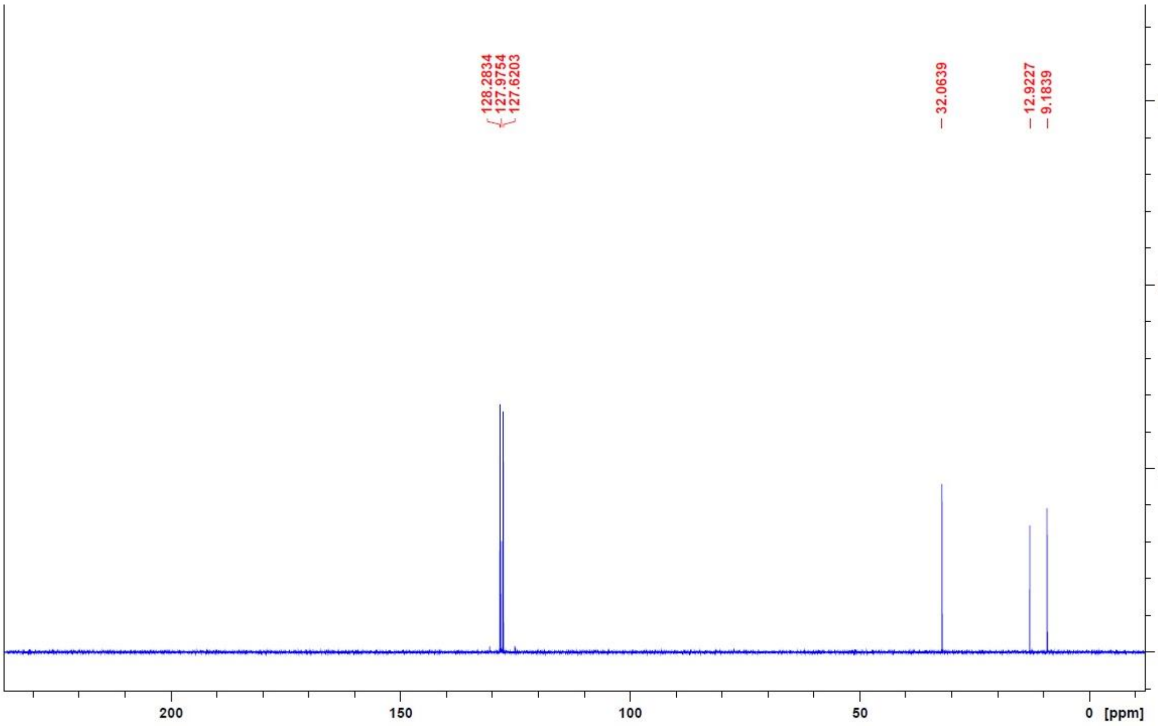

Figure S3. ^{13}C-DEPT NMR spectrum ($CDCl_3$, 125.8 MHz) of compound **8**

2.2. Compound **9**.

Imidazole **7** (5.00 g, 45.39 mmol, 1.00 eq.) was dissolved in dichloromethane (40 mL) and cooled to 0 °C. $BH_2Cl{\cdot}SMe_2$ (22.7 mL, 22.69 mmol, 0.50 eq., 1 M in dichloromethane) was added dropwise. The cooling bath was removed and the reaction was warmed to ambient temperature within 15 min. Tetrahydrofuran (10 mL) was added and the solution was stirred for 15 min. Hexanes (200 mL) was slowly added within 2 min with vigorous stirring, which afforded a colorless precipitate. The supernatant solution was removed by cannula-filtration. The precipitate was dried in high vacuum to give compound **9** as a colorless solid (5.79 g, 21.56 mmol, 95 %), which was sufficiently pure for further manipulation. Analytically pure samples were obtained by diffusion of *n*-pentane vapor into a solution of compound **9** in chloroform.

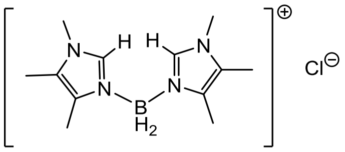

^{1}H-NMR (CD_2Cl_2, 400 MHz, 293 K): δ 2.12 (12 H, s, CMe), 2.5–4.0 (br, BH_2), 3.72 (6 H, s, NMe), 8.83 (2 H, s, CH).

^{13}C{^{1}H}-NMR (CD_2Cl_2, 100.6 MHz, 293 K): δ 8.3 (CMe), 9.8 (CMe), 33.2 (NMe), 126.2 (N*C*Me), 129.1 (N*C*Me), 138.5 (NCN).

^{11}B{^{1}H}-NMR (CD_2Cl_2, 96.2 MHz, 293 K): δ −11.5 (BH_2, $\omega_{1/2}$ = 105 Hz).

^{11}B-NMR (CD_2Cl_2, 96.2 MHz, 293 K): δ −11.4 (BH_2, t, $^{1}J_{BH}$ = 96 Hz).

IR: $\tilde{\upsilon}$ = 3108, 3015 (both aryl C−H), 2978 (alkyl C−H), 2424 (B−H), 2397 (B−H), 2360 (B−H), 1626 (C=C), 1548 cm^{-1}.

MS (ESI): m/z = 233.06 $[M–Cl]^{+}$.

Elemental Analysis: Calculated: C 53.66, H 8.26, N 20.68. Found: C 54.03, H 8.53, N, 20.53.

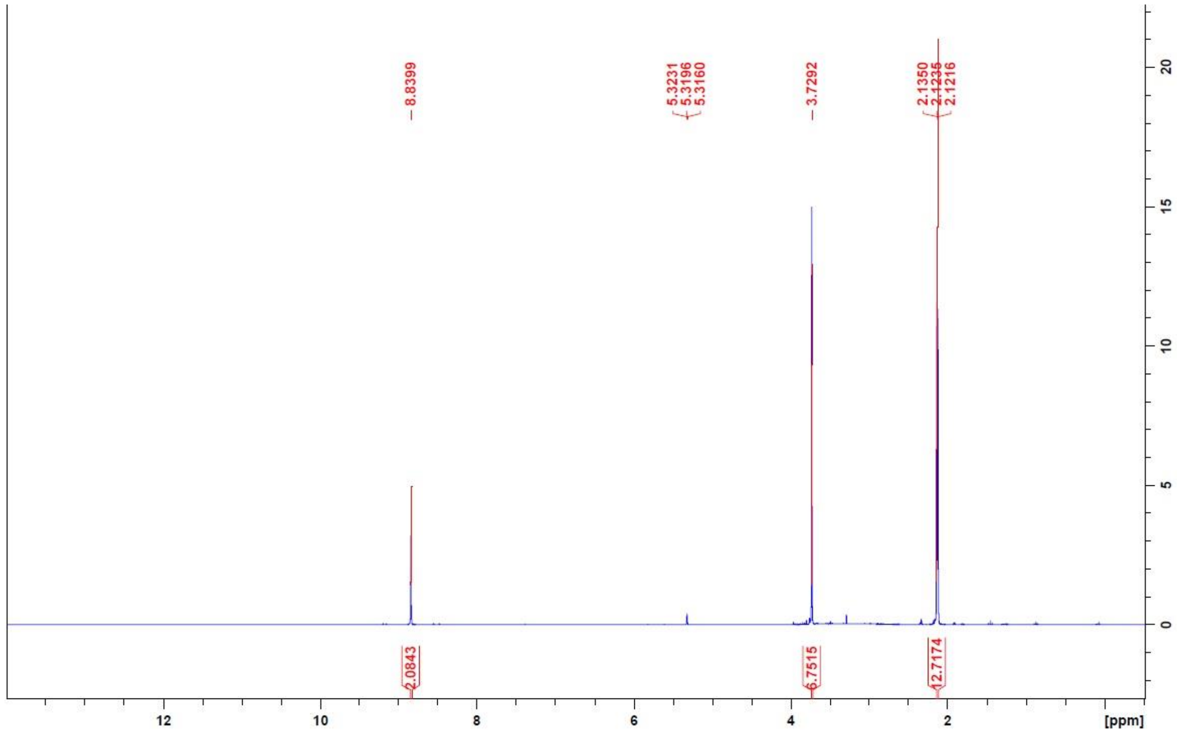

Figure S4. ^{1}H-NMR spectrum (CD_2Cl_2, 400 MHz) of compound **9**

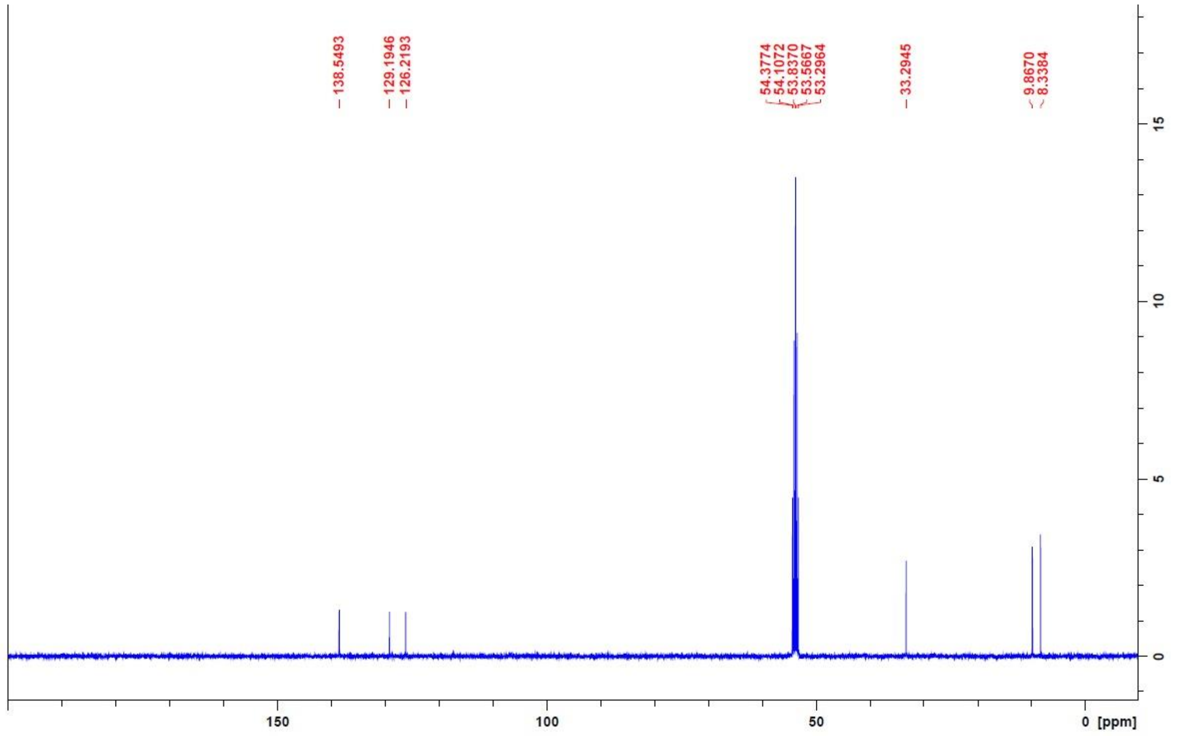

Figure S5. $^{13}C\{^{1}H\}$-NMR spectrum (CD_2Cl_2, 100.6 MHz) of compound **9**

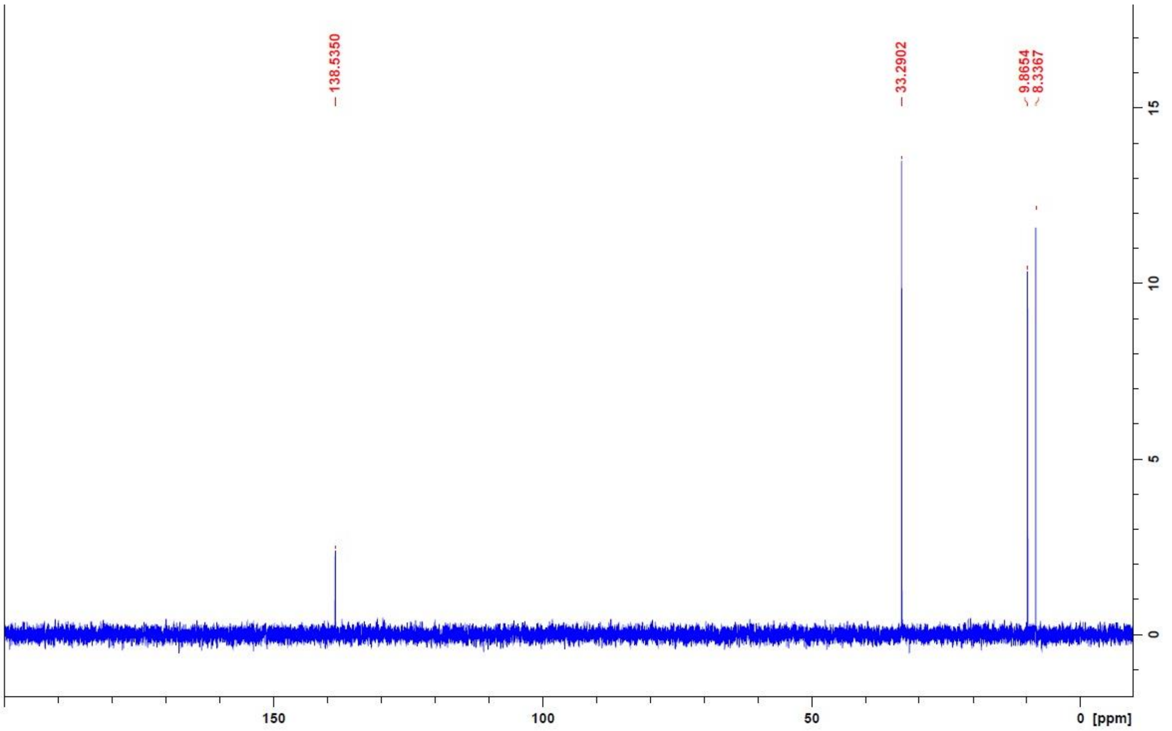

Figure S6. ^{13}C-DEPT-NMR spectrum (CD_2Cl_2, 100.6 MHz) of compound **9**

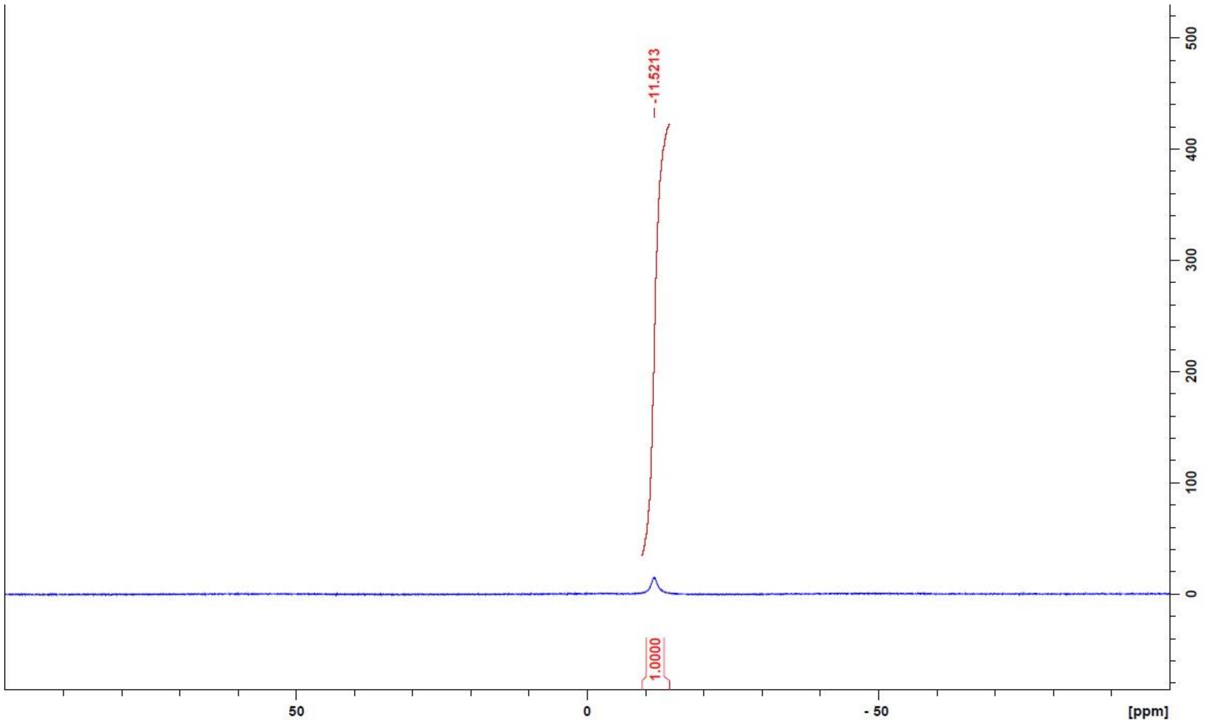

Figure S7. $^{11}B\{^1H\}$-NMR spectrum (CD_2Cl_2, 96.2 MHz) of compound **9**

2.3. Compound **10**.

Compound **9** (5.00 g, 18.62 mmol, 1.00 eq.) was suspended in tetrahydrofuran (100 mL), cooled to −78 °C by means of an acetone dry ice bath and stirred for 10 min. *n*-BuLi (23.85 mL, 38.16 mmol, 2.05 eq., 1.6 M in hexanes) was added dropwise with stirring. The reaction was covered with aluminum foil and slowly allowed to warm to ambient temperature overnight.

Annotation: Dry ice pellets of ca. 1 cm in diameter were employed. The volume of the cooling liquid (acetone) should be at least 0.5 L. The slow warming process was found to be important. Faster warming without insulation of the reaction-setup afforded samples of lower purity.

The solid dissolved completely and the solution turned yellowish. Methyl benzoate [PhC(O)OMe, 2.66 g, 19.55 mmol, 1.05 eq.] was added dropwise. The solution turned dark red and was stirred for 1 h at ambient temperature. The mixture was quenched with water (20 mL) and all volatiles were removed at reduced pressure. The crude mixture was dissolved a two-phase-system composed of chloroform (200 mL) and saturated brine (150 mL). The organic phase was dried over $MgSO_4$ and the solvent removed in high vacuum. Compound **10** was obtained as a pale-yellow solid (6.59 g, 17.69 mmol, 95 %), which was sufficiently pure for further manipulation. Analytically pure samples and crystals suitable for X-ray crystallography were obtained by diffusion of *n*-pentane vapor into a solution of compound **10** in chloroform.

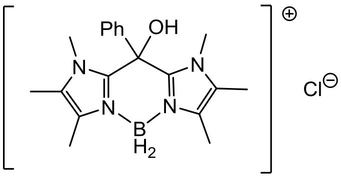

^{1}H-NMR (CD_2Cl_2, 400 MHz, 293 K): δ 2.10 (6 H, s, CMe), 2.25 (6 H, s, CMe), 3.0–4.0 (br, BH_2), 3.55 (6 H, s, NMe), 7.30−7.43 (5 H, m, aryl-CH), 9.88 (H, s, OH).

^{13}C{^{1}H}-NMR (CD_2Cl_2, 100.6 MHz, 293 K): δ 8.5 (CMe), 9.4 (CMe), 32.3 (NMe), 78.1 (C−OH), 125.6 (aryl CH), 127.2, 127.6, 129.0 (aryl CH), 129.2 (aryl CH), 139.4 (NCMe), 141.8 (N*C*Me).

^{11}B{^{1}H}-NMR (CD_2Cl_2, 96.2 MHz, 293 K): δ −11.2 (BH_2, $\omega_{1/2}$ = 238 Hz).

^{11}B{^{1}H}-NMR (CD_2Cl_2, 96.2 MHz, 293 K): δ −11.2 (BH_2, br, s).

IR: $\tilde{\upsilon}$ = 3080 (aryl C−H), 2950 (alkyl C−H), 2740 (O−H), 2390 (B−H), 2380 (B−H), 2338 (B−H), 1633 (C=C), 1536 cm^{-1}.

MS (ESI): m/z = 337.20 $[M–Cl]^{+}$.

Elemental Analysis: Calculated: C 61.23, H 7.03, N 15.03. Found: C 61.15, H 6.95, N 15.15.

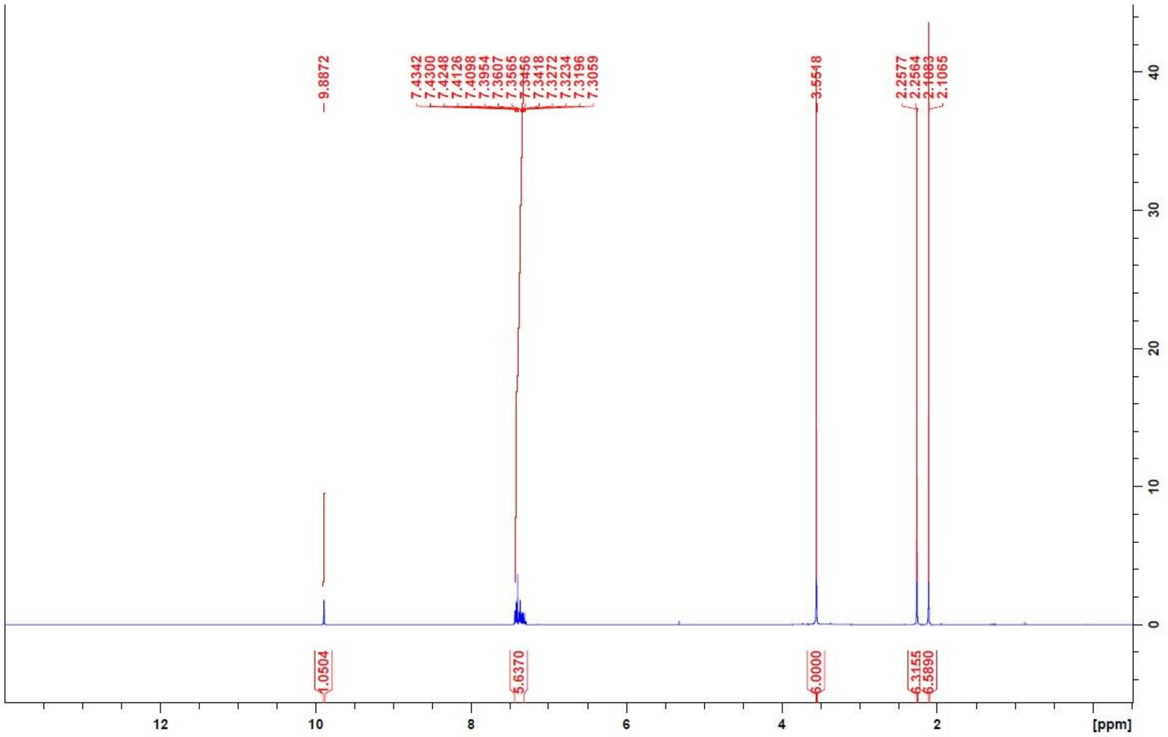

Figure S8. ^{1}H-NMR spectrum (CD_2Cl_2, 400 MHz) of compound **10**

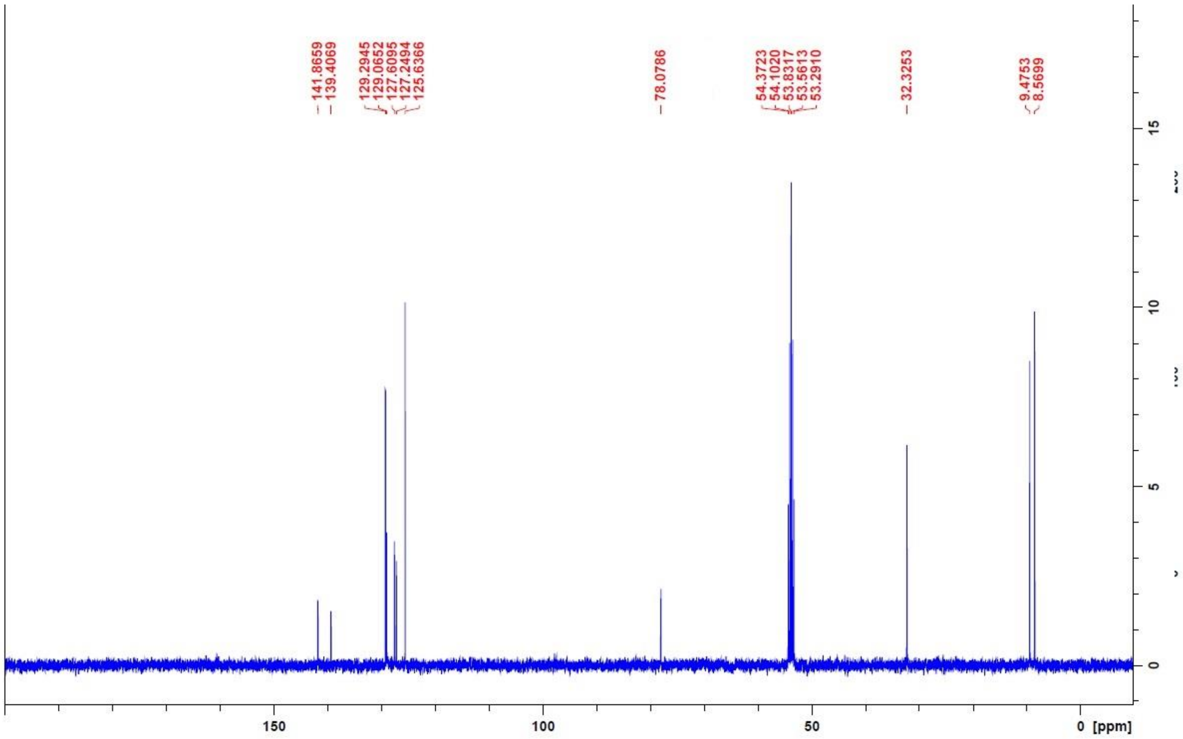

Figure S9. $^{13}C\{^1H\}$-NMR spectrum (CD_2Cl_2, 100.6 MHz) of compound **10**

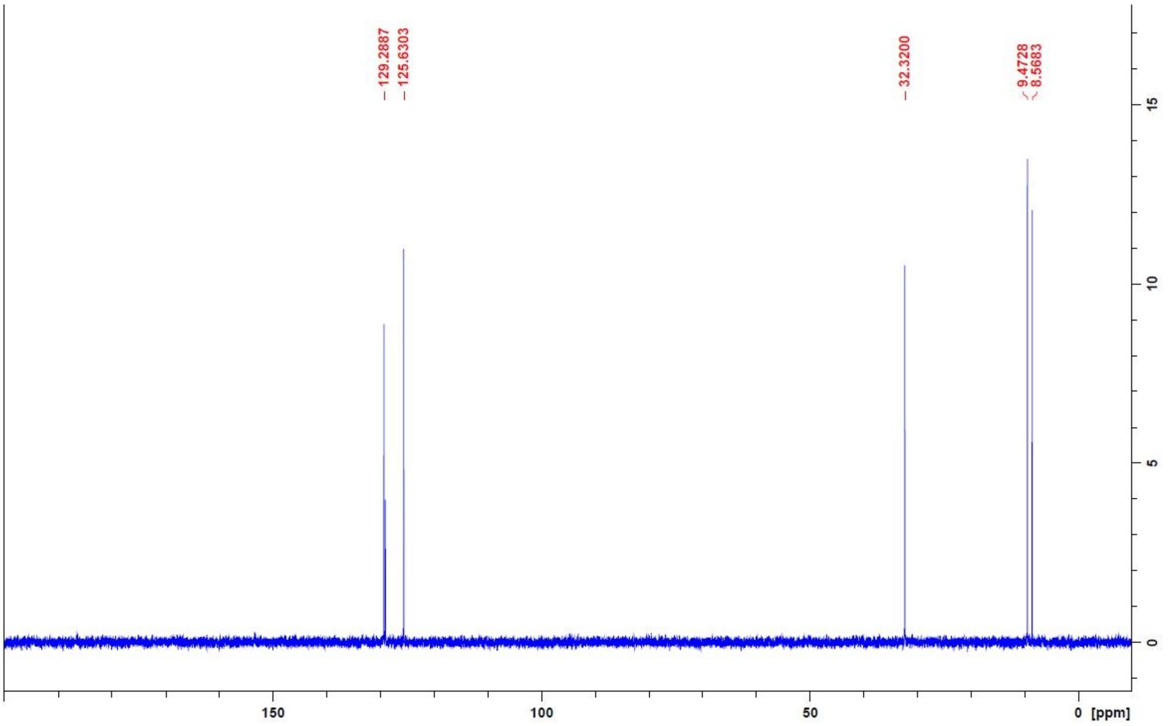

Figure S10. ^{13}C-DEPT-NMR spectrum (CD_2Cl_2, 100.6 MHz) of compound **10**

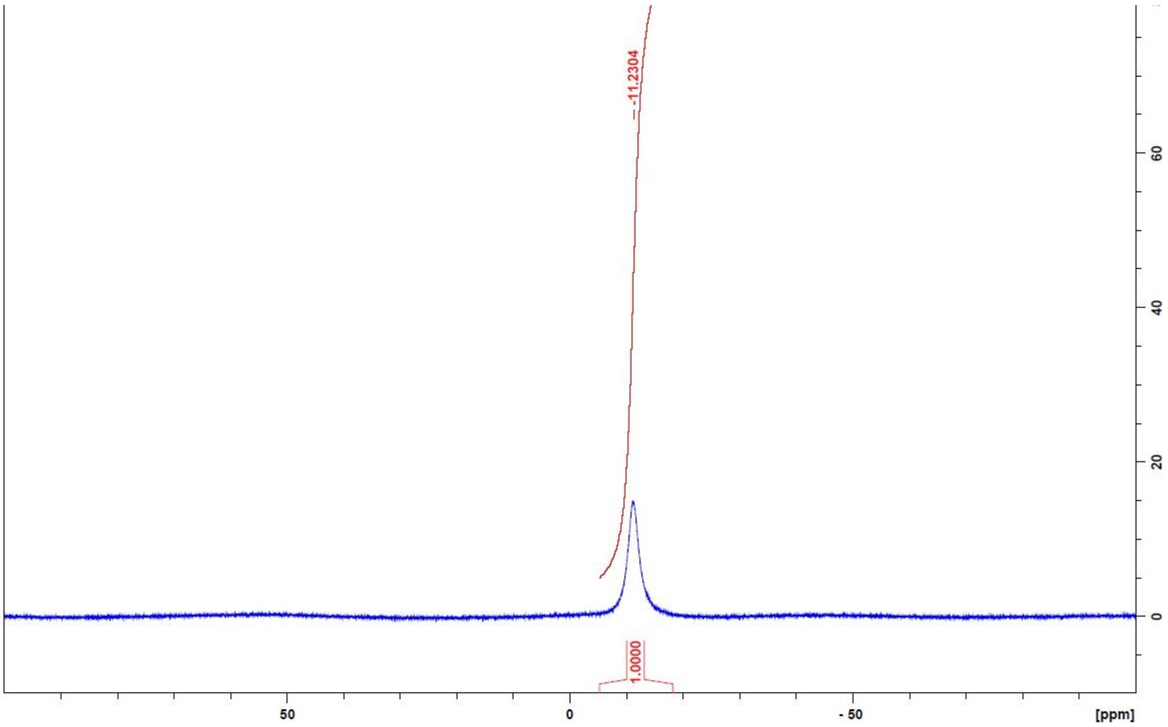

Figure S11. $^{11}B\{^{1}H\}$-NMR spectrum (CD_2Cl_2, 96.2 MHz) of compound **10**

2.4. Compound **11**.

Compound **10** (5.00 g, 13.42 mmol, 1.00 eq.) was suspended in tetrahydrofuran (100 mL), cooled to −78 °C and stirred for 10 min. A solution of NaHMDS {Na[N(SiMe$_3$)$_2$], 2.58 g, 14.09 mmol, 1.05 eq.} in THF (30 mL) was added dropwise with stirring. The mixture was allowed to warm to ambient temperature within 3 h with dissolution of the solid material and gradual precipitation of sodium chloride. Methyl iodide (CH$_3$I, 2.50 mL, 40.26 mmol, 3.00 eq.) was added at ambient temperature and the solution was stirred for 12 h. All volatiles were removed at reduced pressure The crude mixture was dissolved a two-phase-system composed of chloroform (200 mL) and saturated brine (150 mL). The organic phase was dried over MgSO$_4$ and the solvent removed in high vacuum. Compound **11** was obtained as a pale-yellow foam (4.98 g, 12.88 mmol, 96 %), which was sufficiently pure for further manipulation. Analytically pure samples were obtained by diffusion of *n*-pentane vapor into a solution of compound **11** in chloroform to afford a pale-yellow powder.

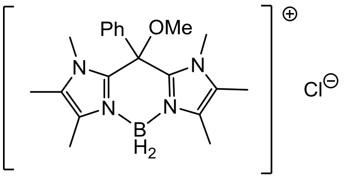

^{1}H-NMR (CD$_2$Cl$_2$, 400 MHz, 293 K): δ 2.20 (6 H, s, CMe), 2.27 (6 H, s, CMe), 2.5–4.0 (br, BH$_2$), 3.22 (3 H, s, OMe), 3.44 (6 H, s, NMe), 7.28–7.32 (2 H, s, aryl-CH), 7.38–7.42 (3 H, s, aryl-CH).

^{13}C{^{1}H}-NMR (CD$_2$Cl$_2$, 100.6 MHz, 293 K): δ 8.9 (CMe), 9.4 (CMe), 32.3 (NMe), 54.5 (OMe), 76.4 (*C*−OMe), 125.7 (aryl CH), 128.8, 129.3, 129.6 (aryl CH), 129.9 (aryl CH), 136.3 (N*C*Me), 137.3 (N*C*Me).

^{11}B{^{1}H}-NMR (CD$_2$Cl$_2$, 96.2 MHz, 293 K): δ −10.9 (BH$_2$, $\omega_{1/2}$ = 204 Hz).

^{11}B{^{1}H}-NMR (CD$_2$Cl$_2$, 96.2 MHz, 293 K): δ −10.9 (BH$_2$, t, $^1J_{BH}$ = 128 Hz).

IR: $\tilde{\upsilon}$ = 2970 (alkyl C−H), 2385 (B−H), 2358 (B−H), 2340 (B−H), 1630 (C=C), 1535 cm^{-1}.

MS (ESI): m/z = 351.20 [M–Cl]$^+$, 319.27 [M–Cl–MeOH]$^+$.

Elemental Analysis: Calculated: C 62.11, H, 7.30, N, 14.49. Found: C 62.34, H, 7.15, N, 14.53.

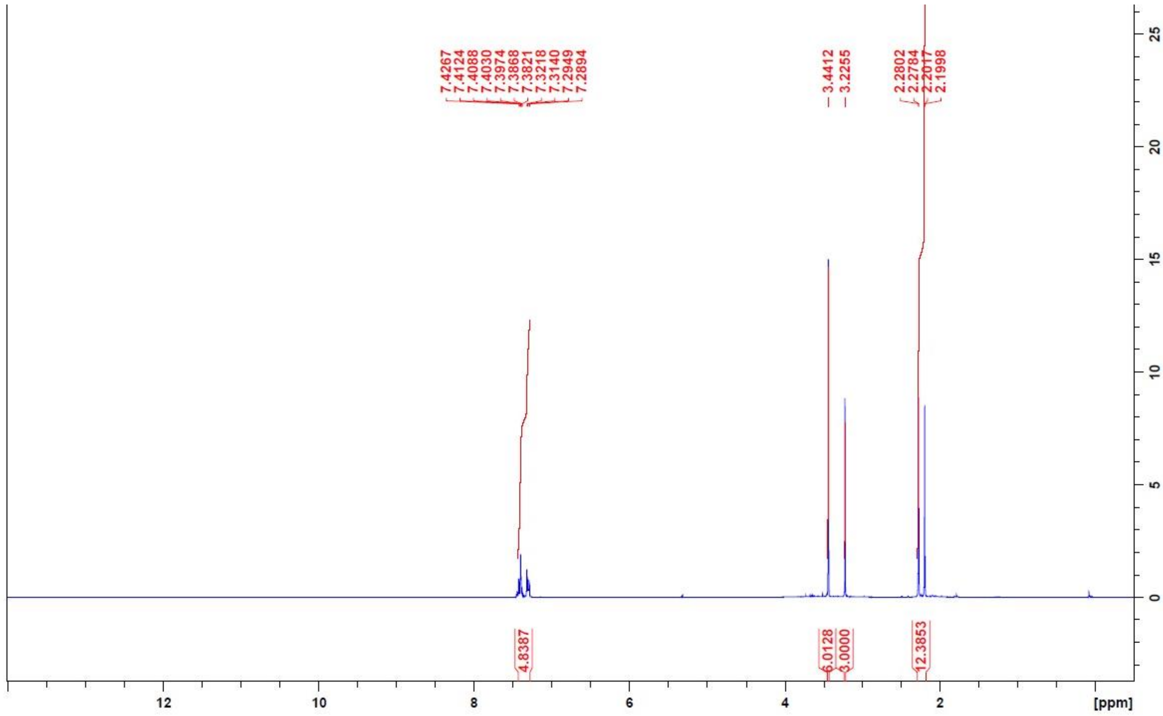

Figure S12. ^{1}H-NMR spectrum (CD_2Cl_2, 400 MHz) of compound **11**

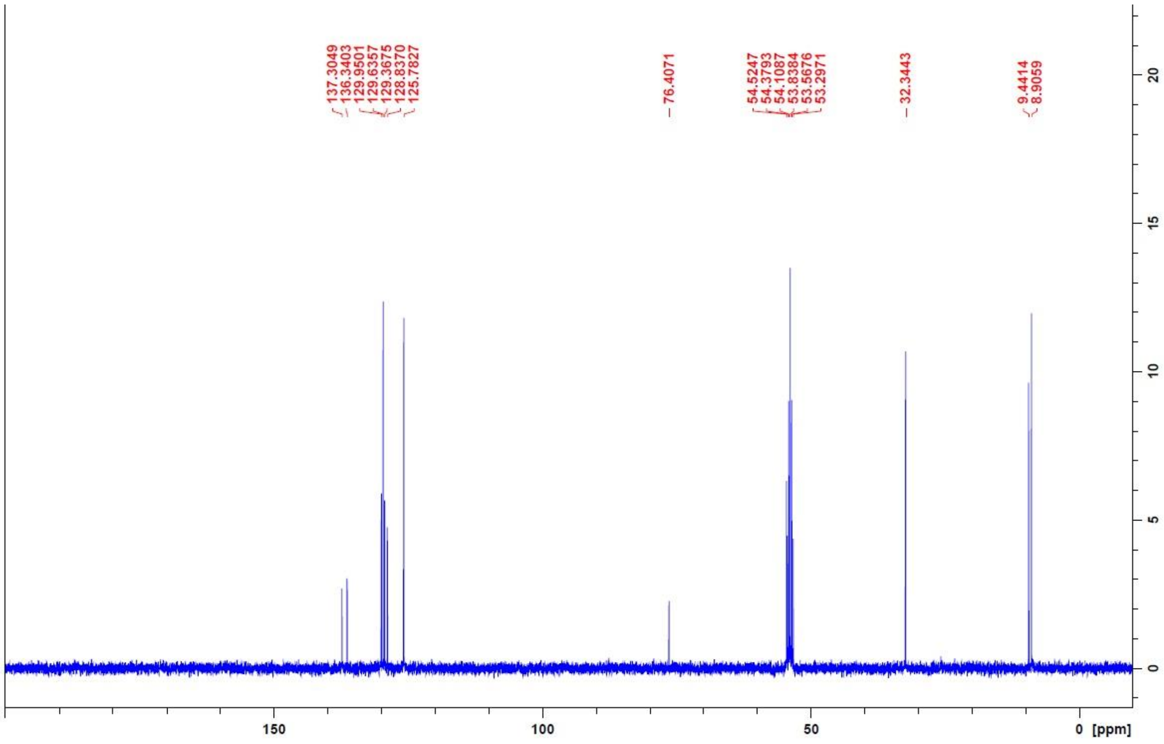

Figure S13. $^{13}C\{^{1}H\}$-NMR spectrum (CD_2Cl_2, 100.6 MHz) of compound **11**

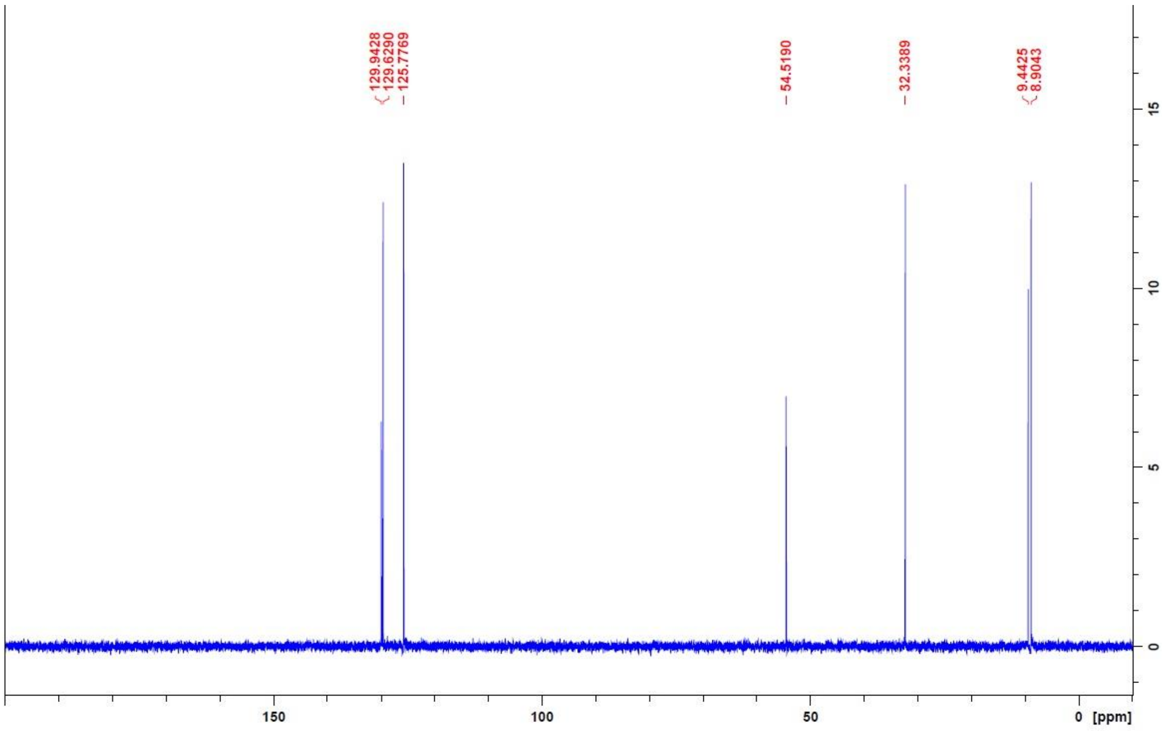

Figure S14. ^{13}C-DEPT-NMR spectrum (CD_2Cl_2, 100.6 MHz) of compound **11**

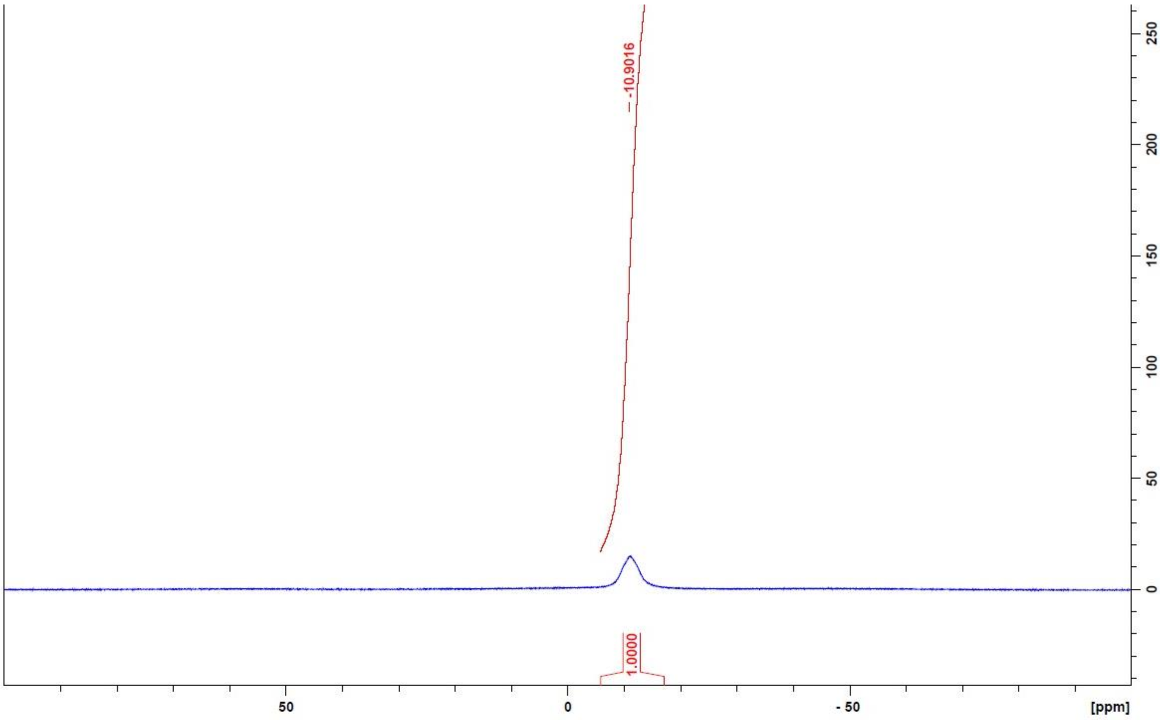

Figure S15. $^{11}B\{^1H\}$-NMR spectrum (CD_2Cl_2, 96.2 MHz) of compound **11**

2.5. Compound **6**.

Compound **11** (5.00 g, 12.93 mmol, 1.00 eq.) was suspended in tetrahydrofuran (100 mL), cooled to 0 °C and stirred for 10 min. Potassium graphite (KC_8, 7.00 g, 51.72 mmol, 4.00 eq.) was added in portions over 5 min. The mixture was stirred for 10 min and then filtered by frit-filtration. The orange filtrate was freed from all volatile material under high vacuum to afford compound **6** as a yellow powder (3.93 g, 12.28 mmol, 95 %). Analytically pure samples and crystals suitable for X-ray crystallography were obtained by diffusion of *n*-pentane vapor into a solution of compound **6** in tetrahydrofuran.

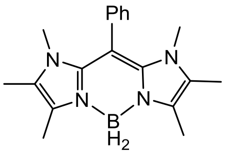

Chemical Formula: $C_{19}H_{25}BN_4$
Exact Mass: 320,22
Molecular Weight: 320,25
m/z: 320.22 (100.0%), 319.22 (24.8%), 321.22 (20.5%), 320.22 (5.1%), 322.22 (2.0%), 321.21 (1.5%)
Elemental Analysis: C, 71.26; H, 7.87; B, 3.38; N, 17.50

^{1}H-NMR (THF-D$_8$, 400 MHz, 293 K): δ 1.71 (6 H, s, CMe), 2.03 (6 H, s, CMe), 2.56 (6 H, s, NMe), 3.0–4.0 (br, BH_2), 7.20–7.34 (5 H, m, aryl-CH).

^{13}C{^{1}H}-NMR (THF-D$_8$, 100.6 MHz, 293 K): δ 8.4 (CMe), 9.2 (CMe), 32.1 (NMe), 68.0 (C−Ph), 118.6, 122.2, 126.9 (aryl CH), 128.1 (aryl CH), 137.0 (aryl CH), 138.9 (N*C*Me), 147.9 (N*C*Me).

^{11}B{^{1}H}-NMR (THF-D$_8$, 128.4 MHz, 293 K): δ −9.5 (BH_2, $\omega_{1/2}$ = 87 Hz).

^{11}B-NMR (THF-D$_8$, 128.4 MHz, 293 K): δ −9.5 (BH_2, t, $^{1}J_{BH}$ = 94 Hz).

IR: $\bar{\upsilon}$ = 2922, 2858 (both alkyl C−H), 2327 (B−H), 2324 (B−H), 2266 (B−H) cm^{-1}.

MS (EI): m/z = 319.2 [M–H]$^{+}$, 243.2 [M–Ph]$^{+}$.

Elemental Analysis: Calculated: C 71.26, H 7.87, N 17.50. Found: C 71.25, H 7.98, N 17.36.

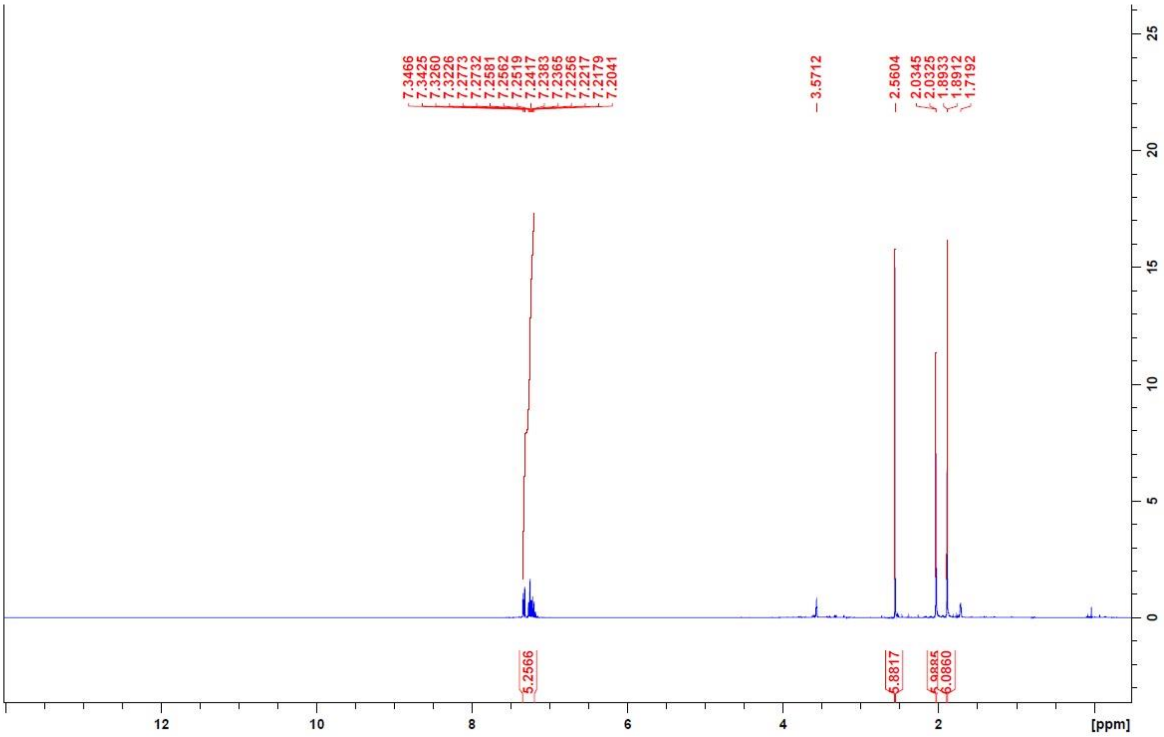

Figure S16. ^{1}H-NMR spectrum (CD_2Cl_2, 400 MHz) of compound **6**

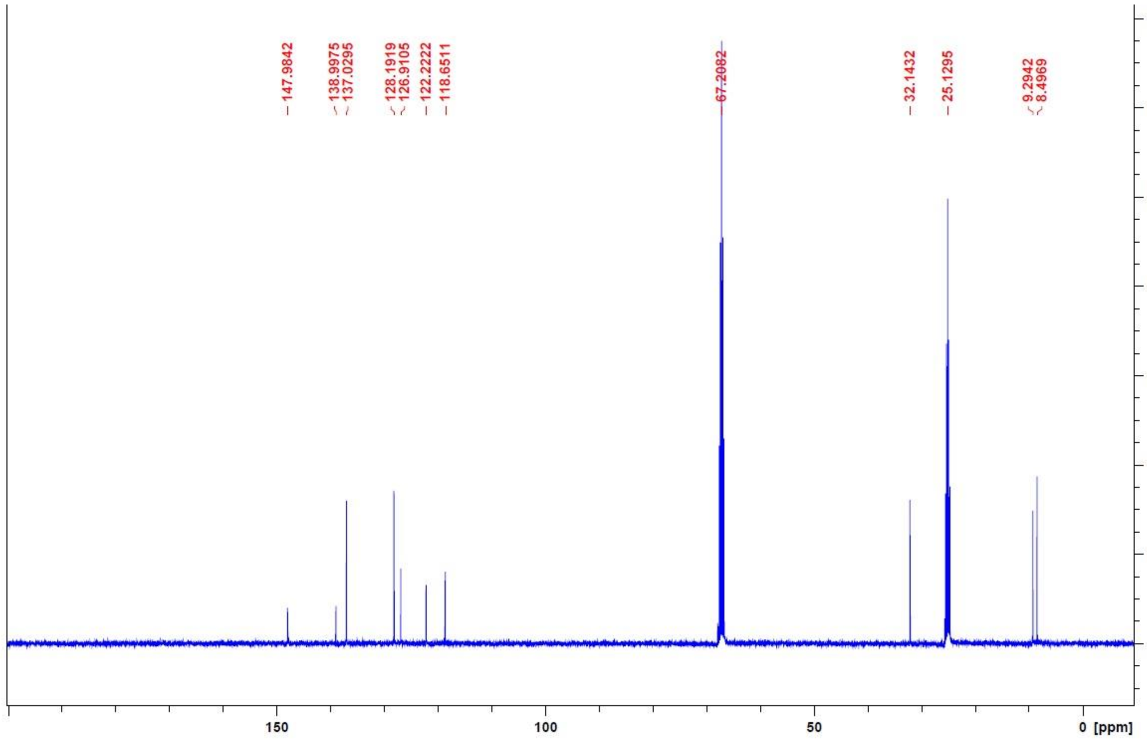

Figure S17. $^{13}C\{^{1}H\}$-NMR spectrum (CD_2Cl_2, 100.6 MHz) of compound **6**

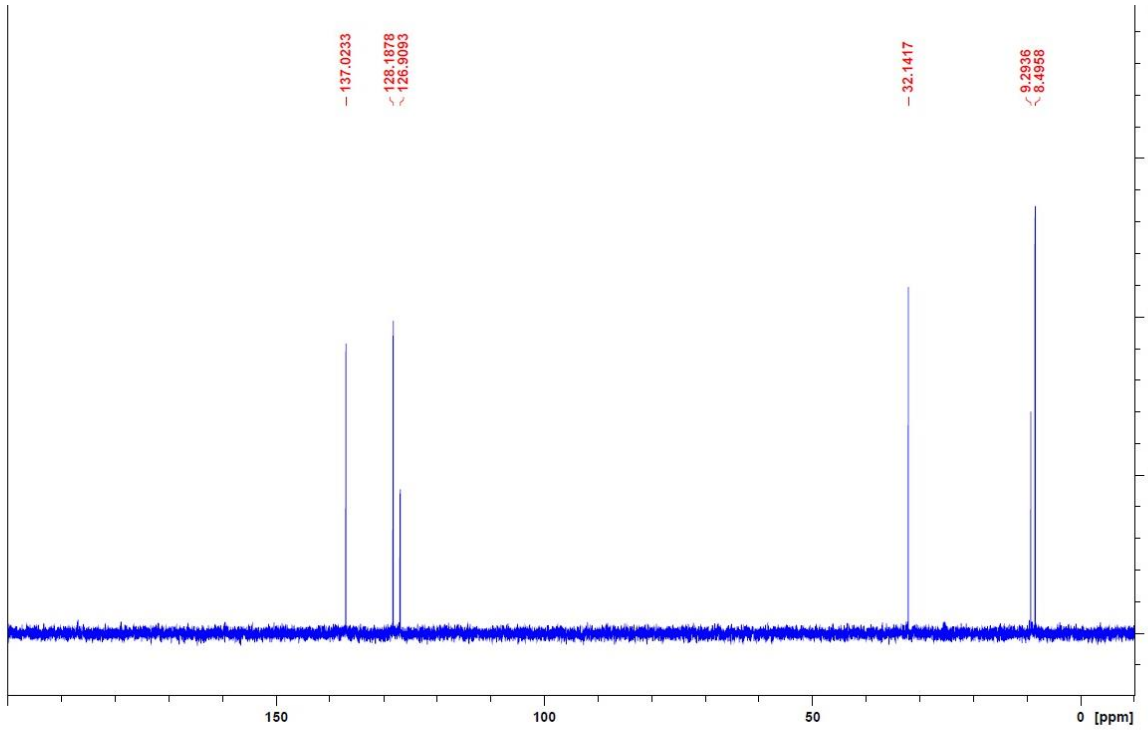

Figure S18. ^{13}C-DEPT-NMR spectrum (CD_2Cl_2, 100.6 MHz) of compound **6**

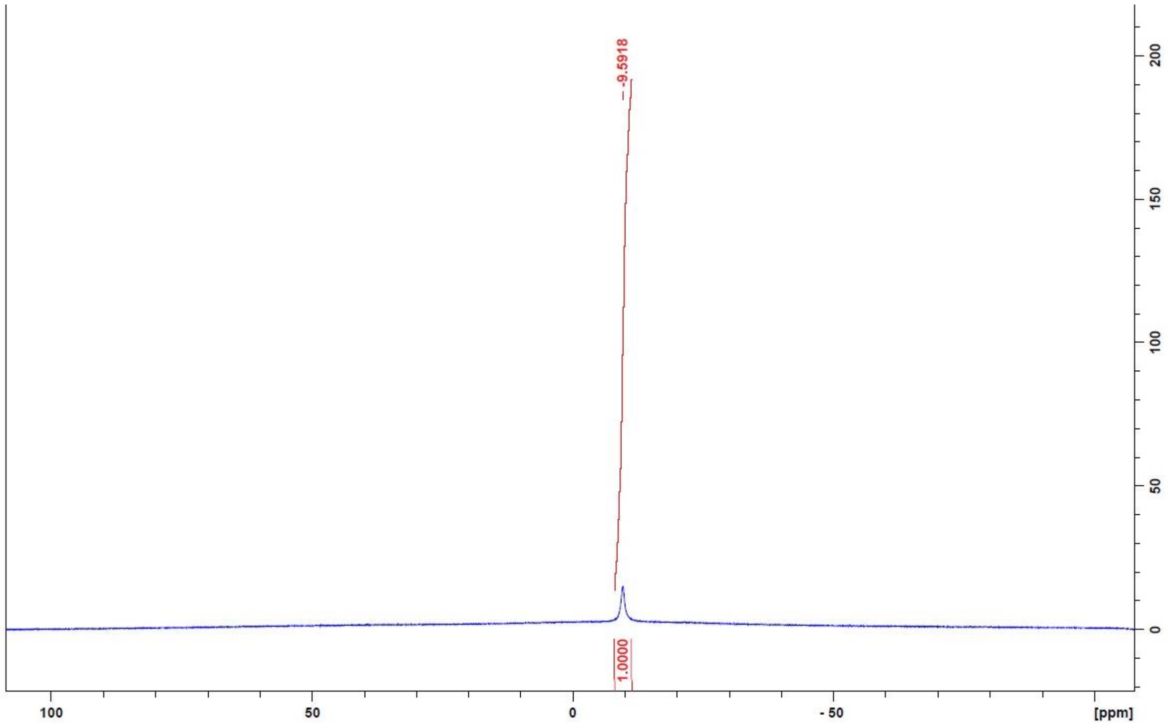

Figure S19. $^{11}B\{^{1}H\}$-NMR spectrum (CD_2Cl_2, 96.2 MHz) of compound **6**

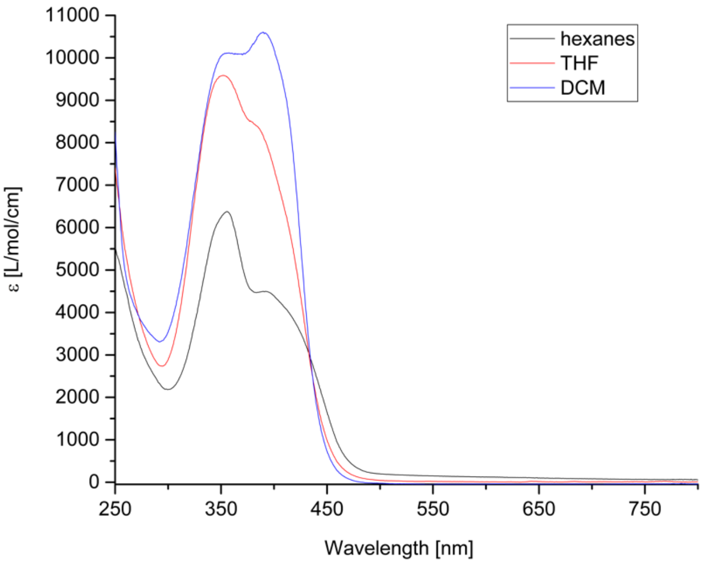

Figure S20. UV-VIS absorption spectra of compound **6**

2.6. Compound **12**.

Compound **6** (1.00 g, 3.12 mmol, 1.00 eq.) was dissolved in toluene (10 mL), and $BH_3{\cdot}SMe_2$ (237 mg, 6.24 mmol, 2.00 eq.) dissolved in toluene (1 mL) was added with stirring. The yellowish solution turned colorless immediately. After stirring for 30 min at ambient temperature all volatile material was removed under reduced pressure. Slow vapor diffusion of *n*-pentane into a solution of the crude product in toluene (5 mL) afforded colorless crystals of compound **12** (782 mg, 2.34 mmol, 75 %).

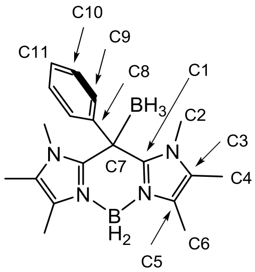

Chemical Formula: $C_{19}H_{28}B_2N_4$
Exact Mass: 334,25
Molecular Weight: 334,08
m/z: 334.25 (100.0%), 333.25 (49.7%), 335.25 (20.5%), 334.26 (10.2%), 332.26 (6.2%), 336.26 (2.0%), 335.25 (1.5%)
Elemental Analysis: C, 68.31; H, 8.45; B, 6.47; N, 16.77

^{1}H-NMR ($CDCl_3$, 300 MHz, 293 K): δ 0.5–2.0 (br, BH_3), 1.97 (6 H, s, C4-Me), 2.18 (6 H, s, C6-Me), 2.5–4.0 (br, BH_2), 3.00 (6 H, s, NMe), 7.18–7.25 (5 H, m, aryl-CH).

^{13}C{^{1}H}-NMR ($CDCl_3$, 100.6 MHz, 293 K): δ 8.5 (C4), 9.1 (C6), 31.6 (C2), 43.3 (C7), 123.6, 124.3 (both C1, C8), 126.7 (C10), 128.2 (C11), 132.2 (br, C9), 142.5, 150.4 (both C3, C5). The signal for C9 at 132.2 ppm sharpens upon heating (75 °C) and is due to dynamic motion of the phenyl moiety.

^{11}B{^{1}H}-NMR ($CDCl_3$, 96.2 MHz, 293 K): δ −11.2 (BH_2, $\omega_{1/2}$ = 197 Hz), −27.3 (BH_3, s, $\omega_{1/2}$ = 50 Hz).

^{11}B-NMR ($CDCl_3$, 96.2 MHz, 293 K): δ −11.2 (BH_2, br, s), −27.2 (BH_3, q, $^1J_{BH}$ = 89 Hz).

IR: = 2945 (alkyl C−H), 2330 (B−H), 2285 (B−H), 2233 (B−H), 1649 (C=C), 1517 cm^{-1}.

MS (EI): m/z = 320.2 [M–BH_3]$^+$, 319.2 [M–BH_3–H]$^+$.

Elemental Analysis: Calculated: C 68.31, H 8.45, N 16.77. Found: C 68.03, H 8.40, N 16.79.

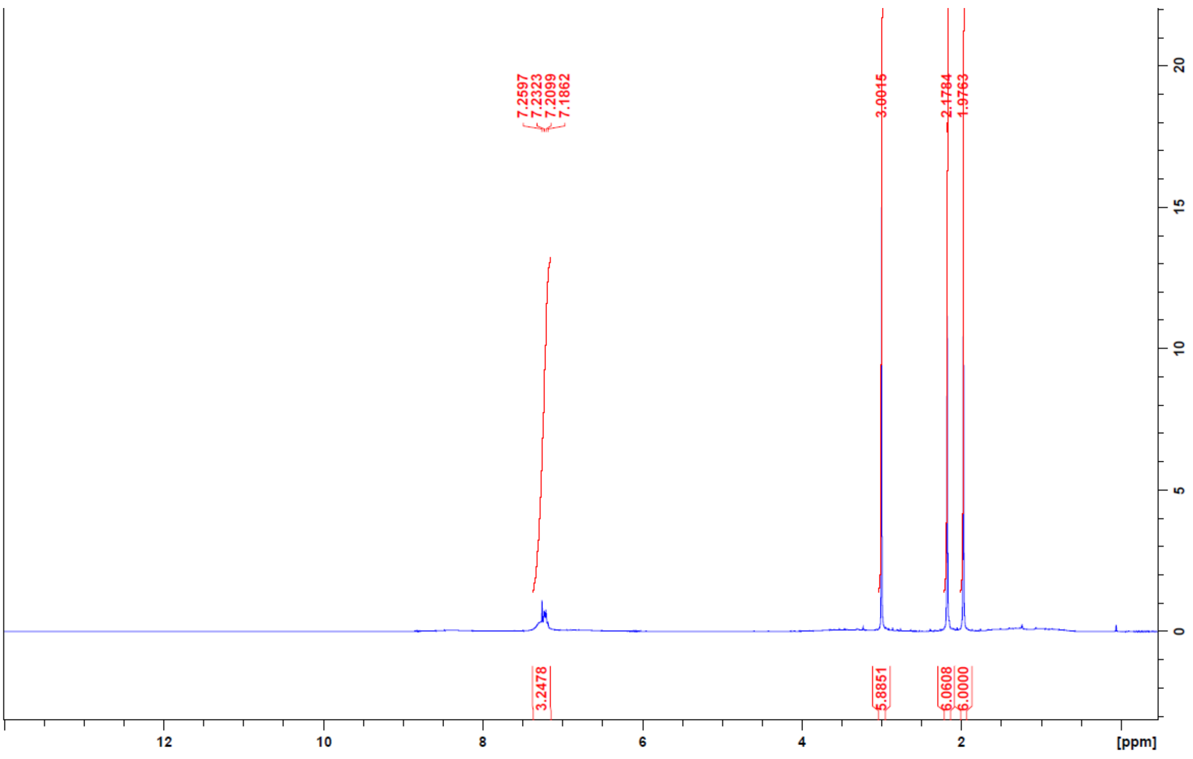

Figure S21. ^{1}H-NMR spectrum ($CDCl_3$, 300 MHz) of compound **12**

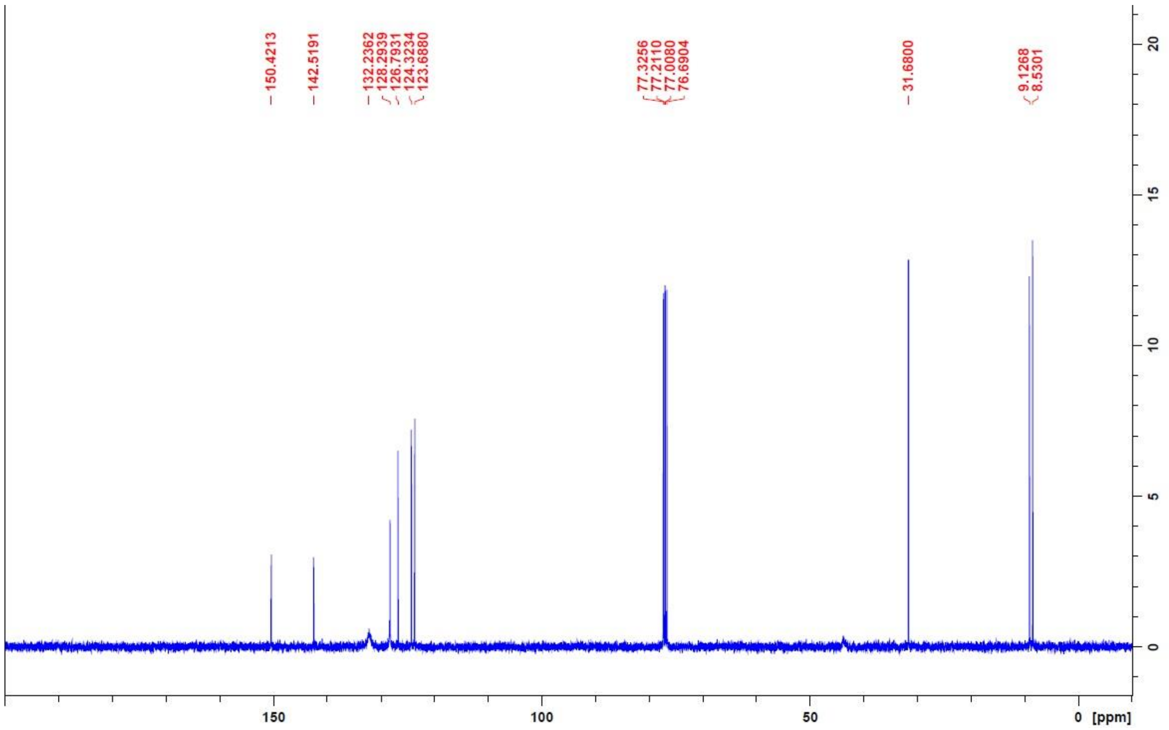

Figure S22. ^{13}C{^{1}H}-NMR spectrum ($CDCl_3$, 100.6 MHz) of compound **12**

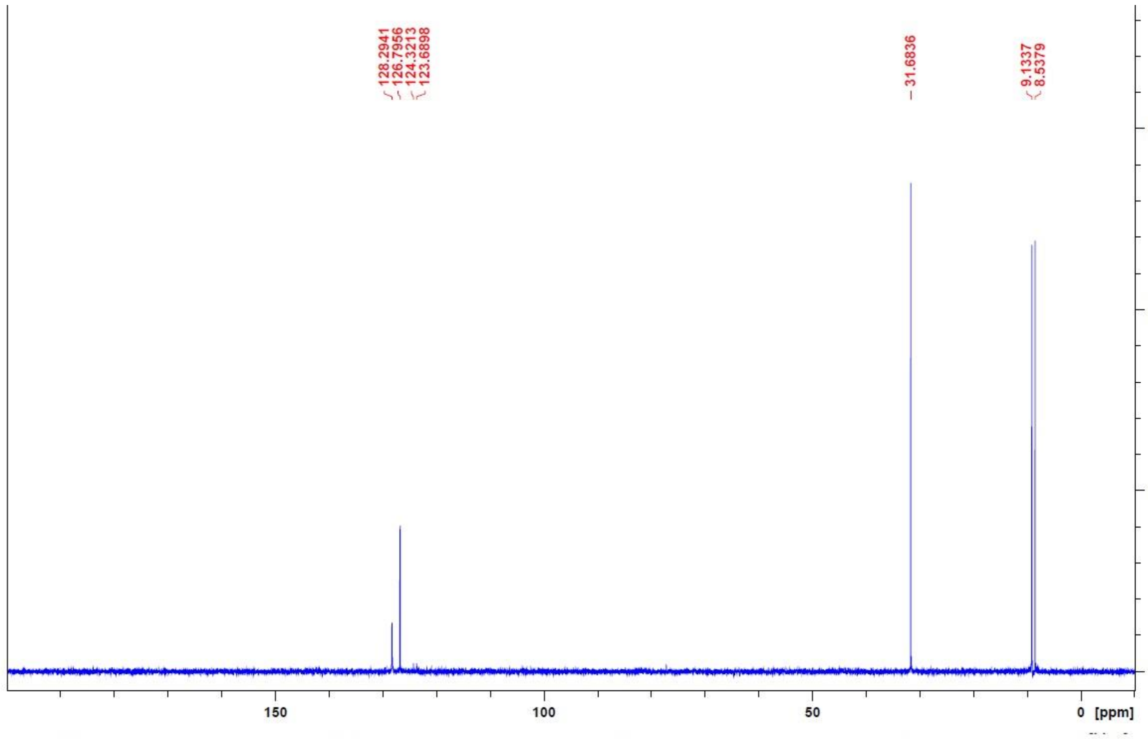

Figure S23. ^{13}C-DEPT-NMR spectrum ($CDCl_3$, 100.6 MHz) of compound **12**

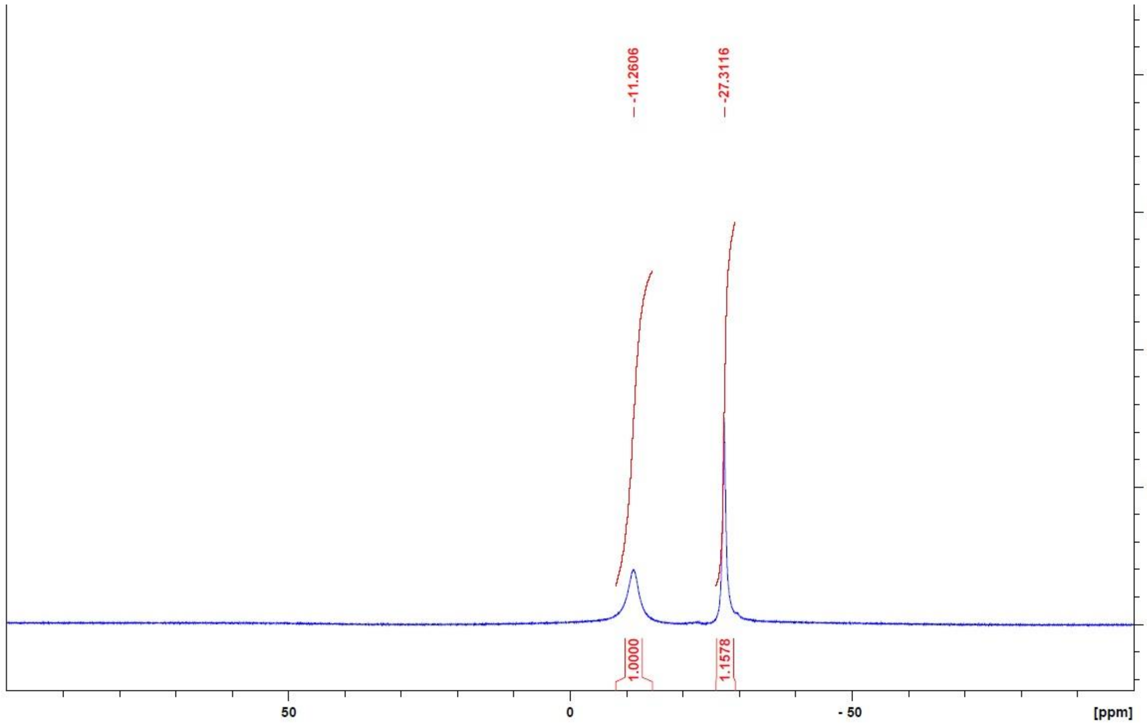

Figure S24. $^{11}B\{^{1}H\}$-NMR spectrum ($CDCl_3$, 96.2 MHz) of compound **12**

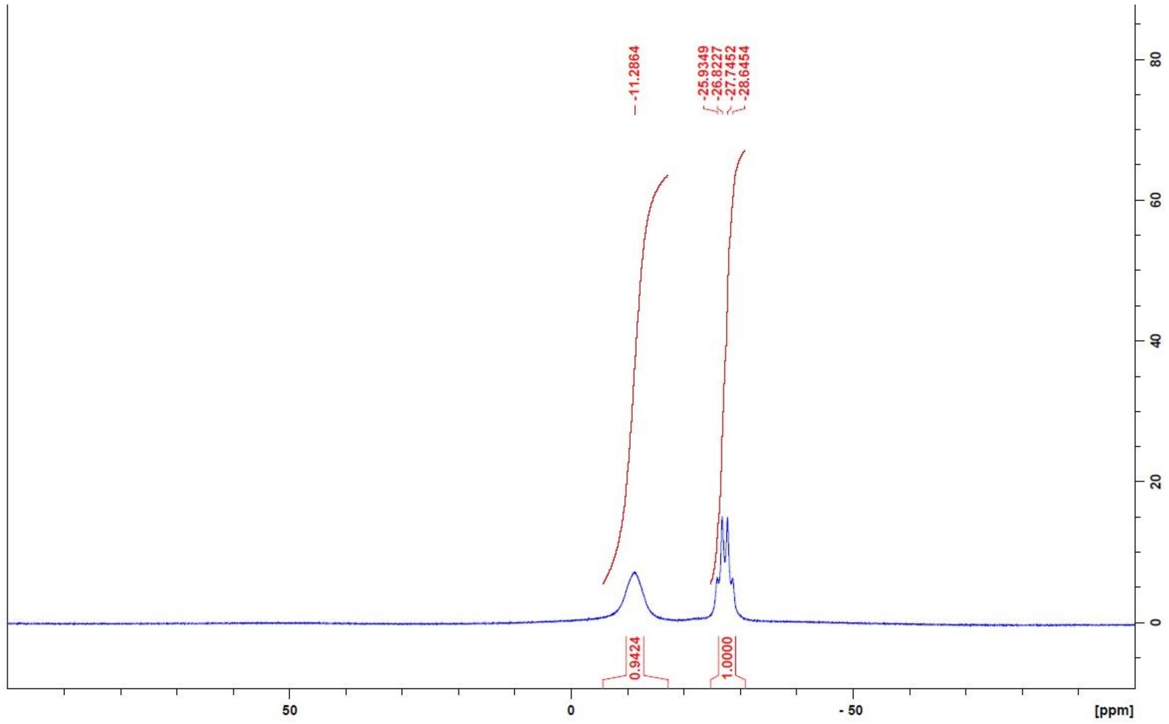

Figure S25. ^{11}B-NMR spectrum ($CDCl_3$, 96.2 MHz) of compound **12**

2.7. Compound **13**.

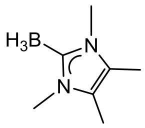

Compound **13** was prepared according to a literature procedure.[2]

^{1}H-NMR (C_6D_6, 300 MHz, 293 K): δ 0.95 (3 H, BH_3, q, $^{1}J_{BH}$ = 87 Hz), 2.09 (6 H, s, CMe), 3.53 (6 H, s, NMe).

^{11}B{^{1}H}-NMR (C_6D_6, 96.2 MHz, 293 K): δ −11.2 (BH_3, $\omega_{1/2}$ = 5 Hz).

^{11}B-NMR (C_6D_6, 96.2 MHz, 293 K): δ −11.2 (BH_3, q, $^{1}J_{BH}$ = 87 Hz).

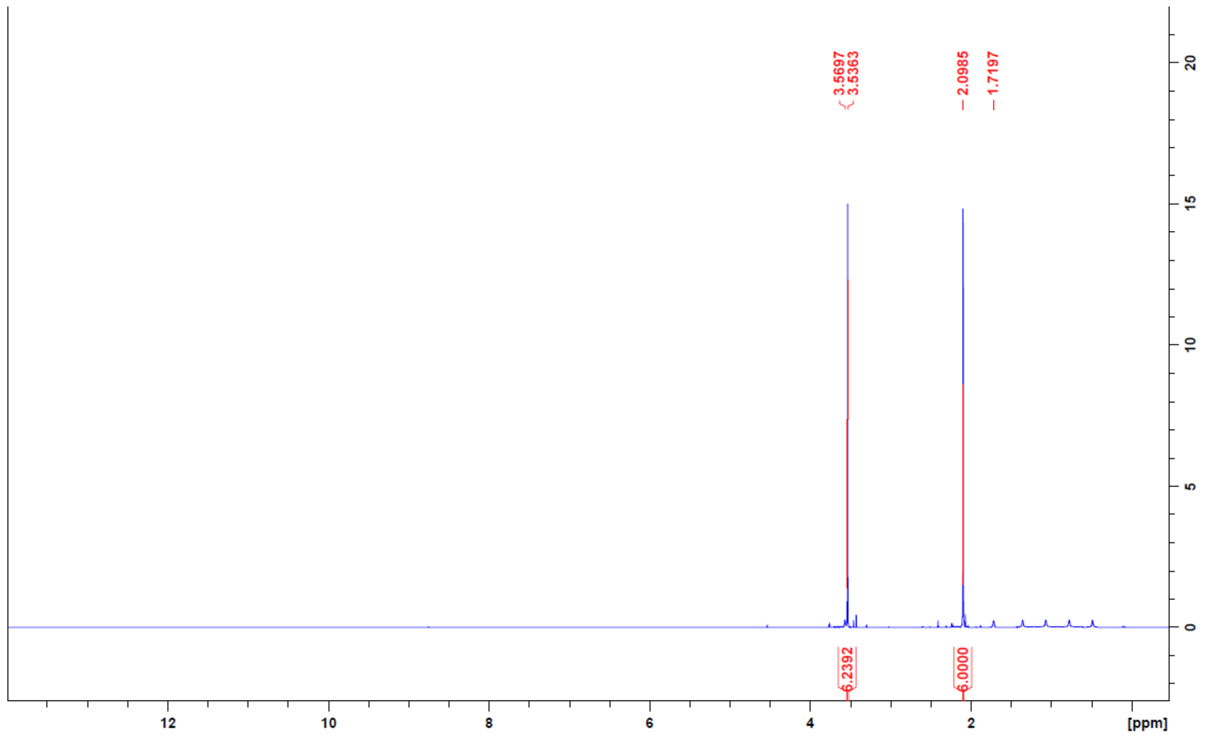

Figure S26. ^{1}H-NMR spectrum (C_6D_6, 300 MHz) of compound **13**

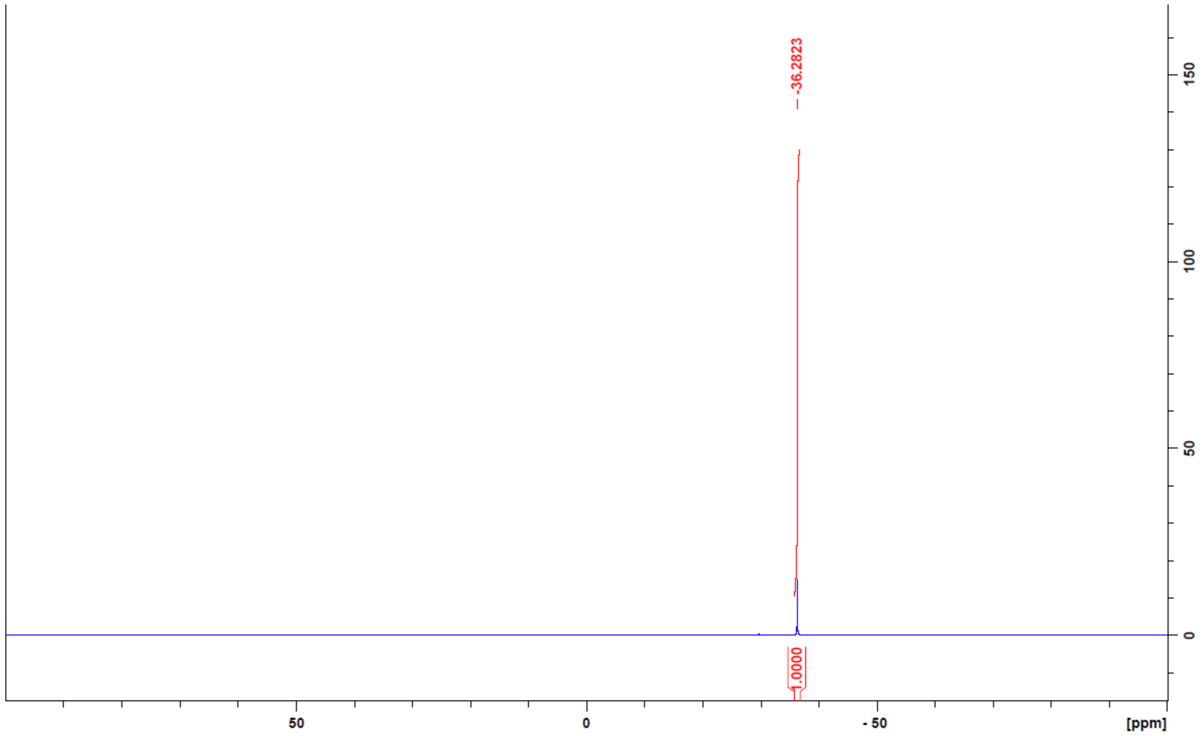

Figure S27. $^{11}B\{^1H\}$-NMR spectrum (C_6D_6, 96.2 MHz) of compound **13**

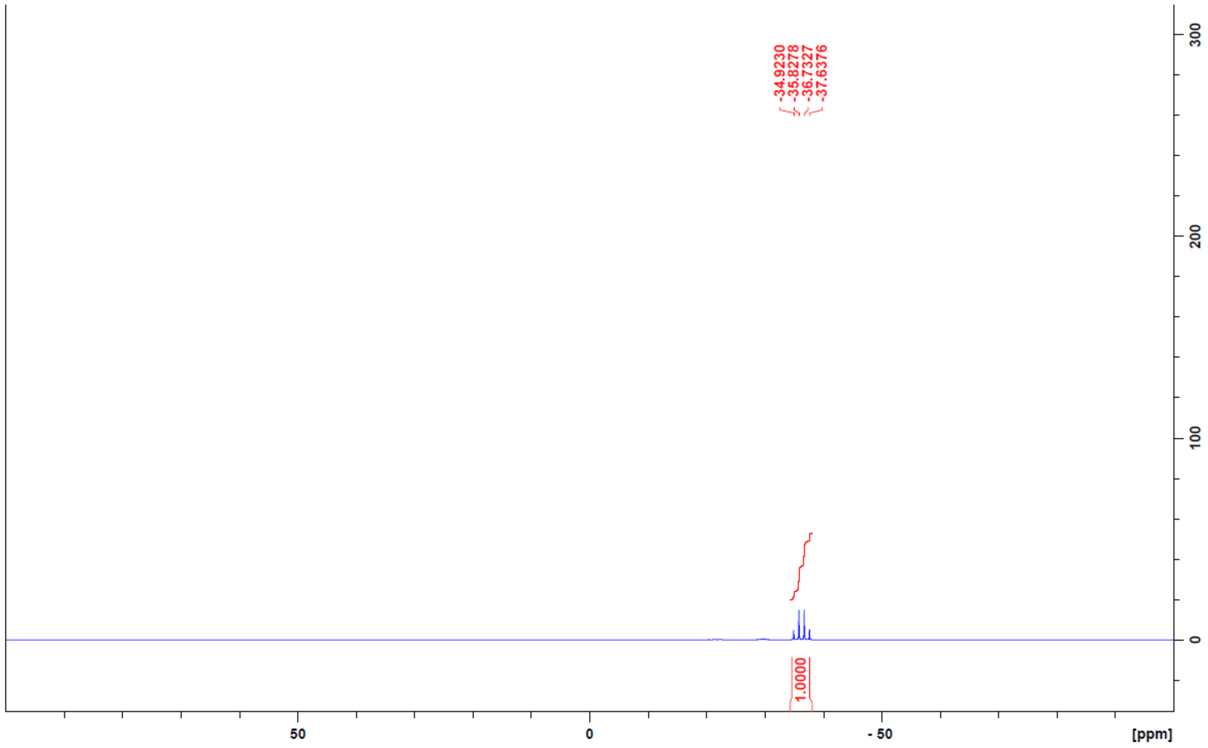

Figure S28. ^{11}B-NMR spectrum (C_6D_6, 96.2 MHz) of compound **13**

2.8. Compound **14**

Compound **6** (1.00 g, 3.12 mmol, 1.00 eq.) was dissolved in toluene (10 mL), and Mes-BH_2 (Mes = 2,4,6-$Me_3C_6H_2$, 618 mg, 6.24 mmol, 1.50 eq.) dissolved in toluene (1 mL) was added with stirring. The yellowish solution turned colorless immediately. After stirring for 30 min at ambient temperature all volatile material was removed under reduced pressure. Slow vapor diffusion of *n*-pentane into a solution of the crude product in toluene (4 mL) afforded colorless crystals of compound **12** (990 mg, 2.18 mmol, 70 %).

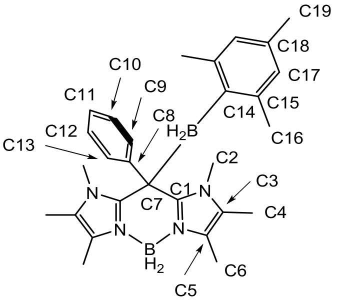

Chemical Formula: $C_{28}H_{39}B_2N_4$
Exact Mass: 453,34
Molecular Weight: 453,27
m/z: 453.34 (100.0%), 452.34 (49.7%), 454.34 (21.6%), 453.34 (10.2%), 454.34 (8.7%), 451.34 (6.2%), 453.34 (4.8%), 455.34 (2.1%), 455.34 (2.0%), 454.35 (2.0%), 454.33 (1.5%), 452.35 (1.3%)
Elemental Analysis: C, 74.20; H, 8.67; B, 4.77; N, 12.36

^{1}H-NMR (C_6D_6, 300 MHz, 293 K): δ 1.0−2.5 (br, BH_2Mes), 1.28 (6 H, s, C4-Me), 1.96 (6 H, s, C6-Me), 2.09 (6 H, s, *ortho*-Me), 2.36 (3 H, s, *para*-Me), 2.5−4.0 (br, BH_2), 2.77 (6 H, s, NMe), 6.59 (1 H, br, s, aryl-CH), 6.87 (1H, br, s, aryl-CH), 6.92 (2 H, s, C17−H), 7.05 (1 H, m, aryl-CH), 7.33 (1 H, br, s, aryl-CH), 9.20 (1 H, br, s, aryl-CH).

^{13}C{^{1}H}-NMR (C_6D_6, 125.8 MHz, 293 K): δ 7.9 (C4), 9.2 (C6), 21.5 (C19), 22.3 (C16), 31.6 (C2), 123.6, 124.9 (both C1, C8), 126.9 (C11), 127.7 (C17), 128.5, 129.3 (both C10, C12), 132.3, 134.1 (both C9, C13) 132.1, 143.4 (both C15, C18), 145.5, 150.4 (both C3, C5). Not observed C7, C14.

^{11}B{^{1}H}-NMR (C_6D_6, 96.2 MHz, 293 K): δ −11.2 (BH_2, $\omega_{1/2}$ = 189 Hz), −19.5 (Mes−BH_2, $\omega_{1/2}$ = 89 Hz).

^{11}B-NMR (C_6D_6, 96.2 MHz, 293 K): δ −10.9 (BH_2, br), −19.5 (Mes−BH_2, t, $^1J_{BH}$ = 85 Hz).

IR: $\tilde{\upsilon}$ = 2982 (alkyl C−H), 2369 (B−H), 2328 (B−H), 2282 (B−H), 1647 (C=C), 1518 cm^{-1}.

MS (EI): m/z = 319.2 [M–BH_2Mes]$^+$, 243.2 [M–BH_2Mes–Ph]$^+$.

Elemental Analysis: Calculated: C 74.36, H 8.47, N 12.39. Found: C 74.21, H 8.36, N 12.20.

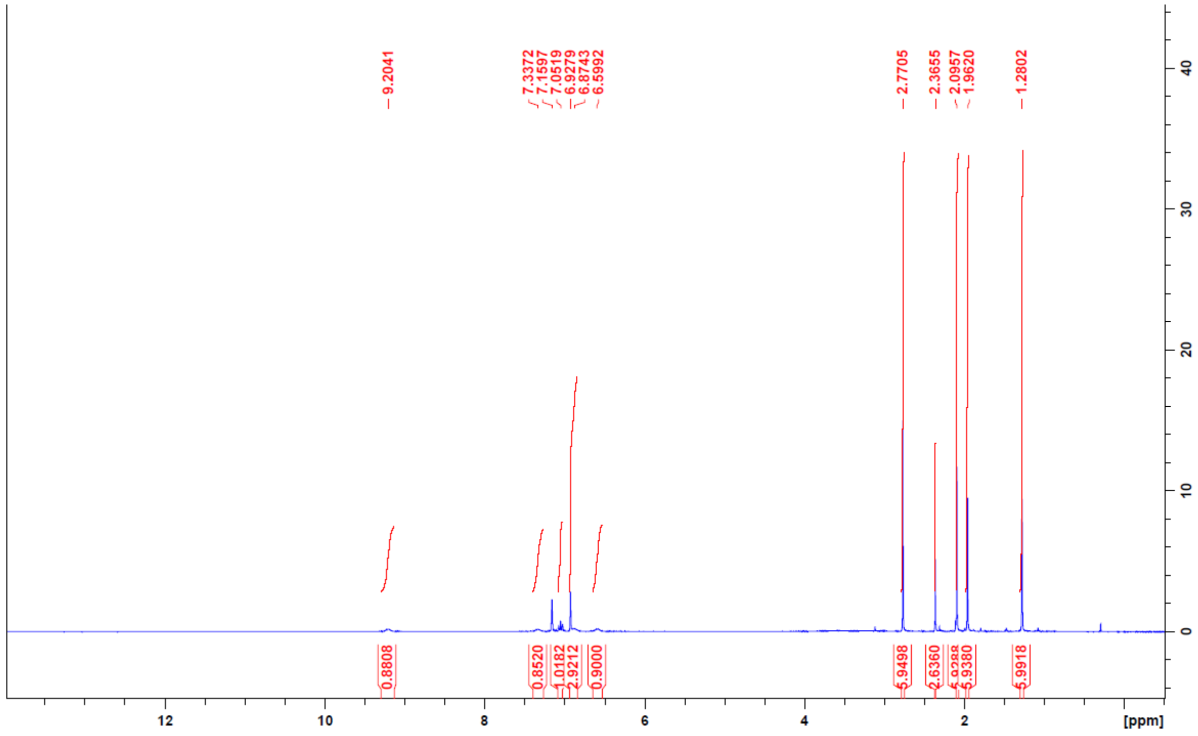

Figure S29. ^{1}H-NMR spectrum (C_6D_6, 300 MHz) of compound **14**

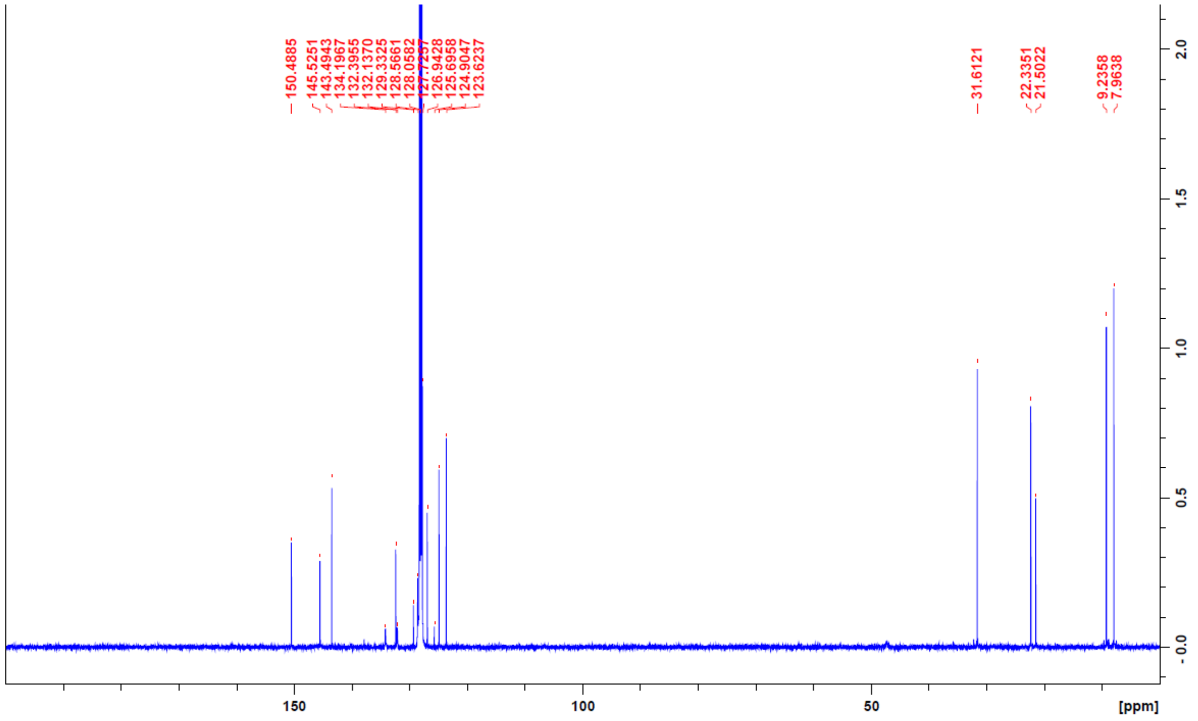

Figure S30. $^{13}C\{^{1}H\}$-NMR spectrum (C_6D_6, 125.8 MHz) of compound **14**

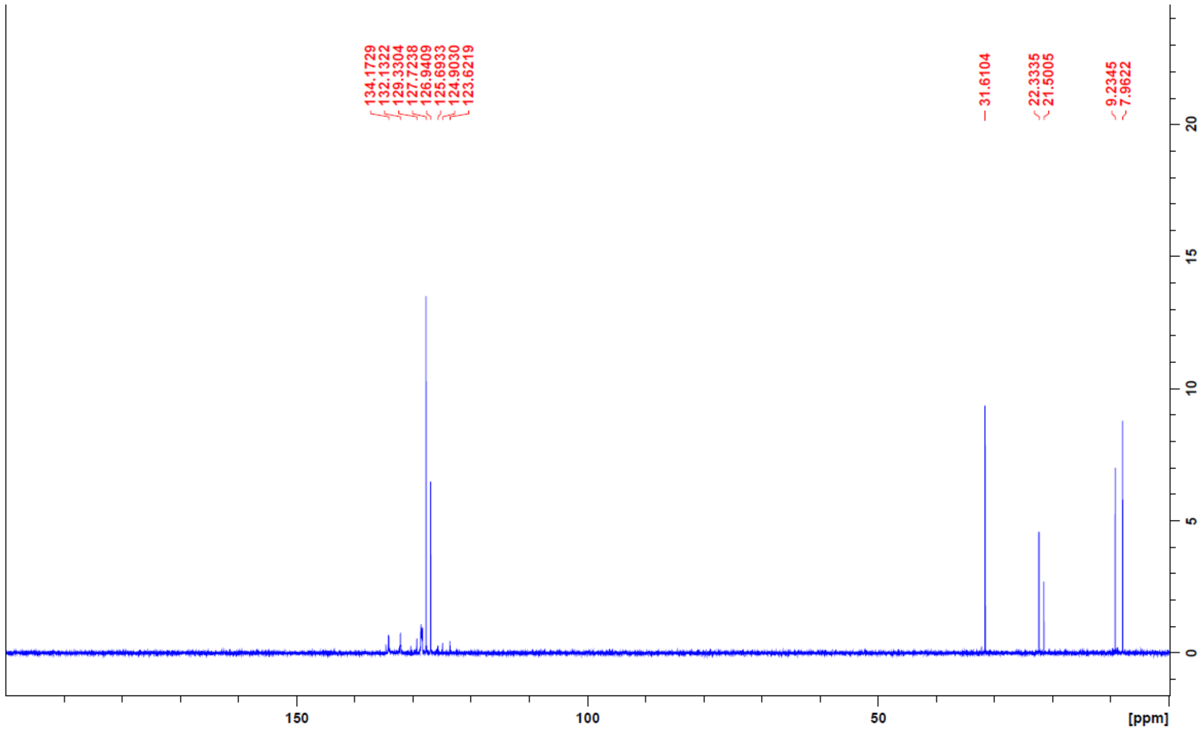

Figure S31. ^{13}C-DEPT-NMR spectrum (C_6D_6, 125.8 MHz) of compound **14**

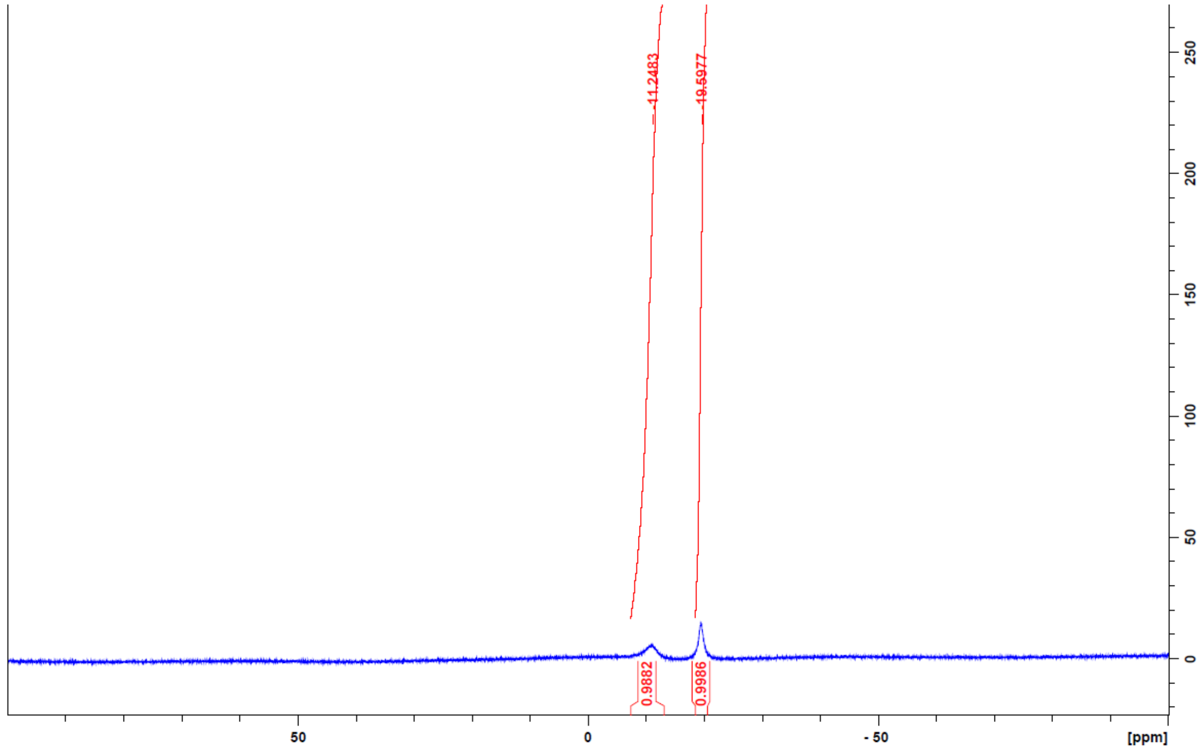

Figure S32. $^{11}B\{^1H\}$-NMR spectrum (C_6D_6, 96.2 MHz) of compound **14**

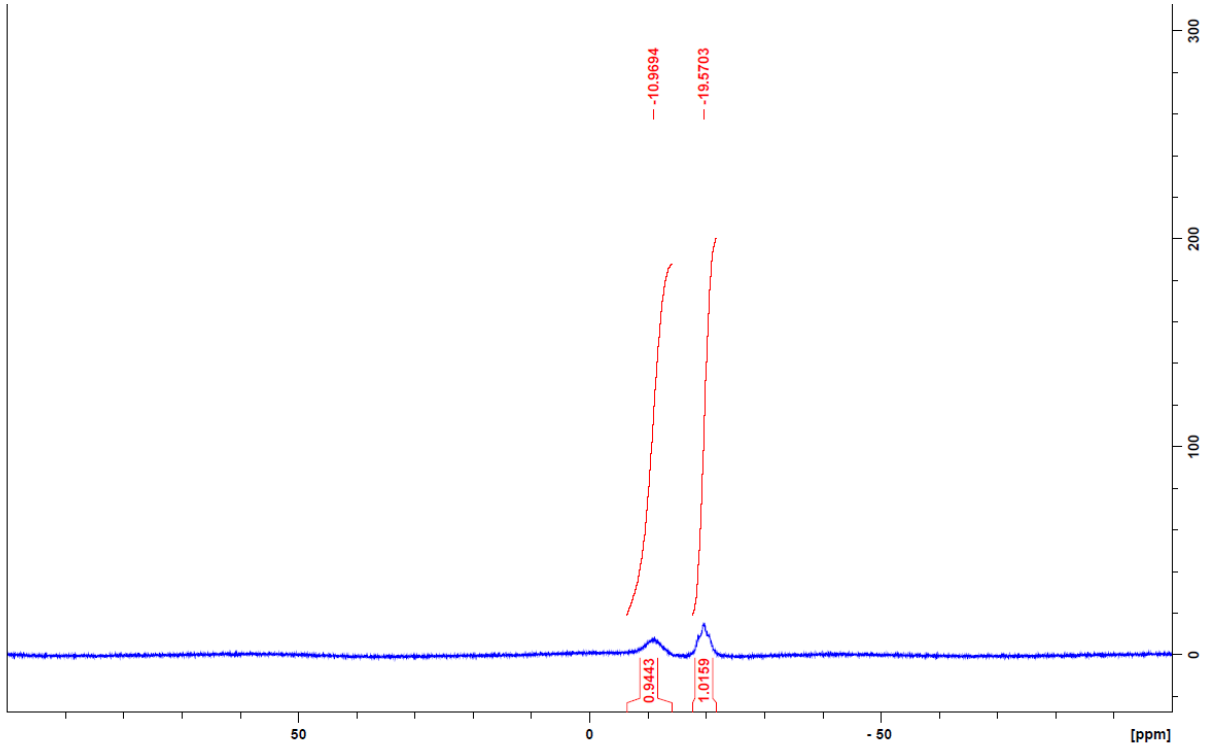

Figure S33. ^{11}B-NMR spectrum (C_6D_6, 96.2 MHz) of compound **14**

2.9. Compound **15**.

The *N*-heterocyclic carbene IMe^{Me} (500 mg, 4.03 mmol, 1.00 eq.) was dissolved in toluene (5 mL) and Mes-BH$_2$ (Mes = 2,4,6-Me$_3$C$_6$H$_2$, 638 mg, 4.84 mmol, 1.20 eq.) dissolved in toluene (5 mL) was added with stirring at ambient temperature. After 30 min all volatile material was removed under reduced pressure. The residue was washed with *n*-pentane (2 × 10 mL) to obtain a pale-yellow material **15** (500 mg, 3.22 mmol, 80 %).

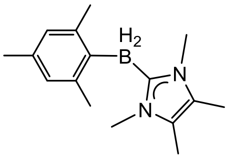

Chemical Formula: $C_{16}H_{25}BN_2$
Exact Mass: 256,21
Molecular Weight: 256,20
m/z: 256.21 (100.0%), 255.21 (24.8%), 257.21 (17.3%), 256.22 (4.3%)
Elemental Analysis: C, 75.01; H, 9.84; B, 4.22; N, 10.93

^{1}H-NMR (C_6D_6, 300 MHz, 293 K): δ 1.16 (6 H, s, CMe), 1.5−3.0 (br, BH$_2$), 2.41 (3 H, s, *para*-CH$_3$), 2.60 (6 H, s, *ortho*-Me), 2.99 (6 H, s, NMe) 7.09 (2 H, s, aryl-CH).

^{13}C{^{1}H}-NMR (CD_2Cl_2, 100.6 MHz, 293 K): δ 8.8 (C−CH$_3$), 20.9, 23.9 (both Mes−*C*H$_3$), 32.2 (NMe), 123.5 (*C*−CH$_3$), 127.6 (aryl-CH), 132.8 (aryl-C), 142.1 (aryl-C). Not observed B−C.

^{11}B{^{1}H}-NMR (C_6D_6, 96.2 MHz, 293 K): δ −27.2 (BH$_2$, $\omega_{1/2}$ = 12 Hz).

^{11}B-NMR (C_6D_6, 96.2 MHz, 293 K): −29.4 (BH$_2$, t, $^1J_{BH}$ = 84 Hz).

IR: $\tilde{\upsilon}$ = 2923 (alkyl C−H), 2291 (B−H), 1659 (C=C) cm^{-1}.

MS (EI): m/z = 256.2 [M–H]$^+$, 241.2 [M–Me]$^+$.

Elemental Analysis: Calculated: C 75.01, H 9.84, N 10.93. Found: C 75.25, H 9.79, N 10.98.

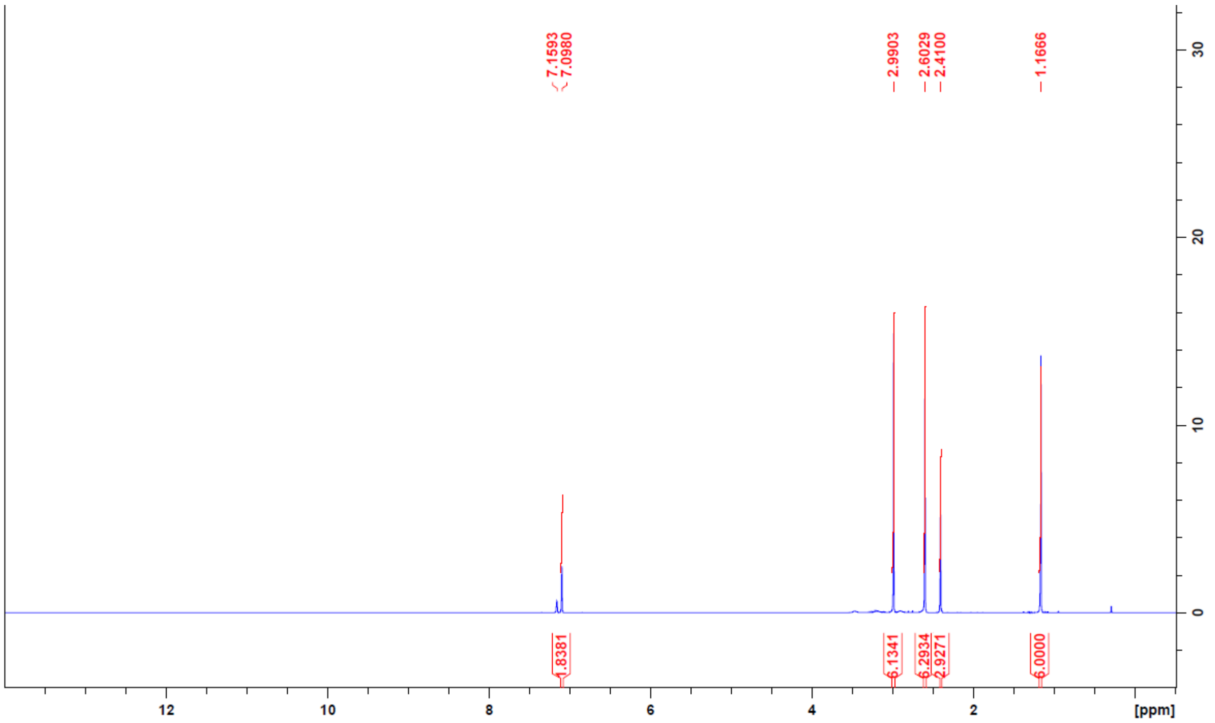

Figure S34. ^{1}H-NMR spectrum (C_6D_6, 300 MHz) of compound **15**

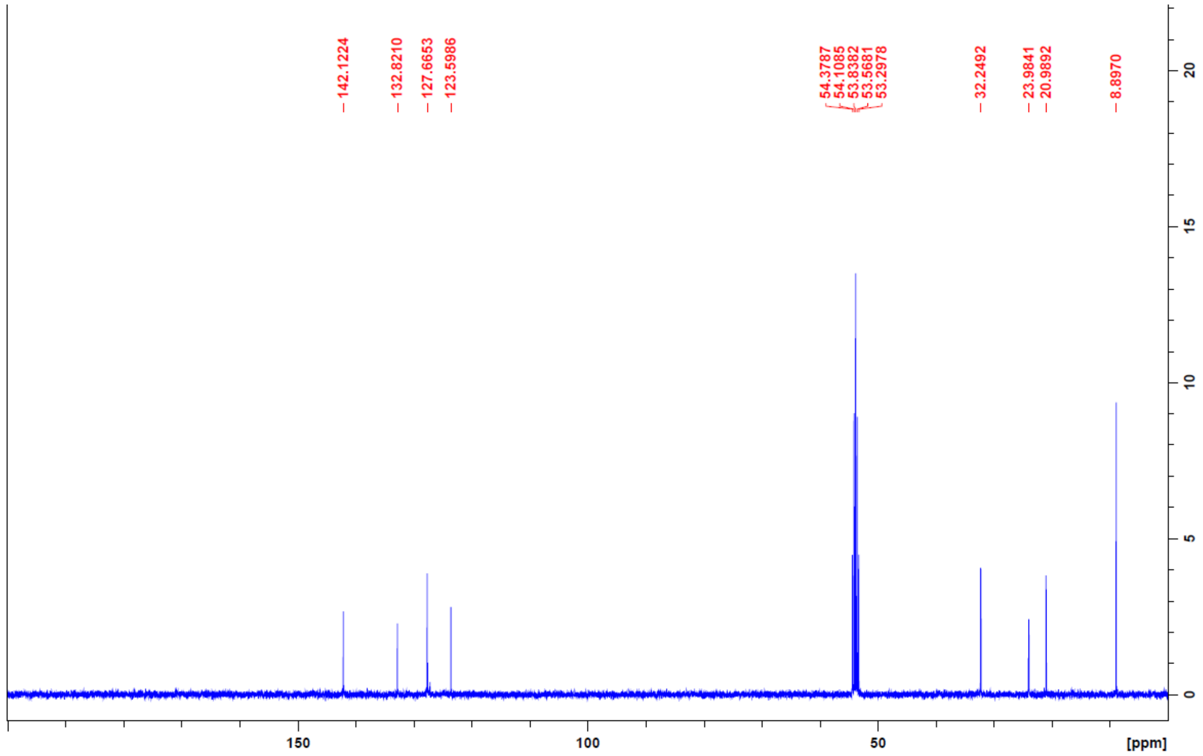

Figure S35. $^{13}C\{^{1}H\}$-NMR spectrum (CD_2Cl_2, 100.6 MHz) of compound **15**

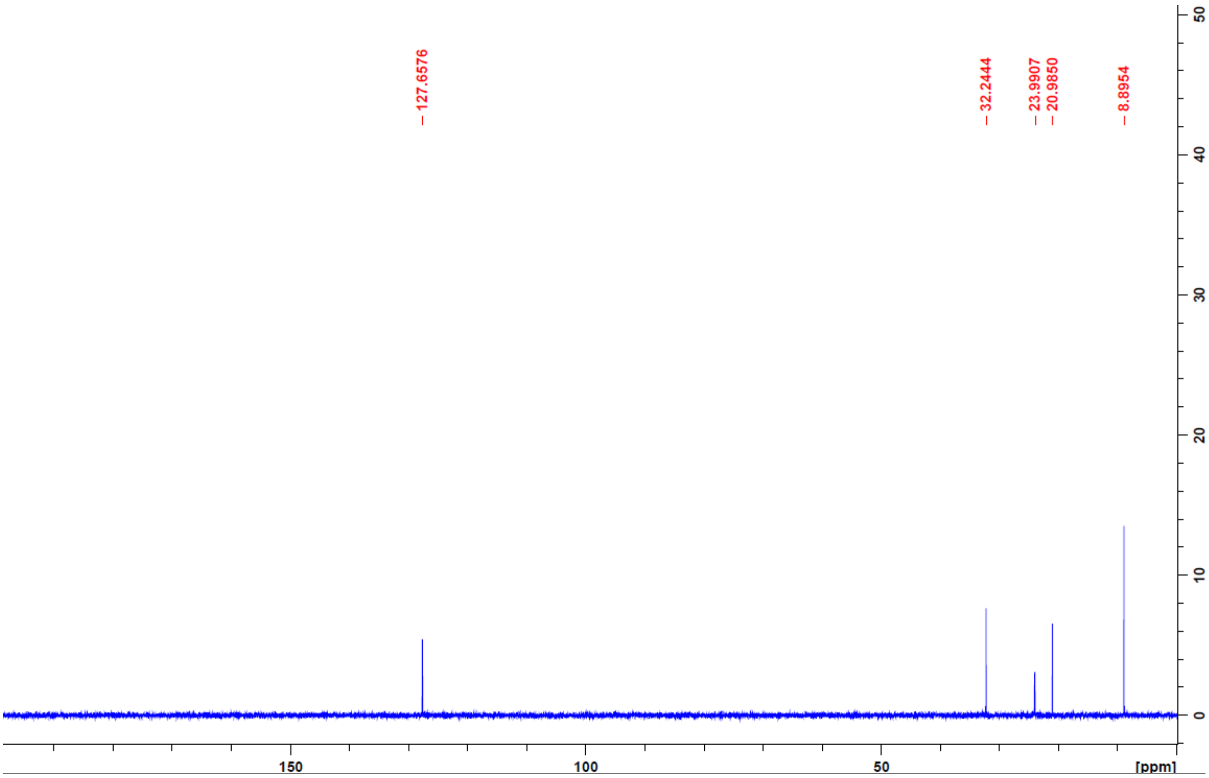

Figure S36. ^{13}C-DEPT-NMR spectrum (CD_2Cl_2, 100.6 MHz) of compound **15**

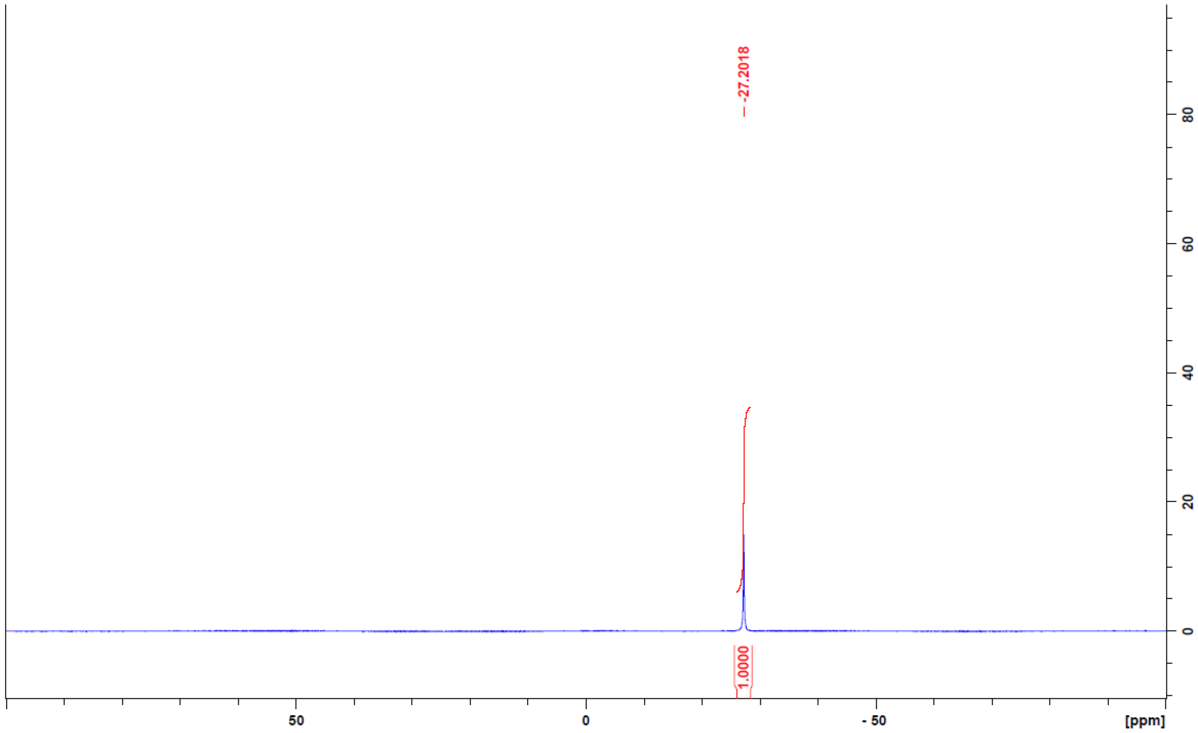

Figure S37. $^{11}B\{^1H\}$-NMR spectrum (C_6D_6, 96.2 MHz) of compound **15**

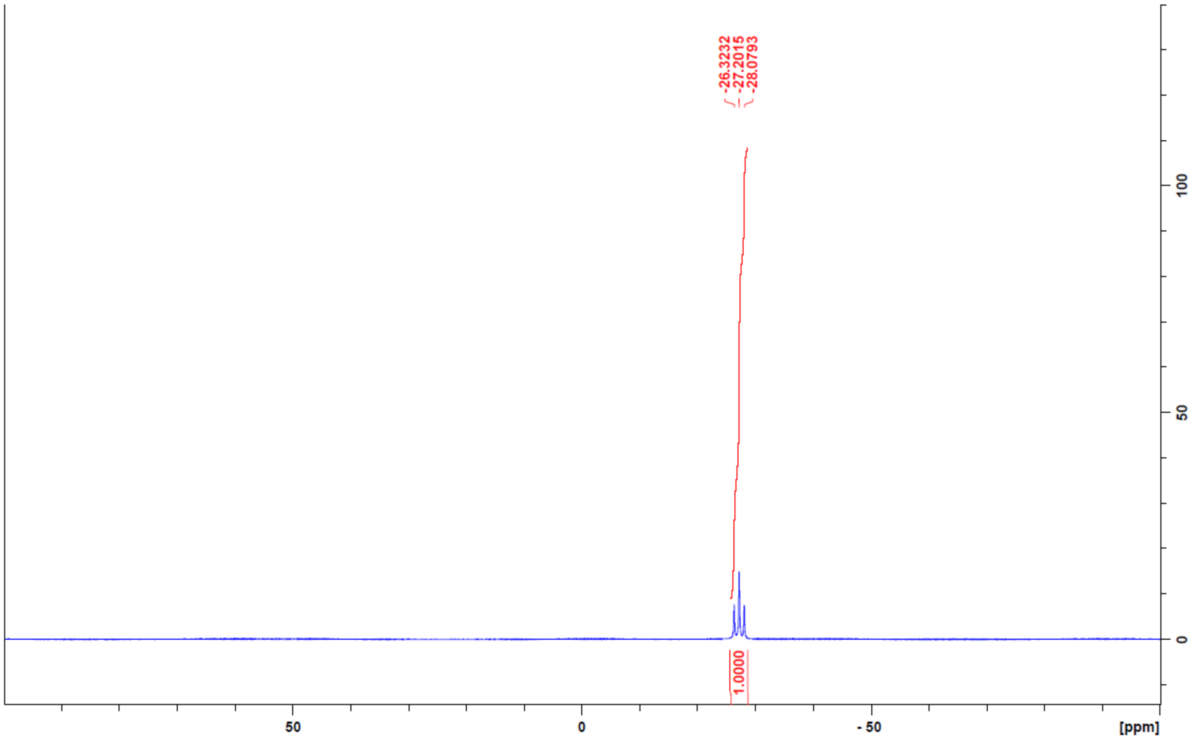

Figure S38. ^{11}B-NMR spectrum (C_6D_6, 96.2 MHz) of compound **15**

3. X-ray Crystallography

3.1. General Information

Data collections were performed by mounting single crystals on glass fibers or MiTeGen mounts in perfluorinated oil. Diffractometers used for intensity measurements (at 100 K) were Oxford Diffraction Xcalibur E with Mo K_{α} radiation or Rigaku XtaLAB Synergy S Single Source with either Mo K_{α} or Cu K_{α} micro sources. Absorption correction was applied based on multi-scan methods. Data reduction was performed using the program CrysalisPro.[5] The structures were solved with SHELXT-14/5.[6] and refined anisotropically on F^2 using the programs SHELXL-14/7 and SHELXL-17/1.[7]

3.2. Compound **6**.

CCDC code	1948988	
Empirical formula	$C_{19}H_{25}BN_4$	
Formula weight	320.24	
Temperature	100.0(1) K	
Wavelength	1.54184 Å	
Crystal system	Monoclinic	
Space group	*C*2/*c*	
Unit cell dimensions	a = 14.25640(10) Å	α = 90°
	b = 15.73740(10) Å	β = 92.0660(10)°
	c = 15.49490(10) Å	γ = 90°
Volume	3474.15(4) $Å^3$	
Z	8	
Density (calculated)	1.225 g/cm^3	
Absorption coefficient	0.566 mm^{-1}	
F(000)	1376	
Crystal size	0.350 × 0.300 × 0.050 mm^3	
Theta range for data collection	4.186 to 74.427°	
Index ranges	−17<=h<=16, −19<=k<=19, −19<=l<=19	
Reflections collected	72524	
Independent reflections	3557 [R(int) = 0.0470]	
Completeness to theta = 67.684°	100.0 %	
Absorption correction	Semi-empirical from equivalents	
Max. and min. transmission	1.00000 and 0.70601	
Refinement method	Full-matrix least-squares on F^2	
Data / restraints / parameters	3557 / 0 / 231	
Goodness-of-fit on F^2	1.078	
Final R indices [I>2sigma(I)]	R1 = 0.0359, wR2 = 0.0982	
R indices (all data)	R1 = 0.0383, wR2 = 0.1002	
Largest diff. peak and hole	0.205 and −0.168 e/$Å^3$	

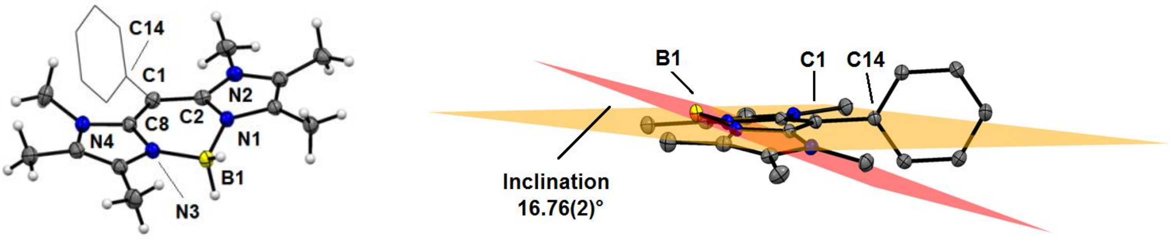

Figure S39. Molecular structure of compound **6**. The phenyl entity in the left illustration is represented in the wireframe model. Thermal ellipsoids are drawn at the 50 % probability level. Selected bond lengths [Å] and bond angles [°]:B1−N1 1.546(1), B1−N3 1.550(1), C8−N3 1.356(1), C1−C8 1.413(1), C1−C2 1.417(1), C1−C14 1.490(1), C2−N1 1.357(1), C8−C1−C14 121.95(9), C2−C1−C14 122.73(9), C2−C1−C8 115.17(9).

3.3. Compound **8**.

CCDC code	1948987	
Empirical formula	$C_{19}H_{24}N_4O$	
Formula weight	324.42	
Temperature	100.0(2) K	
Wavelength	1.54184 Å	
Crystal system	Monoclinic	
Space group	$P2_1/c$	
Unit cell dimensions	a = 10.1036(2) Å	α = 90°
	b = 20.5674(3) Å	β = 108.412(2)°
	c = 8.46620(10) Å	γ = 90°
Volume	1669.26(5) $Å^3$	
Z	4	
Density (calculated)	1.291 g/cm^3	
Absorption coefficient	0.652 mm^{-1}	
F(000)	696	
Crystal size	0.372 × 0.202 × 0.063 mm^3	
Theta range for data collection	4.299 to 76.297°	
Index ranges	−12<=h<=12, −25<=k<=25, −10<=l<=10	
Reflections collected	35841	
Independent reflections	3487 [R(int) = 0.0667]	
Completeness to theta = 67.684°	100.0 %	
Refinement method	Full-matrix least-squares on F^2	
Data / restraints / parameters	3487 / 0 / 227	
Goodness-of-fit on F^2	1.053	
Final R indices [I>2sigma(I)]	R1 = 0.0397, wR2 = 0.0899	
R indices (all data)	R1 = 0.0534, wR2 = 0.0975	
Largest diff. peak and hole	0.226 and −0.273 e/$Å^3$	

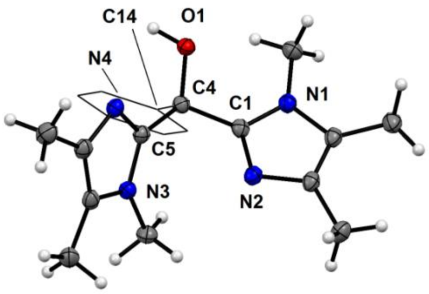

Figure S40. Molecular structure of compound **8**. The phenyl entity is represented in the wireframe model. Thermal ellipsoids are drawn at the 50 % probability level. Selected bond lengths [Å] and bond angles [°]: C1−C4 1.5167(19), C4−C5 1.5280(20), C4−C14 1.5377(18), C4−O1 1.4241(16), C1−C4−C5 109.90(11), C1−C4−C14 113.11(11), C5−C1−C14 109.12(11).

3.4. Compound **10**.

CCDC code	1948991	
Empirical formula	$C_{19}H_{26}BClN_4O \cdot CHCl_3$	
Formula weight	492.08	
Temperature	99.9(4) K	
Wavelength	0.71073 Å	
Crystal system	Monoclinic	
Space group	$P2_1/c$	
Unit cell dimensions	a = 17.2383(2) Å	α = 90°
	b = 12.8365(1) Å	β = 100.7650(10)°
	c = 22.4272(2) Å	γ = 90°
Volume	4875.34(8) $Å^3$	
Z	8	
Density (calculated)	1.341 g/cm^3	
Absorption coefficient	0.505 mm^{-1}	
F(000)	2048	
Crystal size	0.483 × 0.251 × 0.176 mm^3	
Theta range for data collection	2.560 to 27.482°.	
Index ranges	−22<=h<=22, −16<=k<=16, −29<=l<=29	
Reflections collected	209330	
Independent reflections	11172 [R(int) = 0.0531]	
Completeness to theta = 25.242°	99.9 %	
Absorption correction	Gaussian	
Max. and min. transmission	1.000 and 0.198	
Refinement method	Full-matrix least-squares on F^2	
Data / restraints / parameters	11172 / 0 / 571	
Goodness-of-fit on F^2	1.152	
Final R indices [I>2sigma(I)]	R1 = 0.0643, wR2 = 0.1481	
R indices (all data)	R1 = 0.0672, wR2 = 0.1495	
Largest diff. peak and hole	1.142 and −1.025 e/$Å^3$	

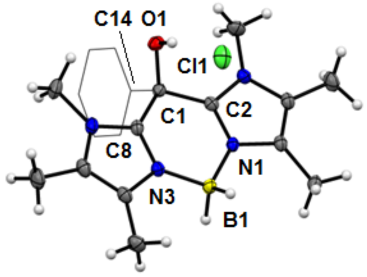

Figure S41. Molecular structure of compound **10**. The phenyl entity is represented in the wireframe model. Thermal ellipsoids are drawn at the 50 % probability level. Solvent molecules are omitted. Selected bond lengths [Å] and bond angles [°]: B1−N1 1.553(3), B1−N3 1.553(3), C8−N3 1.326(3), C1−C8 1.512(3), C1−C2 1.513(3), C1−C14 1.534(3), O1−C1 1.412(3), C2−N1 1.323(3), C8−C1−C14 107.90(18), C2−C1−C14 109.06(18), C2−C1−C8 108.56(18).

3.5. Compound **12**.

CCDC code	1948989	
Empirical formula	$C_{19}H_{28}B_2N_4$	
Formula weight	334.07	
Temperature	100.1(3) K	
Wavelength	0.71073 Å	
Crystal system	Monoclinic	
Space group	$P2_1/m$	
Unit cell dimensions	a = 8.1657(3) Å	α = 90°
	b = 13.3435(3) Å	β = 100.405(3)°
	c = 9.0392(3) Å	γ = 90°
Volume	968.71(5) Å^3	
Z	2	
Density (calculated)	1.145 g/cm^3	
Absorption coefficient	0.068 mm^{-1}	
F(000)	360	
Crystal size	0.250 × 0.200 × 0.150 mm^3	
Theta range for data collection	2.536 to 26.369°	
Index ranges	−10<=h<=10, −16<=k<=16, −11<=l<=11	
Reflections collected	46637	
Independent reflections	2077 [R(int) = 0.0576]	
Completeness to theta = 25.242°	99.9 %	
Absorption correction	Semi-empirical from equivalents	
Max. and min. transmission	1.00000 and 0.77710	
Refinement method	Full-matrix least-squares on F^2	
Data / restraints / parameters	2077 / 0 / 143	
Goodness-of-fit on F^2	1.054	
Final R indices [I>2sigma(I)]	R1 = 0.0429, wR2 = 0.1199	
R indices (all data)	R1 = 0.0490, wR2 = 0.1253	
Largest diff. peak and hole	0.289 and −0.158 e/Å^3	

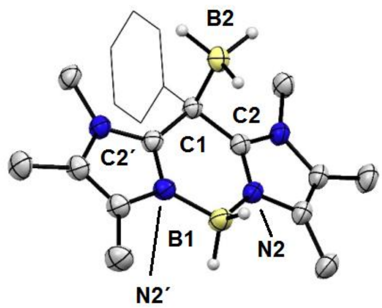

Figure S42. Molecular structure of compound **12**. The phenyl entity is represented in the wireframe model. Thermal ellipsoids are drawn at the 50 % probability level. Carbon bound hydrogen atoms are omitted. The illustrated molecule is located on a crystallographic mirror plane including B1, C1, B2 and the phenyl entity. Selected bond lengths [Å] and bond angles [°]: B1−N2 1.5443(16), C1−C2 1.4975(14), C2−N2 1.3330(15), C1−B2 1.679(2), C2−C1−C2´ 108.16(13), C2−C1−B2 106.34(9), N2−B1−N2´ 105.03(14).

3.6. Compound **14**

CCDC code	1948990	
Empirical formula	$C_{28}H_{38}B_2N_4$	
Formula weight	452.24	
Temperature	100.00(10) K	
Wavelength	1.54184 Å	
Crystal system	Monoclinic	
Space group	$P2_1/c$	
Unit cell dimensions	a = 14.1625(2) Å	α = 90°
	b = 10.6880(2) Å	β = 99.644(2)°
	c = 16.9419(3) Å	γ = 90°
Volume	2528.23(8) $Å^3$	
Z	4	
Density (calculated)	1.188 g/cm^3	
Absorption coefficient	0.525 mm^{-1}	
F(000)	976	
Crystal size	0.150 × 0.100 × 0.050 mm^3	
Theta range for data collection	3.165 to 77.862°	
Index ranges	−17<=h<=17, −13<=k<=13, −18<=l<=21	
Reflections collected	47356	
Independent reflections	5315 [R(int) = 0.1119]	
Completeness to theta = 67.684°	100.0 %	
Absorption correction	Semi-empirical from equivalents	
Max. and min. transmission	1.00000 and 0.33135	
Refinement method	Full-matrix least-squares on F^2	
Data / restraints / parameters	5315 / 0 / 354	
Goodness-of-fit on F^2	1.087	
Final R indices [I>2sigma(I)]	R1 = 0.0443, wR2 = 0.1209	
R indices (all data)	R1 = 0.0532, wR2 = 0.1278	
Largest diff. peak and hole	0.270 and −0.256 e/$Å^3$	

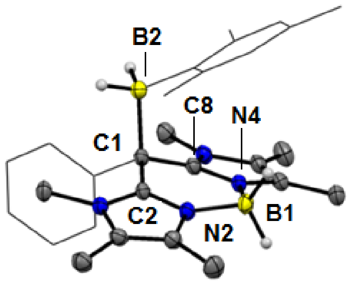

Figure S43. Molecular structure of compound **14**. The phenyl and the mesityl entity are represented in the wireframe model. Thermal ellipsoids are drawn at the 50 % probability level. Carbon bound hydrogen atoms are omitted. Selected bond lengths [Å] and bond angles [°]: B1−N2 1.5519(17), B1−N4 1.5543(18), B2−C1 1.734(2), C2−N2 1.3368(16), C8−N4 1.3340(16), C1−C2 1.5033(18), C1−C8 1.4989(17), N2−B1−N4 104.24(10), C8−C1−C2 107.40(10).

4. NMR Experiments

The decomplexation reactions of compounds **12** and **14** with the NHC IMe^{Me} were studied with ^{1}H- and $^{11}B\{^{1}H\}$-NMR experiments. By way of example we present $^{11}B\{^{1}H\}$-NMR spectra obtained from the reaction of **14** with the NHC IMe^{Me} in comparison to compounds **6** and **15** being formed.

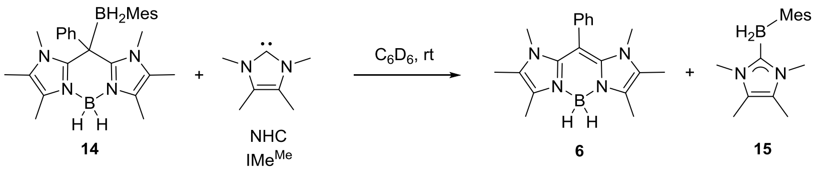

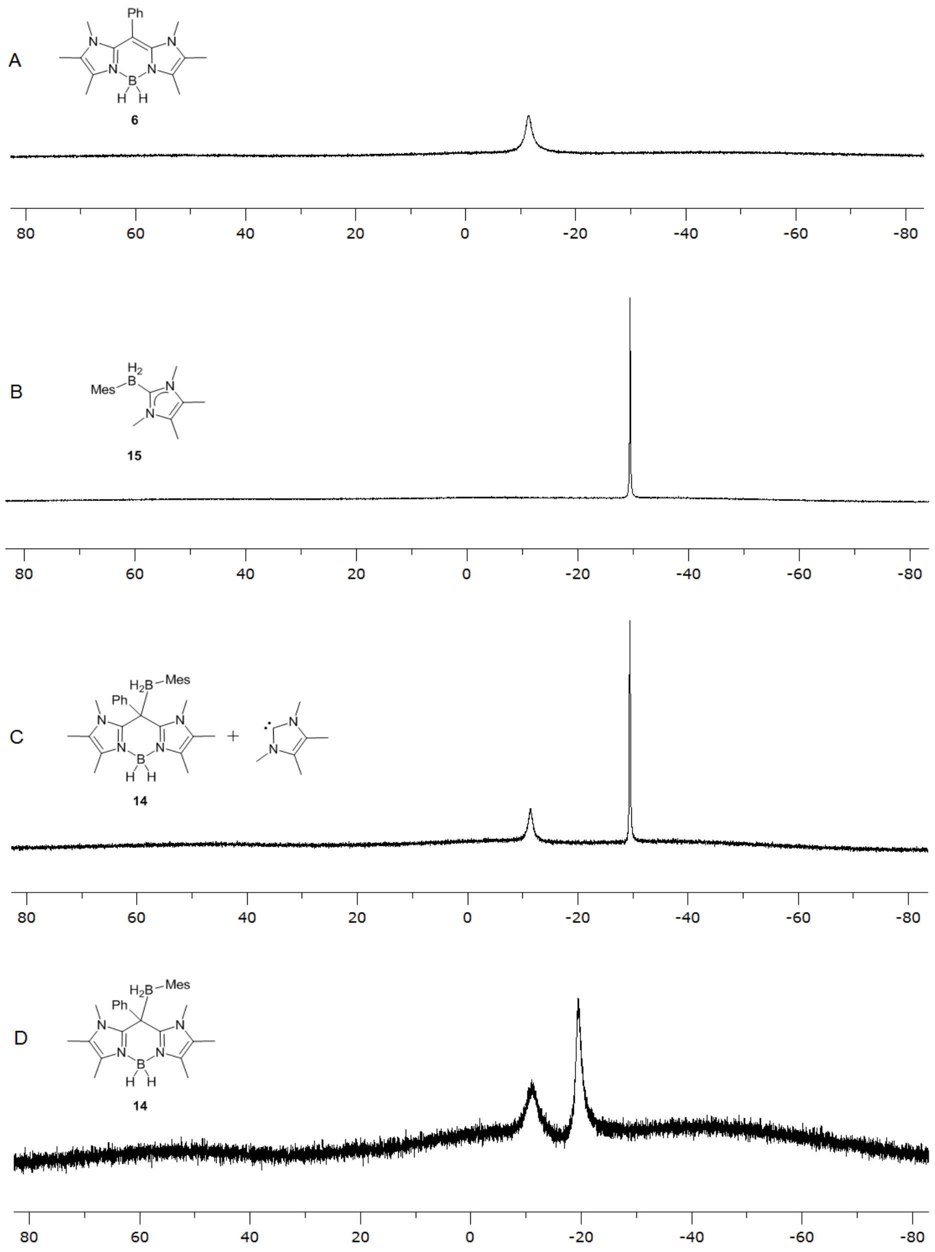

Figure S44. Chart A: Compound **6**. Chart B: Compound **15**. Chart C: A stoichiometric mixture of **14** and the NHC IMe^{Me} shows the quantitative formation of **6** (Chart A) and **15** (Chart B). Chart D: Compound **14**. Conditions for all spectra: C_6D_6, 96.2 MHz, 293 K, axis scaled in ppm.

5. Quantum Chemical Calculations

5.1 Computational Details

For the calculation of absorption and emission spectra, the Gaussian16 program[8] was used. Solvation effects were described within the polarizability continuum model[9,10] (PCM) using default settings for the cavity construction and solvent parameters. For the absorption spectra, the initial structure optimizations of reference **B** and **6** were performed using the def2-TZVP basis set[11] and the PBE0[12] exchange-correlation (XC) functional, which was previously found to be reliable for ground state (GS) structure optimization of molecules from the BODIPY class.[13] In order to validate the robustness of the computed metrics, ground state optimizations of **6** were repeated in ORCA[14] with the BP86[15] and B3LYP [16] functionals and TZVP basis sets (Table S1); consistent results were obtained largely independent of the level of theory. Optimized structures were verified to be local minima by calculation of harmonic vibrational frequencies. Excitation energies and oscillator strengths were calculated using linear-response time-dependent density functional theory (TD-DFT) using the long-range-corrected hybrid XC functional CAM-B3LYP.[17] For exploitation of an empirical correction based on benchmark results by Momeni *et al.,*[18] i.e.

$$\Delta E_{\text{corrected}} = 1.0941 \cdot \Delta E_{\text{CAM-B3LYP}} - 0.8659\ \text{eV}\ ,$$

the cc-pVTZ basis set[19] was used for this step. A total of 30 vertical excitation energies were calculated. Plotted spectra were broadened using Gaussian line shapes with a half-width at half maximum (HWHM) of 0.25 eV (≈ 2000 cm^{-1}).

For the emission spectra, structure optimizations of the first (singlet) excited-state (ES) were performed using the wB97XD XC functional[20] and def2-TZVP basis set. Optimized structures were verified to be ES local minima by calculation of ES harmonic vibrational frequencies. The calculated emission energies were corrected for the state-specific solvation[21] of the ES, i.e. the ES electrostatic potential was made self-consistent with the solvent reaction field. Since the emission process is fast compared to molecular motion, only the polarization of the solvents charges was accounted for in this step, i.e. non-equilibrium solvation was used. Note that empirical XC functional corrections (see above) could not be applied for the emission spectrum as these corrections were deduced for transitions at GS structures.

Different from the ground state structure of **6**, having the arene tilted from orthogonality by ca. 20° (XRD and DFT), the excited state structure converged to (close-to-ideal) C_{2v} symmetry in THF and hexanes with close-to-orthogonal π-systems (dihedral τ = 88.5° and 89.7° in THF and hexanes, respectively). Transitions between ES and GS are hindered by symmetry in this geometry in THF (f_{osc} = 0.0047), whereas they are essentially forbidden in hexanes (f_{osc} < 0.00005). Single-point calculations in the structure minima therefore cannot and do not resemble the experimentally found fluorescence intensities. By contrast, experimental emission spectra in hexanes and THF are convincingly matched by theory, if vibrationally excited states are taken into account which deviate from C_{2v} symmetry. The oscillator strengths (corresponding to the probability and intensity of the radiative transition) were in fact found to be crucially dependent on the dihedral angle τ between the phenyl ring and the BODIIM backbone. Finite temperature effects (e.g. molecular vibrations) lead to an ensemble of conformers with different dihedral angles more or less favoring the emission process. More complete Boltzmann

weighed averaging of spectra could be used in principle to receive the correct oscillator strengths. Given the quite significant computational cost of a proper Born-Oppenheimer molecular dynamics sampling of several hundred conformers, we restricted ourselves to a zero-order approximation along a tailor-made reaction coordinate. We manually sampled ~15 conformers by modifying the dihedral angles between the BODIIM backbone and the phenyl ring within a 75°-105° range (concomitant with the dihedral angles of the adjacent N-Me groups). All other internal coordinates were fixed at their global ES minimum values. A Boltzmann weighed (T = 300 K) averaging of the obtained Gaussian broadened (HWHM = 0.25 eV) single-point spectra was then used to obtain the final emission spectra in both *n*-hexane and THF. Additionally, the obtained ES and GS energies were plotted against the dihedral angle and an analogous scan at the GS structure was performed at the PBE0/ def2-TZVP level of theory for comparison.

5.2 Results

Table S1. Selected bond lengths in Å (numbers in parentheses denote the deviation among equivalent sites) of optimized ground-state (**6** and **B**) and excited-state structures of compound **6**.

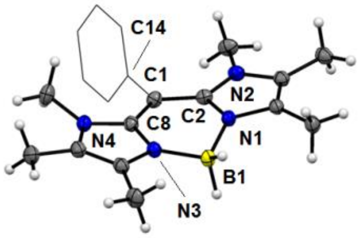

	Ground state					Excited state	
	BP86[a]	B3LYP[a]		PBE0[b]	CAM-B3LYP[b]	CAM-B3LYP[b,c]	wB97XD[b,c]
	**6**	**6**	**B**	**6**	**6**	**6**	**6**
B1-N1	1.556(2)	1.552(4)	1.547(2)	1.546(1)	1.546(2)	1.548	1.549
N1-C2	1.369(2)	1.359(3)	1.400(1)	1.348(1)	1.352(4)	1.345	1.344
N1-C_{im}	1.396(1)	1.395(1)	1.352(2)	1.380(1)	1.384(1)	1.366	1.365
N4-C8	1.381(1)	1.371(1)	-	1.366(1)	1.365(1)	1.356	1.355
N4-$C_{im'}$	1.411(1)	1.409(1)	-	1.397(1)	1.403(2)	1.377	1.377
C2-C1	1.421(2)	1.417(1)	1.398(1)	1.416(1)	1.415(1)	1.426	1.428
C1-C14	1.482	1.490	1.488	1.479	1.487	1.447	1.441
C14-C15	1.410(1)	1.400(1)	1.394(2)	1.399(1)	1.394(1)	1.441	1.443
C15-C16	1.396(1)	1.391(1)	1.390(2)	1.387(1)	1.385(1)	1.368	1.369
C16-C17	1.400(1)	1.393(1)	1.397(2)	1.388(1)	1.385(1)	1.412	1.414

[a] in THF; [b] in hexanes; [c] converged to C_{2V} symmetric structure.

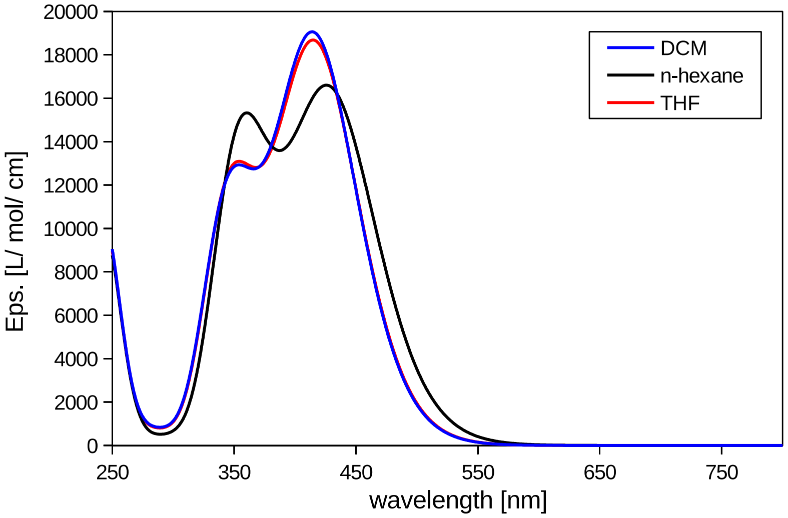

Figure S45. Calculated absorption spectra of **6** using empirically corrected CAM-B3LYP/cc-pVDZ TD-DFT excitation energies at PBE0/def2-TZVP structures and a Gaussian broadening of FWHM = 0.25eV.

Calculated absorption spectra for **6** (Figure S45) are generally in good agreement with experiment. The maxima of the absorption bands at 354 nm and 415 nm (in THF) almost perfectly coincide with the experimental absorption maxima at 360 nm and 400 nm. For calculations in DCM, the shape of the calculated bands is in decent agreement with the experimental spectrum. Calculated results in THF are almost identical to calculated DCM results. This is in line with the expectation since THF and DCM have comparable dielectric constants (THF: 7.426, DCM: 8.930) and refractive indices (THF: 1.974, DCM: 2.028) leading to very similar results within the polarizability continuum model. For calculations in *n*-hexane, the experimentally observed increase of the band at ca. 360 nm (calculated: 360 nm) relative to the band at ca. 400 nm (calculated: 426 nm) is reproduced. Note, however, that the distinct decrease of overall absorbance in the experimental *n*-hexane spectrum is less pronounced in the calculated spectrum.

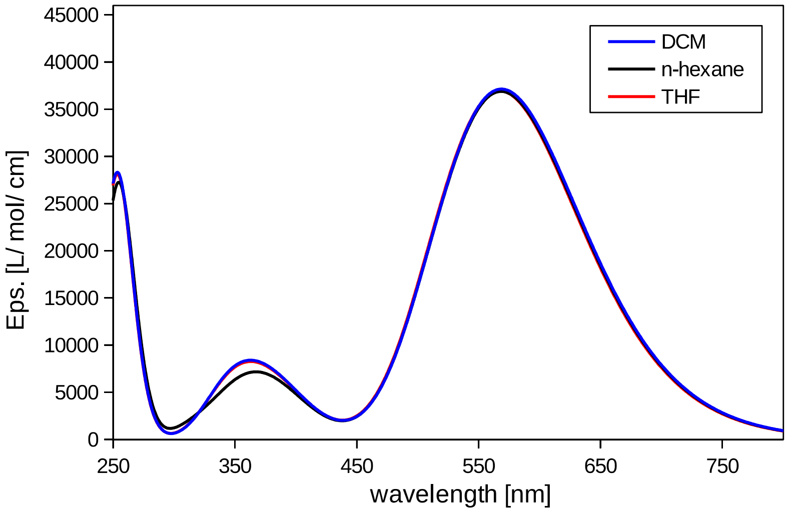

Figure S46. Calculated absorption spectra of **B** using empirically corrected CAM-B3LYP/cc-pVDZ TD-DFT excitation energies at PBE0/def2-TZVP structures and a Gaussian broadening of FWHM = 0.25eV.

The calculated absorption maxima of **B** (Figure S46; *n*-hexane: 367 nm, 568 nm) are in very good agreement with the experimental values found by Bröring *et al.* (toluene: 378 nm, 526 nm).[22] These results also support the validity of the computational procedure and the empirical correction applied.

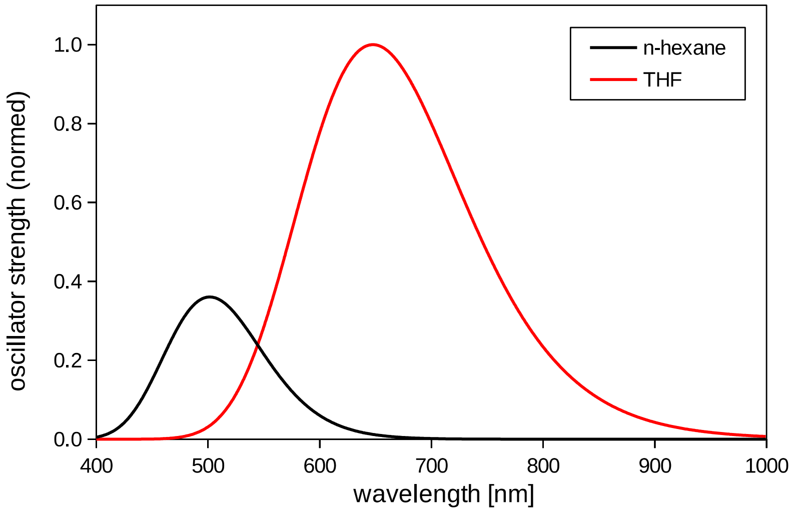

Figure S47. Calculated emission spectra of **6** at wB97XD/def2-TZVP TD-DFT level of theory. Oscillator strengths were normed to 1.0 at λ_{max} in THF.

The calculated emission maximum of **6** is 502 nm in *n*-hexane (648 nm in THF) and thus in excellent (reasonable) agreement with the experimental maximum of 480 nm in *n*-hexane (530 nm in THF). The solvatochromic shift is qualitatively well reproduced. Note that the state-specific equilibration of the solvent at the ES structure is critical and uncorrected emission energies show a reversed solvatochromic shift.

Table S2. Results from scan along dihedral angle τ in *n*-hexane. Bold line corresponds to ES minimum.

τ	f(osc)	E(GS) /Ha	E(ES)/Ha	ΔE / eV	Boltzmann weight	weighted f(osc)
**74.7**	0.0607	-983.03655	-982.9382	2.68	0.000010	0.000001
**77.7**	0.0403	-983.03691	-982.94131	2.60	0.000274	0.000011
**80.7**	0.0234	-983.03702	-982.94386	2.54	0.004001	0.000094
**82.7**	0.0144	-983.03702	-982.94526	2.50	0.017500	0.000252
**84.7**	0.0075	-983.03699	-982.94638	2.47	0.057146	0.000429
**86.7**	0.0027	-983.03697	-982.94713	2.44	0.125256	0.000338
**88.7**	0.0003	-983.03696	-982.9475	2.43	0.186173	0.000056
**89.7**	**0.0000**	**-983.03696**	**-982.94759**	**2.43**	**0.204221**	**0.000000**
**90.7**	0.0003	-983.03696	-982.94755	2.43	0.194843	0.000058
**92.7**	0.0026	-983.03699	-982.94714	2.44	0.127225	0.000331
**94.7**	0.0073	-983.03703	-982.94642	2.47	0.059261	0.000433
**96.7**	0.0141	-983.03706	-982.94534	2.50	0.019077	0.000269
**98.7**	0.0229	-983.03705	-982.94391	2.53	0.004221	0.000097
**100.7**	0.0336	-983.03698	-982.94228	2.58	0.000758	0.000025
**103.7**	0.0527	-983.03667	-982.93933	2.65	0.000034	0.000002
**106.7**	0.0746	-983.03599	-982.93595	2.72	0.000001	0.000000

Table S3. Results from scan along dihedral angle τ in THF. Bold line corresponds to ES minimum.

τ	f(osc)	E(GS) /Ha	E(ES)/Ha	ΔE / eV	Boltzmann weight	weighted f(osc)
**74.5**	0.0508	-983.03569	-982.95516	2.19	0.000005	0.000000
**77.5**	0.0341	-983.03573	-982.95871	2.10	0.000203	0.000007
**80.5**	0.0205	-983.03556	-982.96178	2.01	0.005121	0.000105
**82.5**	0.0135	-983.03542	-982.96343	1.96	0.029366	0.000396
**84.5**	0.0084	-983.03528	-982.96469	1.92	0.109572	0.000920
**86.5**	0.0054	-983.03518	-982.96546	1.90	0.247041	0.001334
**88.5**	**0.0047**	**-983.03515**	**-982.96562**	**1.89**	**0.294017**	**0.001382**
**90.5**	0.0062	-983.03521	-982.96530	1.90	0.209252	0.001297
**92.5**	0.0098	-983.03533	-982.96441	1.93	0.082284	0.000806
**94.5**	0.0155	-983.0355	-982.96307	1.97	0.019909	0.000309
**96.5**	0.0231	-983.03566	-982.96130	2.02	0.003109	0.000072
**99.5**	0.0374	-983.03582	-982.95819	2.11	0.000117	0.000004
**102.5**	0.0545	-983.03572	-982.95456	2.21	0.000003	0.000000

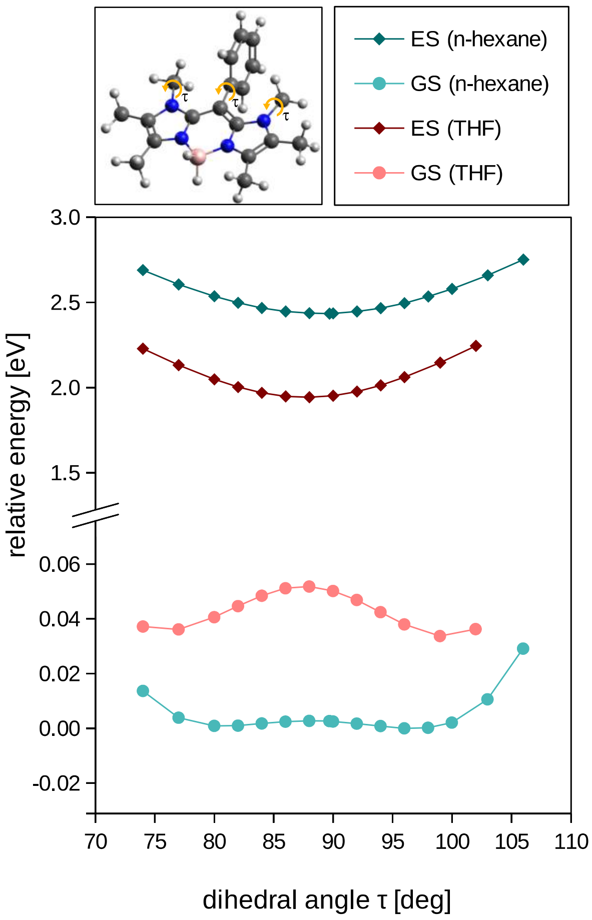

Figure S48. GS and ES relative energies (wB97XD/ def2-TZVP, state-specific non-eq. solvation) along the dihedral angle indicated in the insert. All other parameters were fixed at their ES minimum values.

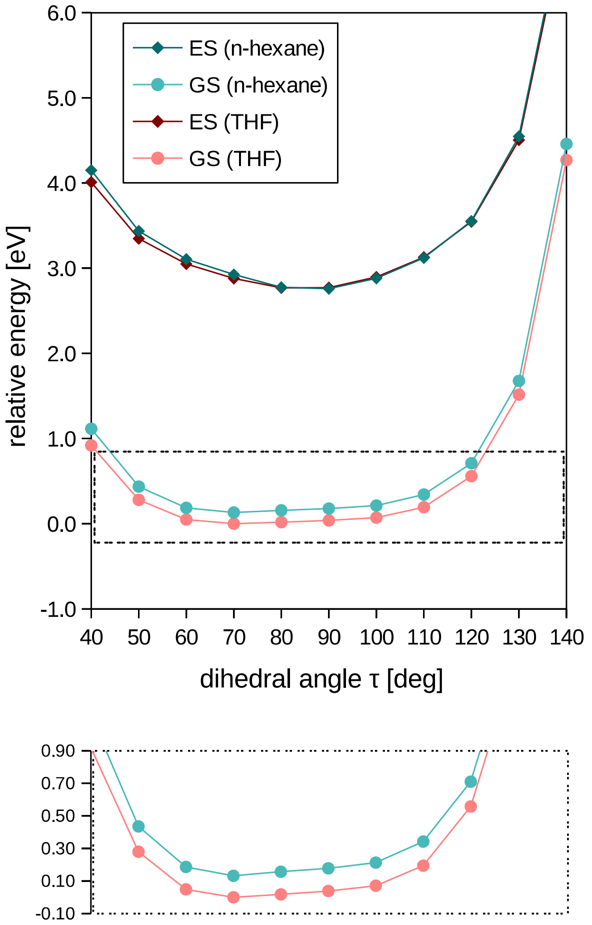

Figure S49. GS and ES relative energies (PBE0/ def2-TZVP, non-eq. solvation) along the dihedral angle. All other parameters were fixed at their GS minimum values.

6. References

[1] X. Zhou, Y. Gong, *Eur. J. Org. Chem.* **2011**, 6092–6099.
[2] A. Solovyev, S.-H. Ueng, J. Monot, L. Fensterbank, M. Malacria, E. Lacôte, D. P. Curran, *Organic Lett.* **2010**, *12*, 2998−3001.
[3] F. Hanasaka, K. Fujita, R. Yamaguchi, *Organometallics* **2005**, *24*, 3422−3433.
[4] K. Smith, A. Pelter, Z. Jin, *Angew. Chem. Int. Ed.* **1994**, *33*, 851−853.
[5] Rigaku Oxford Diffraction, "CrysAlisPRO Software System version 1.171.39.46", **2018.** Rigaku Corporation, Oxford, UK. (Ed.).
[6] G. M. Sheldrick, *Acta Cryst.* **2015**, *A71*, 3–8.
[7] G. M. Sheldrick, *Acta Crystallogr., Sect. A: Found. Crystallogr.* **2008**, *64*, 112–122.
[8] Gaussian 16 Revision A.03, M. J. Frisch, G. W. Trucks, H. B. Schlegel, G. E. Scuseria, M. A. Robb, J. R. Cheeseman, G. Scalmani, V. Barone, G. A. Petersson, H. Nakatsuji, X. Li, M. Caricato, A. V. Marenich, J. Bloino, B. G. Janesko, R. Gomperts, B. Mennucci, H. P. Hratchian, J. V. Ortiz, A. F. Izmaylov, J. L. Sonnenberg, D. Williams- Young, F. Ding, F. Lipparini, F. Egidi, J. Goings, B. Peng, A. Petrone, T. Henderson, D. Ranasinghe, V. G. Zakrzewski, J. Gao, N. Rega, G. Zheng, W. Liang, M. Hada, M. Ehara, K. Toyota, R. Fukuda, J. Hasegawa, M. Ishida, T. Nakajima, Y. Honda, O. Kitao, H. Nakai, T. Vreven, K. Throssell, J. A. Montgomery, Jr., J. E. Peralta, F. Ogliaro, M. J. Bearpark, J. J. Heyd, E. N. Brothers, K. N. Kudin, V. N. Staroverov, T. A. Keith, R. Kobayashi, J. Normand, K. Raghavachari, A. P. Rendell, J. C. Burant, S. S. Iyengar, J. Tomasi, M. Cossi, J. M. Millam, M. Klene, C. Adamo, R. Cammi, J. W. Ochterski, R. L. Martin, K. Morokuma, O. Farkas, J. B. Foresman, D. J. Fox, Gaussian Inc. Wallingford CT, 2016.
[9] S. Miertuš, E. Scrocco, J. Tomasi, *Chem. Phys.* **1981**, *55*, 117–129.
[10] J. Tomasi, B. Mennucci, R. Cammi, *Chem. Rev.* **2005**, *105*, 2999–3094.
[11] F. Weigend, R. Ahlrichs, *Phys. Chem. Chem. Phys.* **2005**, *7*, 3297–3305.
[12] C. Adamo, V. Barone, *J. Chem. Phys.* **1999**, *110*, 6158–6170.
[13] B. Le Guennic, O. Maury, D. Jacquemin, *Phys. Chem. Chem. Phys.* **2012**, *14*, 157–164.
[14] F. Neese, *WIREs Comput. Mol. Sci*. **2012**, *2*, 73-78.
[15] A. D. Becke, A. *J. Chem. Phys*., **1993** *98*, 5648–5652.
[16] A. D. Becke, *Phys. Rev. A* **1988**, *38*, 3098–3100.
[17] T. Yanai, D. P. Tew, N. C. Handy, *Chem. Phys. Lett.* **2004**, *393*, 51–57.
[18] M. R. Momeni, A. Brown. *J Chem. Theory Comput.* **2015**, *11*, 2619–2632.
[19] T. H. Dunning Jr., *J. Chem. Phys.* **1989**, *90*, 1007–1023.
[20] J.-D. Chai, M. Head-Gordon, *Phys. Chem. Chem. Phys.* **2008**, *10*, 6615–6620.
[21] R. Improta, V. Barone, G. Scalmani, M. J. Frisch, *J. Chem. Phys*. **2006**, *125*, 054103.
[22] M. Bröring, R. Krüger, S. Link, C. Kleeberg, S. Köhler, X. Xie, B. Ventura, L. Flamigni, *Chem. Eur. J.* **2008**, *14*, 2976–2983.
